# Predicting drug approvals: The Novartis data science and artificial intelligence challenge

**DOI:** 10.1016/j.patter.2021.100312

**Published:** 2021-07-21

**Authors:** Kien Wei Siah, Nicholas W. Kelley, Steffen Ballerstedt, Björn Holzhauer, Tianmeng Lyu, David Mettler, Sophie Sun, Simon Wandel, Yang Zhong, Bin Zhou, Shifeng Pan, Yingyao Zhou, Andrew W. Lo

**Affiliations:** 1 Laboratory for Financial Engineering, Sloan School of Management, Massachusetts Institute of Technology, Cambridge, MA 02142, USA; 2 Department of Electrical Engineering and Computer Science, Massachusetts Institute of Technology, Cambridge, MA 02142, USA; 3 Novartis Pharma AG, 4056 Basel, Switzerland; 4 Novartis Pharmaceuticals Corporation, East Hanover, NJ 07936, USA; 5 Genomics Institute of the Novartis Research Foundation, San Diego, CA 92121, USA; 6 Sante Fe Institute, Santa Fe, NM 87501, USA

**Keywords:** clinical trials, prediction, data science, artificial intelligence, XGBoost, probability of success

## Abstract

We describe a novel collaboration between academia and industry, an in-house data science and artificial intelligence challenge held by Novartis to develop machine-learning models for predicting drug-development outcomes, building upon research at MIT using data from Informa as the starting point. With over 50 cross-functional teams from 25 Novartis offices around the world participating in the challenge, the domain expertise of these Novartis researchers was leveraged to create predictive models with greater sophistication. Ultimately, two winning teams developed models that outperformed the baseline MIT model—areas under the curve of 0.88 and 0.84 versus 0.78, respectively—through state-of-the-art machine-learning algorithms and the use of newly incorporated features and data. In addition to validating the variables shown to be associated with drug approval in the earlier MIT study, the challenge also provided new insights into the drivers of drug-development success and failure.

## Highlights

• Data science challenge at Novartis in collaboration with MIT researchers
• Dataset derived from 2 decades of drug development and clinical trial data
• Winning teams outperformed the baseline MIT model by leveraging domain expertise
• Predictive analytics can augment human judgment in drug-development risk management

## The bigger picture

The probability of success is a key parameter that clinical researchers, biopharma executives and investors, and portfolio managers focus on when making important scientific and business decisions about drug development. We describe an in-house data science and artificial intelligence challenge organized by Novartis in collaboration with MIT researchers. Using state-of-the-art machine-learning algorithms and extensive feature engineering augmented by domain expertise in drug development, two winning teams developed models that outperformed the baseline MIT model proposed in a prior study. These new predictive models can be used to augment human judgment to make more informed data-driven decisions in portfolio risk management and capital allocation. These results suggest the possibility of developing even more accurate models using more comprehensive and informative data, and a broader pool of challenge participants.

## Teaser

In an in-house data science challenge, Novartis researchers developed machine-learning models for predicting drug-development outcomes, using 2 decades of clinical trial data and building upon previous work at MIT. By leveraging domain expertise, 2 winning teams, out of 50 that participated, developed models that outperformed the baseline MIT model through state-of-the-art algorithms and feature engineering. In addition to providing new insights into drug approvals, the models can augment human judgment to make more informed risk-management decisions.

## Introduction

The rising cost of clinical trials and a shift to utilizing more complex biological pathways with greater therapeutic potential—but also greater chances of failure—have caused drug development to become an increasingly lengthy, costly, and risky endeavor in the past decade.^1^, ^2^, ^3^, ^4^, ^5^ The average drug now requires at least 10 years of translational research involving multiple iterations of lead optimization and several phases of clinical studies costing hundreds of millions of dollars before it can be approved by drug-regulatory authorities, such as the US Food and Drug Administration (FDA).

Due to the capital-intensive nature of the drug-development process, biotech and pharma companies can only afford to invest in a limited number of projects. When managing their portfolios of investigational drugs, these developers typically use historical estimates of regulatory approval rates, based on the therapeutic class and phase of development of the drug, combined with subjective adjustments, determined through unstructured discussions of project-specific risk factors, to make their investment decisions. Recently, however, there has been increased interest in combining machine-learning predictions with human judgments on project-specific information in a more structured manner.^6^

In a recent large-scale study involving a range of drug and clinical trial features from over 6,000 unique drugs and close to 20,000 clinical trials, Lo et al.^7^ applied machine-learning techniques to predict regulatory approval. Using two proprietary pharmaceutical pipeline database snapshots (taken through 2015Q4) provided by Informa (Pharmaprojects and Trialtrove), Lo et al.^7^ developed models that achieved promising predictive accuracy, measured at 0.78 and 0.81 AUC for predicting transitions from phase 2 to regulatory approval and phase 3 to regulatory approval, respectively. (The AUC, also known as the area under the receiver-operating characteristic curve, is the estimated probability that a classifier will rank a positive outcome higher than a negative outcome.) The models also identified the most useful features for predicting drug-development outcomes: trial outcome, trial status, trial accrual, trial duration, prior approval for another indication, and sponsor track record.

With a better understanding of the drivers of drug approval as well as more accurate forecasts of the likelihood of clinical trial success, biopharma companies and investors should be better able to assess the risks of different drug-development projects, and thus allocate their capital more efficiently.

As an extension of the previous study, the authors (all from MIT) collaborated with Novartis, one of the largest multinational pharmaceutical companies in the world, to implement an in-house data science and artificial intelligence (DSAI) challenge based on updated snapshots (taken through 2019Q1) of the same Informa databases. This challenge was designed to leverage the domain expertise of Novartis data scientists, statisticians, portfolio managers, and researchers to develop more powerful models for predicting the probability of success of pipeline drug candidates and uncover deeper insights into the drivers of drug approval. Success in this context was defined as regulatory approval. Over 50 teams consisting of more than 300 individuals from 25 Novartis offices around the world participated in the challenge, submitting approximately 3,000 models for evaluation in a head-to-head competition. In addition to their predictive performance, the teams were evaluated on the innovativeness and robustness of their models, and the potential business value of their findings.

In this paper, we summarize the findings of the top-performing teams. By examining their models, we validate the variables previously found to be associated with drug approval and identify new features that contain useful signals about drug-development outcomes.

### Methods

#### Data

For the DSAI challenge, we used two pharmaceutical pipeline databases from the commercial data vendor Informa for the core dataset: Pharmaprojects, which specializes in drug information, and Trialtrove, which specializes in clinical trial intelligence (see https://pharmaintelligence.informa.com/products-and-services/data-and-analysis/citeline). These two databases aggregate drug and trial information from over 40,000 data sources in the public domain, including company press releases, government drug and trial databases (e.g., Drugs@FDA and ClinicalTrials.gov), and scientific conferences and publications. The database snapshots used in this paper are updated versions of those used in Lo et al.^7^ (2019Q1 versus 2015Q4).

As in Lo et al.,^7^ we constructed a dataset of drug-indication pairs, focused on phase 2 trial data that have known outcomes (“P2APP”), either successful registration or program termination. We extracted a range of drug compound attributes and clinical trial characteristics as potential features for prediction, including three binary features, one date, seven numerical features, two multi-class features, 16 multi-label features, and five unstructured free texts. These are summarized in Table 1. For the purpose of our analysis, we defined the development status of suspension, termination, and lack of development as “failures,” and registration and launch in at least one country as “successes” or approvals (see Note S1 for further details).

**Table 1:** Features extracted from Pharmaprojects and Trialtrove

	Description
**Drug-indication pair**	

Biological target	protein on which the drug acts
Country	country in which the drug is being developed
Drug-indication development status	current approval status of the drug-indication pair
Indication	indication for which the drug is under development
Mechanism of action	mechanism through which the drug produces its pharmacological effect
Medium	physical composition of the material in which the drug is contained
Name	name of the drug
Origin	origin of the active ingredient in the drug
Prior approval of drug for another indication	approval of the drug for another indication prior to the indication under consideration
Route	route by which the drug is administered
Therapeutic class	therapy area for which the drug is in development

**Trial**	

Attribute	distinguishing attribute or feature of the trial, e.g., registration trials, biomarkers, immuno-oncology
Actual accrual	number of patients enrolled in the trial
Disease type	disease, disorder, or syndrome studied in the trial
Duration	duration of the trial
Exclusion criteria	criteria for excluding a patient from trial consideration
Gender	gender of the enrolled patients
Investigator experience	primary investigator's success in developing other drugs prior to the drug-indication pair under consideration
Location	country in which the trial is conducted
Number of identified sites	number of sites where the trial is conducted
Outcome	outcome of the trial
Patient age	minimum and maximum age of the enrolled patients
Patient population	general information about the disease condition of the enrolled patients
Patient segment	disease segmentation by patient subtypes, therapeutic objectives, or disease progression/staging
Phase 2 end date	year phase 2 ended (end date of the last observed phase 2 trial)
Primary endpoint	detailed description of primary objective, endpoint, or outcome of the trial; endpoints are classified into four main groups: efficacy, safety/toxicity, health economics and outcomes research, and pharmacokinetics/pharmacodynamics
Sponsor	financial sponsor of the trial
Sponsor track record	sponsor's success in developing other drugs prior to the drug-indication pair under consideration
Sponsor type	sponsor grouped by type
Status	recruitment status of the trial
Design	investigative methods used in the trial
Design keywords	keywords relating to investigative methods used in the trial
Target accrual	number of patients sought for the trial
Therapeutic area	therapeutic area of the disease studied in the trial

See Note S1 for examples of each feature.

The resulting dataset consisted of 6,901 drug-indication pairs and 12,680 unique phase 2 clinical trials, with end dates spanning 1999 to early 2019, containing about two decades of data (Table 2). In our dataset, 796 drug-indication pairs (11.5%) were successes, and 6,105 drug-indication pairs (88.5%) ended in failure. The data cover 15 indication groups: alimentary, anti-cancer, anti-infective, anti-parasitic, blood and clotting, cardiovascular, dermatological, genitourinary, hormonal, immunological, musculoskeletal, neurological, rare diseases, respiratory, and sensory products. Drugs for cancer, rare diseases, and neurological diseases made up the largest subgroups. As expected, the majority of the trials in the dataset were sponsored by industry rather than investigator-initiated academic trials.

**Table 2:** Sample sizes of the P2APP dataset, and the training and testing data used for the challenge

	Drug-indication pairs	Clinical trials	Unique drugs	Unique indications	Unique clinical trials
**Phase 2 to approval (P2APP)**					

Success	796	2,435	614	182	2,209
Failure	6,105	13,203	3,313	283	10,722
Total	6,901	15,638	3,726	291	12,680

**Training data**					

Success	610	1,852	468	169	1,666
Failure	4,293	6,839	2,537	264	5,845
Total	4,903	8,691	2,872	272	7,451

**Testing data**					

Success	186	583	160	93	557
Failure	1,812	6,364	1,096	218	5,065
Total	1,998	6,947	1,229	229	5,561

Note that the number of unique drugs, indications, and clinical trials are not necessarily additive across rows since drugs, indications, and trials have relationships that are surjective and non-injective: different drugs may target the same indication, and some trials may involve multiple drug-indication pairs. See also Note S1.

#### Challenge setup

**Figure 1:**
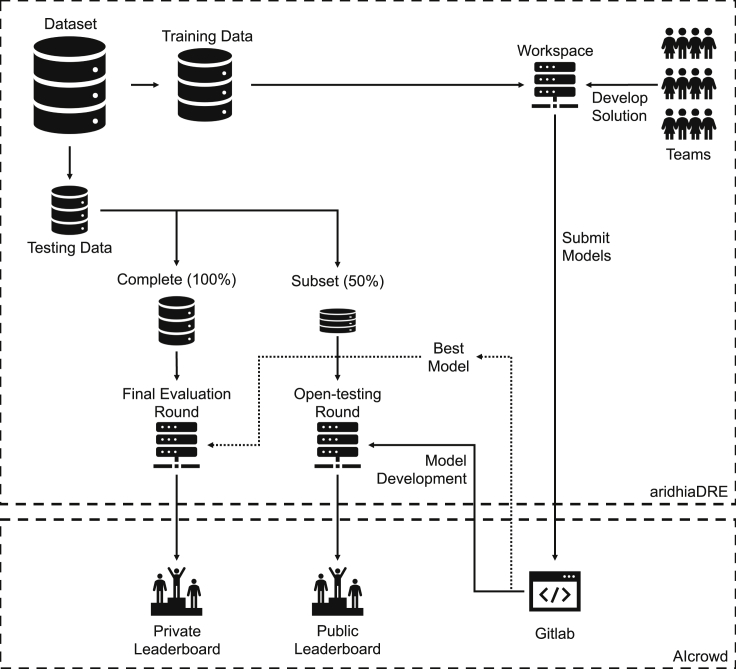
DSAI challenge setup The challenge was hosted on Aridhia DRE and AIcrowd. It consisted of an open-testing round for teams to refine and calibrate their models, and a final evaluation round.

The DSAI challenge was hosted on an Aridhia Digital Research Environment (Aridhia DRE), a cloud-based platform designed for collaborative data analytics on healthcare data (see https://www.aridhia.com/). Each team was provided a remote workspace for accessing the data, computing resources for developing their models, and a Git repository hosted by AIcrowd for managing their source code (see https://www.aicrowd.com/). AIcrowd was also used to host a leaderboard and discussion forum for teams to interact and answer questions. Figure 1 presents an illustration of the setup.

For the leaderboard challenge, teams were required to predict the probability of regulatory approval (i.e., the drug-indication development status) given phase 2 trial data and drug compound characteristics (see Table 1). This corresponds to a real-world decision-making scenario whereby a pharmaceutical company must decide whether to invest in a phase 3 program based on phase 2 results. We split the P2APP dataset chronologically, with drug-indication pairs that failed or succeeded before 2016 provided to the participants as training data, while those pairs that failed or succeeded in 2016 or later were held out as testing data for leaderboard evaluation. Table 2 shows the sample sizes of the training and testing data. Teams were encouraged to create new features in the core dataset, in addition to those provided, by linking new datasets (e.g., compound data) and through feature engineering.

The challenge spanned 5 months, from October 2019 to March 2020: 1 month for team registration and onboarding, 2 months for model development and submission, and 2 months for final evaluation. During the model development segment, teams built their models using the training data. They were able to receive real-time feedback on the performance of their models on a subset of the testing data (50%) and how it compared with other teams (“open-testing round”). This happened via a public leaderboard, which was updated with every submission. This gave participants the opportunity to refine and calibrate their algorithms. Additionally, each team's submissions were evaluated on the complete testing set (100%) in the final evaluation round. This information was not shown to participants during the competition, defining the private leaderboard to assess performance. We used the binary cross entropy log loss function as the primary scoring metric for evaluating the predicted probabilities.

We also trained a baseline model based on the algorithm described in Lo et al.^7^ using the same training data provided to the participants. To obtain the confidence interval of the performance of each model, we bootstrapped the testing set 1,000 times and evaluated the models on the same bootstrapped datasets.

As part of the final evaluation process, teams were required to upload the code used to train their models and a write-up describing their methods and results. An evaluation committee was formed from technical and domain experts to (1) validate the team's leaderboard performance and (2) assess the level of depth regarding business insights produced by the models. Along the technical dimension, each team's source code repository was examined to ensure that the results reported were robust and reproducible. The submission history of the top-performing teams was also reviewed to prevent gamification and ensure that they did not gain an unfair advantage by making frequent submissions. As discussed in the results, little evidence for overfitting or reverse engineering was observed. Technical evaluation also included understanding the innovative aspects of top solutions that were driving their performance in terms of data wrangling and adopted methodology. Domain experts then evaluated the insights and learning from such model interrogations and visualizations in terms of general, scientific program, and scientific trial insights. Since the potential business value of the findings would be to inform portfolio and risk-management decisions, the focus for the business evaluation was on the interpretability of the models, i.e., the ease of insight regarding the risk factors and key drivers of approval. This additional domain assessment was planned in anticipation of a potential discrepancy between top-performing models and actionable insights. However, in-depth domain expertise and feature insights proved to be clear differentiators of both winning solutions.

Subsequent to this evaluation, the two top-performing teams were selected to present their findings to a final committee consisting of Novartis leaders from its portfolio strategy and biostatistics divisions and its Digital Office, and MIT researchers A.W.L. and K.W.S. Other teams with innovative approaches were also invited to share their experience as part of a panel discussion with the broader Novartis community.

## Results

We received approximately 3,000 model submissions in the open-testing round of the leaderboard challenge. The teams explored a wide range of machine-learning models, ranging from traditional logistic regression, support vector machines, decision trees, and neural networks to ensemble methods such as random forests,^8^ gradient boosting machines, XGBoost,^9^ and combinations of multiple types of models.

**Figure 2:**
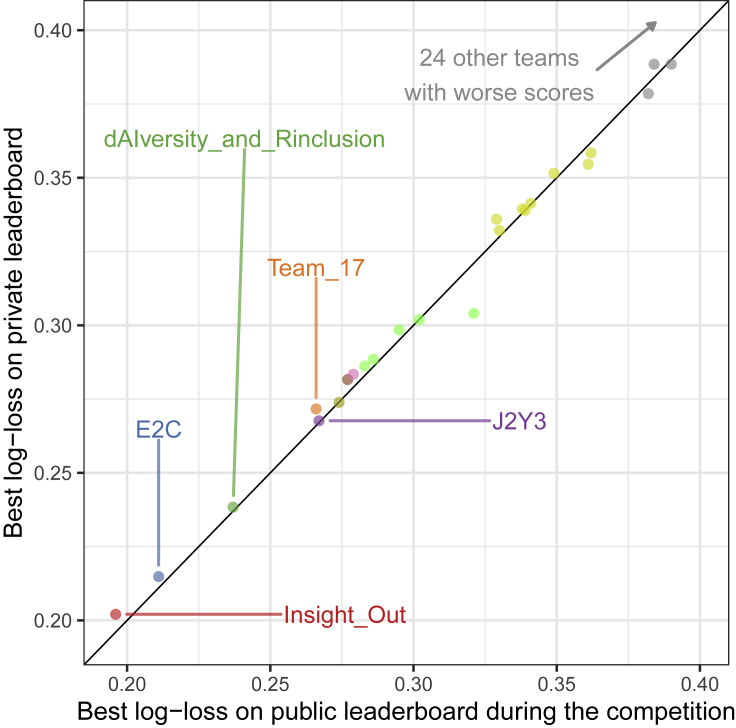
Scatterplot of public and private leaderboard scores Each point corresponds to the best-performing submission of each team. The points lay very close to the diagonal, which indicates that there is little evidence of overfitting in the competition.

Recognizing the dangers of overfitting that arise from the reuse of testing set data,^10^ we created a scatterplot of public and private leaderboard scores to assess the extent of adaptive overfitting (Figure 2). The public scores were evaluated on a subset of the testing set provided to the participants during the open-testing round, while the private scores were evaluated on the complete testing set in the final evaluation round. In the ideal case, the points would lie close to the diagonal since the public and private performance of the models would be almost identical. In contrast, deviations from the diagonal suggest possible overfitting. We observe that our scores approximated the ideal case in Figure 2, indicating that there was little evidence of DSAI challenge competitors overfitting to the public leaderboard score.

**Figure 3:**
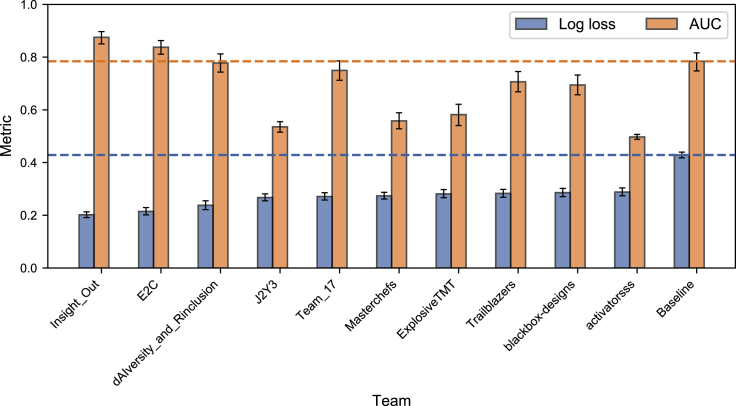
Private leaderboard log loss and AUC for the top ten ranking teams and the baseline model The error bars correspond to the 95% confidence intervals. The top two teams outperformed all other submissions in the leaderboard challenge as well as the baseline model.

In Figure 3, we compare the performance of the top ten ranking teams with the baseline model described in Lo et al.,^7^ using the private leaderboard log loss and the AUC as our metrics. While the baseline model had a worse log loss compared with the top ten best-performing teams, its AUC (0.78 with 95% confidence interval [CI] [0.75, 0.82]) was only lower than the top two teams in the challenge. This may be, in part, because the teams in the competition attempted to optimize log loss.

We focus on the approaches of the two teams that outperformed the baseline model on all metrics. These teams had different strategies and backgrounds of expertise, but were aligned in the way they harnessed human insight into their model predictions.

• The team with the top-ranked model was primarily composed of biostatisticians with significant domain expertise in clinical trial data analysis. They relied on handcrafted features that incorporated their insights into drug-development timelines and which data entries should be discarded. A team member with portfolio management experience also provided a different perspective.
• The runner-up team was primarily composed of data scientists with domain expertise in bioinformatics and cheminformatics. They relied on extensive data exploration and feature engineering, in particular developing a novel method to understand the interaction of these features, but also augmented them with clinical trial knowledge.

### Approach of the top-performing team

The top-performing model was developed by a collaborative team (team “Insight_Out”) from Novartis offices in the United States and Switzerland whose members had backgrounds in biostatistics, data science, and portfolio management. Their model achieved an AUC of 0.88 (95% CI [0.85, 0.90]), corresponding to an improvement of approximately 0.10 over the baseline model. In addition to using the core features provided in the dataset, the team created several new features to: capture information about orphan drug indications, improve the granularity of therapeutic areas; compare the relative size of phase 2 trials with the average by therapeutic area and disease; classify the drug candidate as a novel compound, a life cycle management (LCM) project, or a generic; and determine whether an international nonproprietary name (INN) has been registered for the drug.

The final model of the top-performing team was an ensemble consisting of two XGBoost models and one Bayesian logistic regression (BLR) model. See https://mc-stan.org/rstanarm/ and https://github.com/stan-dev/stancon_talks/for implementation details of the BLR model. The XGBoost models, known to be highly effective for tabular data, were trained using 263 raw and derived features, using time-series cross-validation with different levels of hyperparameter tuning (i.e., using simple heuristics and a more sophisticated approach involving differential evolution optimization).^11^ Subsequently, logistic regression with a ridge penalty was used to combine the trial-level predictions of the XGBoost models into predictions at the drug-indication level.

The BLR model was trained using case weights based on covariate balancing propensity scores,^12^ with greater weights given to cases that had a greater propensity of appearing in the test set. The BLR model allowed the team to incorporate its judgment on the likely effects of a smaller set of features. These included granular therapeutic areas as a random effect, novelty (e.g., that a drug was non-generic, and not an insulin or a flu vaccine), the relative phase 2 accrual versus the disease average, the success rates of drugs with the same mechanism of action, INN assignment, and trial outcomes, as well as interactions between these features. The parameters were estimated via Markov chain Monte Carlo sampling.

Ensembles of diverse models can generally outperform any individual model.^13^ The ensemble predictions were obtained as a weighted average of the predictions from the XGBoost and BLR models. Afterward, the predictions were post-processed using heuristics derived from the team's domain expertise. For example, the predictions for trials after 2018 were rescaled between 0.001 and 0.1 because the team believed that obtaining approval within 2 years of completing phase 2 was unlikely. These limits were determined based on prior elicitation using the roulette method.^14^ In addition, the team introduced upper and lower bounds for their predictions to reduce the impact of overconfident and overpessimistic predictions on the log loss, since extreme predictions that are incorrect are heavily penalized under the log loss metric.

The team found that the phase 2 accrual relative to the disease average was one of the strongest predictors of approval. The likelihood of success increased for programs with above-average accrual compared with other programs for the same disease. In contrast, programs with below-average accrual were more likely to fail. The team also found prior approvals for any indication (e.g., LCM programs), past approvals of other drugs for similar indications, and well-established modes of action improved the odds of approval, suggesting that repositioning an approved drug for a new indication is less challenging than developing a first-in-class new chemical entity. On the other hand, they found that drugs that targeted difficult-to-treat diseases (i.e., therapeutic areas that have historically demonstrated a much lower probability of success in clinical development versus their counterparts), such as cancer or Alzheimer's disease, were more likely to fail. Trial termination (whether due to lack of efficacy, safety issues, or pipeline reprioritization), poor patient enrollment versus planned accrual, and the absence of an INN were also strong indicators of failure. See Note S2 for further details of the methodology and findings.

### Approach of the second-place team

The second-place model was developed by a team of data scientists and researchers from the Genomics Institute of the Novartis Research Foundation (team “E2C”). This model achieved an AUC of 0.84 (95% CI [0.81, 0.86]), corresponding to an improvement of approximately 0.06 over the baseline model. The team performed extensive feature engineering, creating rank-normalized versions of features known to demonstrate temporal coupling (e.g., phase 2 trial durations, which have shown greater mean and spread over the years). This was done because decision-tree algorithms tend to be inefficient at incorporating heteroskedasticity. In addition to the core features in the dataset such as prior approval, the team created new variables to capture the impact of development history on future approvals. For example, they computed the number of past trials in which each drug had been involved by phase, by outcome, and in aggregate, regardless of indication. Additionally, they made a similar computation for indications and indication groups, aggregating them over all drugs. The team also used natural language-processing techniques, such as the TFIDF (term frequency-inverse document frequency) algorithm, to convert text data for trials into feature vectors. Because the set of features under consideration was large, the team performed stepwise feature selection using random forests to identify a parsimonious set of factors.

From the outset, the second-place team focused on the XGBoost model, an algorithm that has a strong track record in data science competitions. They explored multiple training-validation strategies for hyperparameter selection, eventually settling on the random 5-fold cross-validation approach. Like the top team, they also post-processed trial-level predictions from the XGBoost model, based on expert knowledge. For example, they reduced the predictions for trials after 2018 because team members believed that approval within 2 years was unlikely, and clipped overconfident and overpessimistic predictions to reduce the impact of outliers on the log loss scoring metric. Unlike the leading team, however, they obtained predictions for each drug-indication pair by using the maximum trial-level prediction across all trials associated with the drug-indication pair, as opposed to using penalized logistic regression. They hypothesized that the best-performing trial would dominate the outcome of the drug-indication pair regardless of any lack of evidence in other trials in support of efficacy.

Among the final set of features, the second-place team found that rank-normalized variables were generally favored over their raw, unnormalized counterparts, thus verifying the importance of normalization. Out of the top 20 most important features, eight were novel features created by the team and not provided in the core dataset (see Note S3 for further details). They found that the top features were largely consistent with those reported by Lo et al.,^7^ e.g., trial outcomes, trial accrual, prior approval, and sponsor track record. Moreover, they found that drugs with strong development histories, as quantified by the percentage of past trials with positive outcomes, were more likely to be successful. Over- and underenrollment with respect to the target accrual were also associated with lower success rates, not an entirely unexpected finding since these signs hint at poor trial operation or a lack of efficacy. Interestingly, the team found that trials with a younger age criterion for inclusion tended to be more successful. However, features created from text data did not seem to contribute meaningful predictive value.

In addition to single-feature analysis, the second-place team went a step further to identify informative feature pairs. They found strong interaction effects between trial outcomes and drug-development history, e.g., the historical success rate of past trials and the presence or absence of prior approval. For example, given a successful trial with its primary endpoints met, a drug with prior approval for other indications was almost twice as likely to be approved in comparison with a new compound without any prior approval. The team also found that drug developers with a strong track record had higher probabilities of success in indications that had been less explored in the development process, as quantified by the cumulative number of past trials.

In addition, the team observed strong coupling between the success of anti-cancer drugs and their development history. The likelihood of success of an anti-cancer drug was five times greater with a prior approval than without. This effect was less pronounced in non-cancer programs, where the ratio in success rates conditional on prior approval was only twice as great. The team hypothesized that historical success rates and prior approval were especially important for anti-cancer drugs because it is not uncommon for effective cancer therapies to work across multiple cancer subtypes (e.g., chemotherapy), and therefore an approval in one subtype was predictive of potential success in other subtypes. See Note S3 for further details of the methodology and findings.

We also evaluate the performance of both models when combined, taking simple composites of the winning teams' predictions, e.g., the maximum, minimum, arithmetic mean, and geometric mean. The combined models achieved AUCs that are close to the top-performing model, ranging between 0.86 and 0.88 depending on the composite used.

## Discussion

MIT and Novartis researchers collaborated on an in-house DSAI challenge to develop machine-learning models for predicting clinical development outcomes, building on Lo et al.,^7^ whose work used one of the largest pharmaceutical pipeline databases in the world, provided by Informa. To the best of our knowledge, this challenge represents the first crowd-sourced collaborative competition to use pharmaceutical data for this purpose, in this case updated snapshots of the same Informa databases used in the earlier MIT study. In total, over 50 cross-functional teams from 25 international Novartis offices participated in the challenge. We received approximately 3,000 model submissions over a 2-month period.

Internal data science competitions are both an opportunity for a company to address business problems and a learning opportunity for the company's data science community. From this perspective, the large number of Novartis associates who chose to actively participate in the process and had the chance to expand their data science skill set was encouraging.

The probability of success is one of several key parameters, in combination with unmet medical need and market opportunity, which clinical researchers, biopharma investors, and portfolio managers consider when making scientific and business decisions about drug development. Accurate estimates of this parameter are therefore critical for efficient risk management and resource allocation. The top-performing teams in their winning solutions delivered additional heuristics with respect to predicting the probability of success:

• Identification of novel features predictive of probability of success (as outlined above)
• Novel approaches and methodologies for feature extraction, combining domain expertise and machine learning
• Creative ways of introducing additional data types to the problem, such as unstructured text and biochemical data—for example, several teams presented ways of connecting new data types, although this in itself did not translate into top leaderboard performance

Additionally, the discussion about the availability of specific information at the time of decision making about the fate of a project was helpful for assessing the potential for target leakage in the solutions of external vendors offering similar predictive solutions.

However, the DSAI challenge also had several limitations. First, the P2APP dataset was split chronologically, using drug-indication pairs that failed or succeeded before 2016 as training data, and those that failed or succeeded in 2016 or later were held out as testing data. Due to the nature of drug development, however, some boundary effects were inevitably present in the last years of the testing data. Because drugs tend to fail much more quickly than those that are approved, the majority of the trials completed after 2018 ended in failure. (The probability of phase 2 to approval in the testing data is 9.3% for all trials in aggregate but only 1.8% for trials completed after 2018.) With their experience and expertise in drug development, both teams eventually discovered this artifact in the data, and were able to improve their model performance by adjusting their predictions for trials after 2018. While such adjustments were useful in the competition, they add little practical value for real-life application.

Second, some available features reflected a decision already taken by a company to terminate a project. These included trials that were stopped due to pipeline reprioritization, a small-sized phase 2 program due to stopping the program after an initial small trial, and the failure to apply for an INN. Not all such information is available at the time of decision making in practice. These limitations illustrate that in order to make data science competitions directly useful for business problems without substantial modification, it is important to align extremely closely the prediction task in the competition with the real-world business problem

We also received feedback from knowledgeable participants that the core dataset lacked key information that decision-makers typically take into consideration, such as the preclinical data, detailed safety and efficacy data, and biological plausibility of the mechanism of action. Unfortunately, investigators do not usually release this information to the public domain for strategic reasons. It is therefore unsurprising that such data are not available in commercial pharmaceutical databases based on publicly available sources of information. Potentially, this limitation could be overcome by recent progress in deep-learning approaches to natural language processing, which may enable information about trial protocols, development programs, and drugs to be extracted from unstructured text data sources.

### Conclusion

By tapping the power of crowd-sourcing and the domain expertise of Novartis researchers working in cross-disciplinary teams, we have shown the potential for DSAI challenges to generate predictive models for drug-development outcomes that outperform existing models from the academic literature. In addition to validating features previously associated with drug approval in the MIT study, the DSAI challenge has provided new insights into the drivers of drug approval and failure. Ultimately, these new predictive models can be used to augment human judgment to make more informed decisions in portfolio risk management. Nevertheless, there remains a clear opportunity to further improve the models in this competition. We believe that more accurate models can be developed with access to better quality and more comprehensive data, and a broader pool of challenge participants.

## Experimental procedures

### Resource availability

#### Lead contact

Andrew W. Lo, MIT Sloan School of Management, 100 Main Street, E62–618, Cambridge, MA 02142. (617) 253-0920 (tel), (781) 891-9783 (fax), alo-admin@mit.edu (email).

#### Materials availability

This study did not generate new unique reagents.

#### Data and code availability

The source code of the algorithms is available at https://github.com/bjoernholzhauer/DSAI-Competition-2019 (Insight_Out) and https://github.com/data2code/DSAI-Competition-2019 (E2C). The data supporting the current study have not been deposited in a public repository, due to their proprietary nature. The data are available from Informa at https://pharmaintelligence.informa.com/products-and-services under Pharmaprojects and Trialtrove. Restrictions apply to the availability of the data, which were used under license for this study.

## Supplemental information

Document S1. Supplemental experimental procedures, Notes S1–S3, Figures S1–S24, and Tables S1, S2, and S4

Table S3. Definitions of the trial-level features used in Xgboost models

Document S2. Article plus supplemental information
