# Supplementary material for: Predicting drug approvals: The Novartis data science and artificial intelligence challenge
Source: Patterns (N Y). 2021 Jul 21;2(8):100312. doi: 10.1016/j.patter.2021.100312 (PMC8369231; doi:10.1016/j.patter.2021.100312)
Supplement: Document S1. Supplemental experimental procedures, Notes S1–S3, Figures S1–S24, and Tables S1, S2, and S4 [file mmc1.pdf]

Patterns, Volume *2*

Supplemental information

Predicting drug approvals: The Novartis data science and artificial intelligence challenge

Kien Wei Siah, Nicholas W. Kelley, Steffen Ballerstedt, Björn Holzhauer, Tianmeng Lyu, David Mettler, Sophie Sun, Simon Wandel, Yang Zhong, Bin Zhou, Shifeng Pan, Yingyao Zhou, and Andrew W. Lo

Supplemental Experimental Procedures

Supplemental Note S1: Core Dataset

We constructed our datasets using two Informa® databases: *Pharmaprojects* and *Trialtrove*, two separate relational databases organized by largely different ontologies. We extracted drug-specific features and drug–indication development status from *Pharmaprojects*, and clinical trial features from *Trialtrove*.

First, we identified all drug–indication pairs with known outcomes in *Pharmaprojects*. Next, we dropped pairs that did not have any trials captured in *Trialtrove*. (We note that the disease coverage in *Pharmaprojects* and *Trialtrove* is slightly different.) Because missingness is present in both *Pharmaprojects* and *Trialtrove*, we imposed several additional filters to make sure that all samples collected were usable for analysis.

We summarize the steps in Table S1 and Figure S1. It is important to note that the drug, indication, and trial relationships in the databases are surjective and non-injective: different drugs may target the same indication, and some trials may involve multiple drug–indication pairs. This is to be expected since one drug can be indicated for multiple diseases, a disease can have more than one treatment, and it is not uncommon for a trial to involve two or more related primary investigational drugs. In Figure S2, we plot the probability of phase 2 to approval over time in the dataset.

We extracted drug compound attributes and clinical trial characteristics from *Pharmaprojects* and *Trialtrove*, respectively (see Table S2). In addition to structured features readily available in the databases, we created an augmented set of variables that captured sponsor track record and investigator experience. To quantify a particular trial sponsor's track record in successfully developing other drugs, we used the number of prior approved and failed drug–indication developments; and for past trials for phases 1, 2, and 3 separately, we used the total number of trials sponsored, the number of trials sponsored with positive and negative results, and the number of trials sponsored to completion and termination. We used the end date of the last trial of the drug–indication pair under consideration as the cutoff for considering prior experience since the last end date will be the time of prediction. We abstracted investigator experience in the same manner.

Lastly, we also constructed a binary drug–indication pair feature that indicates whether a drug has previously been approved for another indication. Similarly, we used the end date of the last trial as the cutoff for considering prior approval.

Table S1. Filters for constructing P2APP.

	Rationale
**Drug–indication Pairs in *Pharmaprojects***	
Trials observed in *Trialtrove*	We excluded pairs for which we do not observe any trials in *Trialtrove*.
Known approval date (if approved)	We defined the approval date as the earliest date a drug–indication pair was approved in any market. We require these dates to perform time-series analysis.
Known failure date (if failed)	We defined the failure date as one year after the end-date of the last phase 2 or phase 3 trial (if any), whichever is latest.
**Clinical Trials in *Trialtrove***	
Phase 2 trials	P2APP focuses on phase 2 trial data
Known end date	We required these dates to create sponsor track record and investigator experience, and to perform time-series analysis.
Known sponsors and disease types	Trials not tagged with sponsor/disease types are typically out of *Trialtrove* commercial coverage and not maintained.

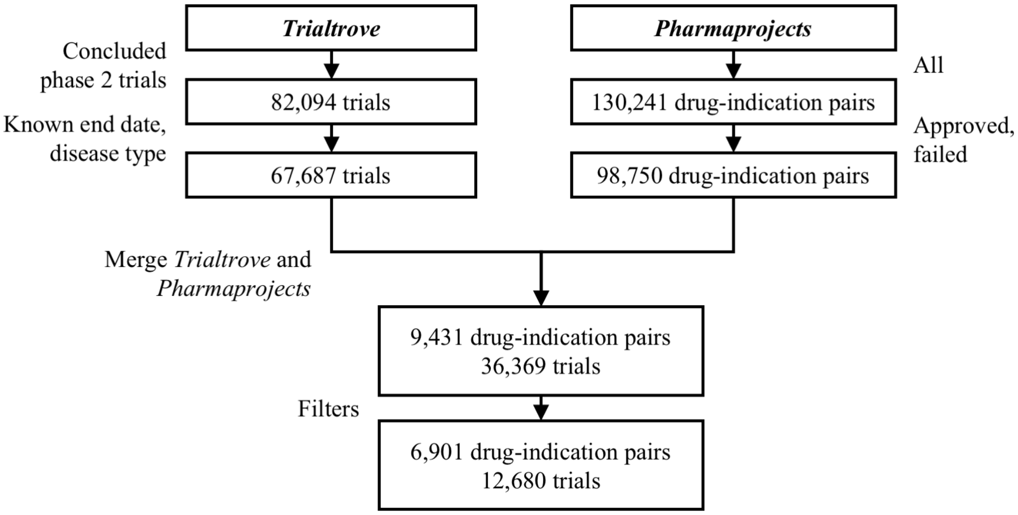

Figure S1. Sample sizes at each step of data pre-processing.

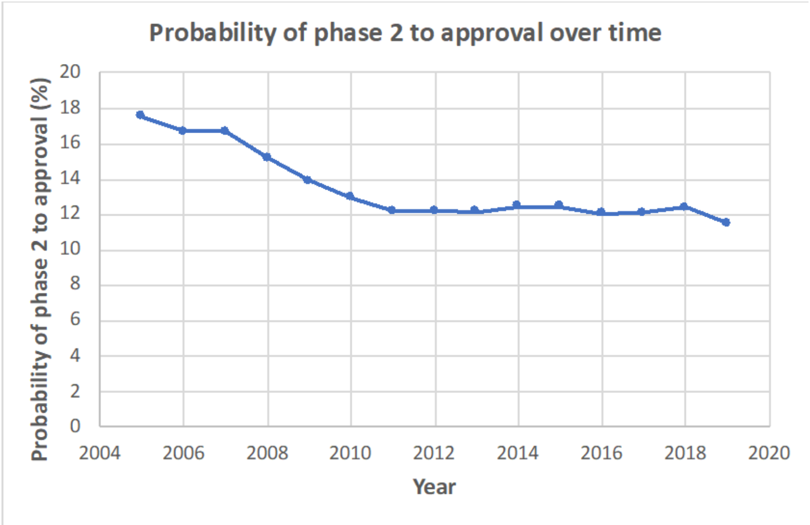

Figure S2. Probability of phase 2 to approval in the dataset plotted using expanding windows.

Table S2. Features extracted from *Pharmaprojects* and *Trialtrove*. We define multi-class features as categorical features that have mutually exclusive categories, i.e., features can belong to only one category at any time; and multi-label features as features that have non-mutually-exclusive categories, i.e., features can belong to more than one category simultaneously.

	Examples	Type
**Drug–indication Pair**		
Biological target	Cytokine/Growth factor; Enzyme; Ion channel; Receptor; Transporter	Multi-label
Country	China; India; Japan; United States	Multi-label
Drug–indication development status	Approved; Failed	Binary
Indication	Cancer, lung, small cell; Cancer, lung, non-small cell; Cancer, brain	Multi-class
Mechanism of action	Cell cycle inhibitor; DNA inhibitor; Ion channel antagonist; Protein kinase inhibitor	Multi-label
Medium	Capsule, hard; Capsule, soft; Powder; Solution; Suspension; Tablet	Multi-label
Name	Free text	String
Origin	Biological, protein, antibody; Biological, protein, recombinant; Chemical, synthetic	Multi-label
Prior approval of drug for another indication	True; false	Binary
Route	Inhaled; Injectable; Oral; Topical	Multi-label
Therapeutic class	Anti-viral, anti-HIV; Anti-cancer, immunological; Anti-epileptic	Multi-label
**Trial**		
Attribute	Biomarker/Efficacy; Biomarker/Toxicity; Pharmacogenomic - Patient Preselection/Stratification	Multi-label
Actual accrual	Integer	Numerical
Disease type	Bladder; colorectal; ovarian	Multi-label
Duration	Integer	Numerical
Exclusion criteria	Free text	String
Gender	Male, female, both	Multi-class
Investigator experience	Refer to sponsor track record	Numerical
Location	Canada; Europe; United Kingdom; United States	Multi-label
Number of identified sites	Integer	Numerical
Outcome	Completed, Negative outcome/primary endpoint(s) not met; Completed, Outcome indeterminate; Completed, Positive outcome/primary endpoint(s) met; Terminated, Safety/adverse effects	Multi-label
Patient age	Integer	Numerical
Patient population	Free text	String
Patient segment	Stage I; stage III; stage IV; second line; pediatric	Multi-label
Phase 2 end date	Date	Date
Primary endpoint	Free text	String
Sponsor	Duke University Medical Center; National Institutes of Health; Celgene	Multi-label
Sponsor track record	Number of prior approved drug–indication pairs; Number of prior failed pairs; Total number of phase 1 trials sponsored; Number of phase 1 trials with positive results; Number of phase 1 trials with negative results; Number of completed phase 1 trials; Number of terminated phase 1 trials; Total number of phase 2 trials sponsored; Number of phase 2 trials with positive results; Number of phase 2 trials with negative results; Number of completed phase 2 trials; Number of terminated phase 2 trials; Total number of phase 3 trials sponsored; Number of phase 3 trials with positive results; Number of phase 3 trials with negative results; Number of completed phase 3 trials; Number of terminated phase 3 trials	Numerical
Sponsor type	Academic; Industry, all other pharma; Industry, Top 20 Pharma	Multi-label
Status	Completed; terminated	Binary
Design	Free text	String
Design keywords	Cross over; Double blind/blinded; Efficacy; Multiple arm; Open label; Pharmacodynamics; Pharmacokinetics; Placebo control; Randomized; Single arm	Multi-label
Target accrual	Integer	Numerical
Therapeutic area	Autoimmune/Inflammation; Cardiovascular; CNS; Infectious Disease	Multi-label

Supplemental Note S2: Detailed Overview of Top-Performing Team

High-level Overview

A high-level overview of the solution by the team in top performing place is shown as a flowchart in Figure S3. The solution uses an ensemble of three different predictions followed by some post-processing. (Source code available at https://github.com/bjoernholzhauer/DSAI-Competition-2019.)

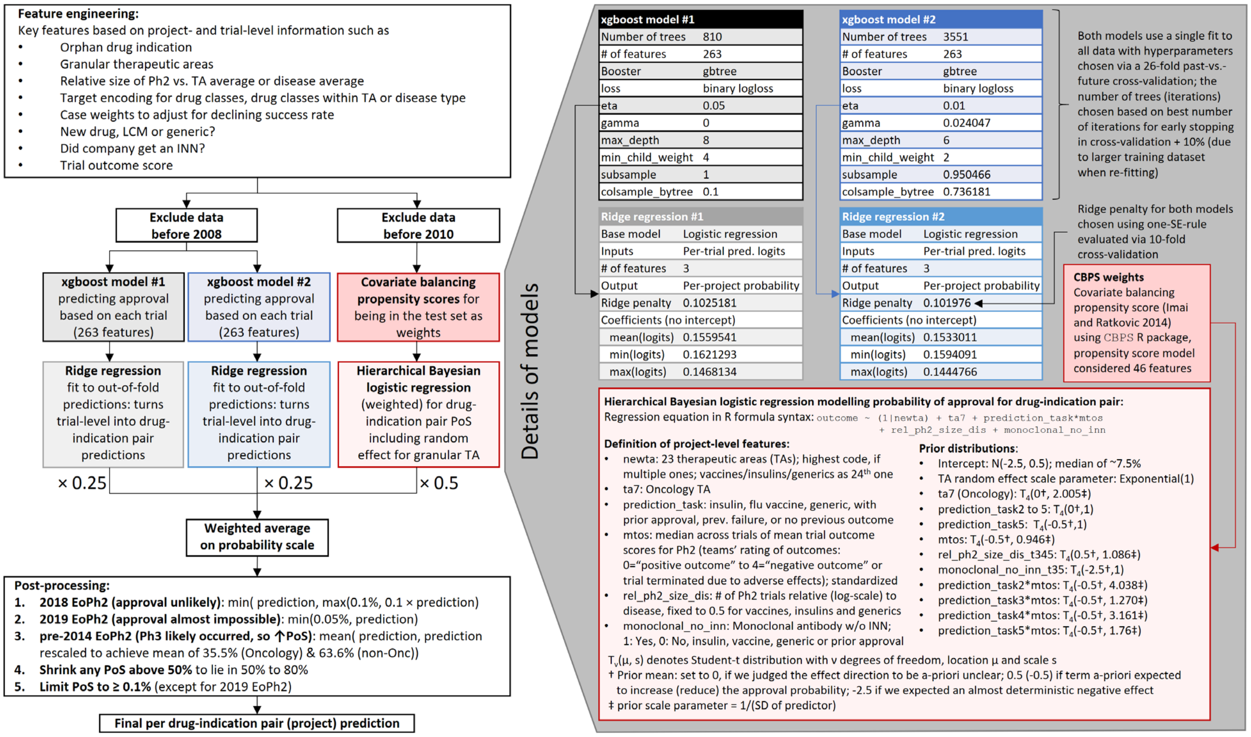

Figure S3. High-level flowchart of top performing team.

Extreme gradient boosting (XGBoost) was found to perform very well, as to be expected on tabular data.[1] Therefore the final solution included two XGBoost models that were fit on trial-level data. To obtain a single prediction per drug–indication pair, the predictions for each phase 2 trial for the drug–indication pair were summarized using 3 features (the mean, minimum, and maximum of the predicted probabilities on the logit-scale) and combined into a single prediction using a ridge regression model. The ridge regression models were fit to the predictions of the validation-fold of each of the 26 cross-validation splits (out-of-fold predictions). Hyperparameters for the ridge regression model were chosen based on the one-standard-error rule via a 10-fold cross-validation. The ridge regression model had the desirable additional effect of improving the calibration of predicted probabilities, which we expected to improve the binary log-loss on the unseen test set data.

In contrast, the third model in the ensemble was a hierarchical Bayesian logistic regression (BLR) model fit on the drug–indication level using features summarized across the trials for the drug–indication pair. The `rstanarm` R package (see https://mc-stan.org/rstanarm/) that uses the Stan modelling language and Markov Chain Monte Carlo sampler (https://mc-stan.org) in the background were used to fit the model.

For the Bayesian model, somewhat informative (i.e., expressing considerable uncertainty about the prior judgements) prior distributions were chosen based on judgments on the likely effects of different features. While this model performed slightly worse than the XGBoost models as assessed by cross-validation (CV), it substantially improved the performance of our final model ensemble.

The observations used in fitting the BLR model were given weights based on covariate balancing propensity scores (CBPS) for being in the leaderboard test set. This had the motivation that there appear to be notable differences in the predictors of the training data and predictors of the leaderboard test set. While we did not know the outcomes to be predicted for the test samples and how exactly these might be influenced by this distribution shift in predictors, we assumed that it would be important to address this. CBPS weights are one of the possible techniques for addressing such a distribution shift.[2]

There is a notable decline in approval rates over time in the training data. Some of this trend may be due to an increasing difficulty in obtaining approval for a new drug. However, we were concerned that this might be the result of failed projects missing from the database for the years before trials had to be registered on clinicaltrials.gov in 2007. We tested this theory by discarding some of the training data both via cross-validation and in terms of leaderboard score, and obtained improved results. Thus, we did not use data before 2008 in our XGBoost models and, in the case of the BLR, even discarded data from 2008 and 2009.

Hyperparameter Tuning for XGBoost

Hyperparameter tuning was based on a 26-fold past-vs.-future CV scheme.

- For the first XGBoost model it was done manually using the rule of thumb that one should tune hyperparameters separately using relatively high learning rate (eta=0.1) in the following order: 1) max_depth, 2) subsample, 3) min_child_weight, 4) colsample_bytree.
- For the second XGBoost model, hyperparameters were tuned at a high learning rate using the differential evolution algorithm[3] implemented in the `DEoptim` R package for global optimization of the leader-board metric as assessed by CV.

For both XGBoost models the final learning rate was 0.05 and the number of trees (learning rounds) for refitting with the whole data was identified using the CV-log-loss (with the optimal number of trees increased by 10% for refitting on the whole dataset to account for the larger dataset).

The selected final hyperparameters are shown in Figure S3.

In contrast to the XGBoost models, the modelling decisions for the Bayesian logistic regression were taken based on the team judgments, because this resulted in substantially better public leaderboard performance than attempts to automatically tune the hyperparameters of prior distributions.

Cross-validation

An appropriate validation set-up is critical for making modelling choices in a principled way that avoids overfitting[4] – i.e., for arriving at a solution that generalizes well to the unseen leaderboard outcomes. The CV approach for hyperparameter tuning followed known general principles for CV (see also https://www.fast.ai/2017/11/13/validation-sets/ accessed 6 April 2021) by

1) attempting to approximate the actual prediction task as closely as possible by splitting the time series of data by past-versus-future (while still offering multiple splits that are as independent as possible for evaluation) and
2) ensuring that studies from the same drug–indication pair are never in the training data and the validation data of a split.

We mixed several possible ways of doing past-vs.-future splits in the 26 different folds we created. These ideas included:

- overlapping past-vs.-future splits (e.g., <2012 vs. >= 2012, <2013 vs. >= 2013, etc.),

- splitting the same future into several bits (e.g., fold 1 = 1/5 of >= 2012, folds 2 = the next 1/5 of >= 2012, etc.), and
- variants where you either try to emulate predicting for the same drug and/or predicting for previously unseen drugs (one could look at those two tasks separately).

As to be expected, random splits correlated less with the public leaderboard score than splitting past-vs.-future.

For the ridge regression, a 10-fold CV scheme on the out-of-fold predictions was used. This CV scheme always grouped all records for a drug–indication pair for any of the 26-validation folds into the same fold.

Feature Engineering

A wide range of additional features (or predictors) was derived in addition to those already provided by the competition organizers. The definition of each of the 263 features used by two XGBoost models is summarized in Table S3. How these features were summarized into features at the drug–indication pair level for the BLR model is summarized in Figure S3.

We highlight a few key features that had high variable importance in the XGBoost models and had somewhat more complex derivations.

One set of key features captured the relative size (on the log-scale) of the phase 2 program (rel_log_size_dis: rel_ph2_size_ta) for the drug–indication pair relative to other drug–indication pairs within the same therapeutic area and for the same disease type. Size was either in terms of the patient number or in terms of the number of phase 2 trials.

Prior approvals for a drug, but also for other similar drugs that share a mode of action is clearly a potentially useful feature. However, for the latter, important information is lost if we focus on just the proportion of prior approvals for similar drugs: 1 approval for one drug is not the same as 20 approvals for 20 drugs—in the latter case our confidence about a mode of action should be increased compared to the first scenario. Similarly, one failure with a previous drug may not be very informative, but repeated failures of many drugs would be. To capture this information, we used two approaches:

1) **Approval counts:** The simpler of the two approaches was to count the number of approvals for a mode of action up to and including the year of the phase 2 end of a drug–indication pair.
2) **Target encoding:** We used a form of target encoding[5,6] adapted to the time series nature of the data by creating a target encoding based only on data up to and including the year of the phase 2 end of a drug–indication pair.

We did this for any approval, approvals in the same therapeutic area and for the same disease type. A drug may have multiple modes of action, in which case we averaged the target encodings for the different modes of action. The derived variable is “prior_approval” in Table S3. A more sophisticated way of dealing with different modes of action could be a direction for future research.

While only limited information on the outcomes of phase trials was available, we created a trial end score to summarize this information (termreason:trailendscore4). Within each trial, one feature was the mean of these scores, while another feature was the worst score.

Other important features included whether a drug is a monoclonal antibody (is_mab), a generic (is_a_generic), an insulin (insulin), or a flu vaccine (fluvacc), and whether an international non-proprietary name (INN) (unwilling_to_pay_12k) is on the dataset for a drug (based on regular expression to distinguish brand names and/or company internal project codes from official INNs).

Table S3. Definitions of the trial-level features used in XGBoost models.

[See Excel file.]

Fitted Models

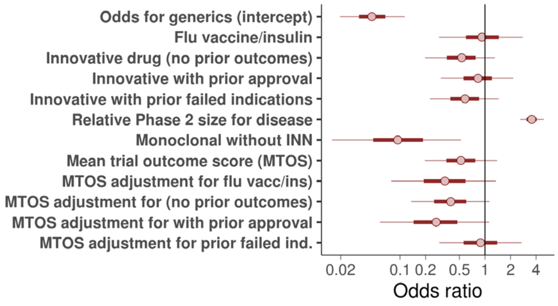

Figure S4. Odds ratios for non-disease area variables from the Bayesian logistic regression used in the final submission.

Figure S4 and Figure S5 show the marginal posterior odds-ratios for each feature obtained from the BLR model. These figures are split according to whether model terms are fixed effects (Figure S4) or random disease area effects (Figure S5). The shown point estimates are posterior medians, the inner intervals are 50% credible intervals, and the outer intervals are 95% credible intervals.

Firstly, a phase 2 program with more trials compared with other drugs for the same disease is associated with a higher probability of approval. This may partially reflect that larger phase 2 programs could result in better decisions about phase 3, but to a considerable extent reflects that projects that fail early in phase 2 will tend to have fewer phase 2 trials than projects that succeed in initial trials. That is, this variable may reflect a decision by companies to terminate a project.

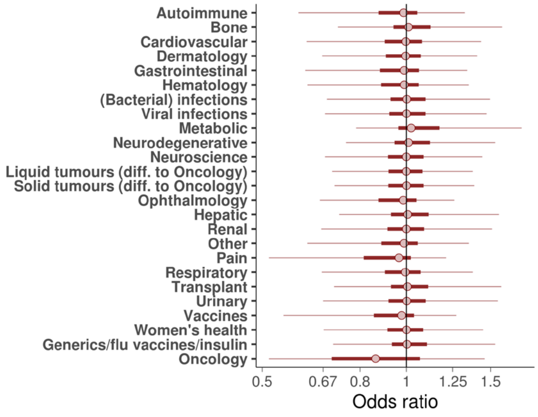

Figure S5. Odds ratios for disease area variables from the Bayesian logistic regression used in the final submission.

Prior approvals for other indications increase the probability of approval. This is logical: A prior approval shows that a drug has at least some clinical effect and makes unexpected safety findings less likely.

If there is a well-understood mode of action and regulatory pathway—such as, for flu vaccines and insulins—the probably of approval is higher, too.

On the negative side, innovative drugs have a lower probability of success than generics. This reflects that the uncertainty about them is greater.

As would be expected, the mean trial outcome score (MTOS) we created is a major predictor of success: projects with studies stopped due to pipeline re-prioritization, strategy shift, safety issues, or lack of efficacy rarely lead to an approval. Similarly, when a company does not obtain an INN for a drug, it does not intend to start a phase 3.

We also note that oncology projects tend to fail more often than drugs in other therapeutic areas.

Three different feature importance metrics for the two XGBoost models are shown in Table S4 for the top-25 predictors in the XGBoost models. As can be seen, no single feature dominates the feature importance, but several features that capture the relative size of the phase 2 program, the target encodings for modes of action, and the trial outcomes scores dominate this list. The lack of a single feature having much higher importance is partially due to the high correlation between the large number of related features.

Table S4. Feature importance for top 25 predictors in XGBoost models.

Feature	XGBoost model #1			XGBoost model #2		
	Gain	Cover	Freq.	Gain	Cover	Freq.
rel_ph2_size_ta: Relative size (number of trials) of phase 2 for TA	0.095	0.056	0.046	0.221	0.118	0.075
rel_ph2_size_dis: Relative size (number of trials) of phase 2 for disease	0.090	0.053	0.048	0.078	0.062	0.061
meanclu50: Avg. target encoding for MoAs of drug (UCrL of 50% CrI)	0.065	0.052	0.051	0.046	0.049	0.055
class_approvals: Total # of MoAs of drug with previous approvals	0.044	0.042	0.033	0.043	0.063	0.047
meancll50: Avg. target encoding for MoAs of drug (LCrL of 50% CrI)	0.031	0.032	0.035	0.049	0.055	0.062
dtmeancll50: Avg. targ. enc. for MoAs of drug for disease type (LCrL of 50% CrI)	0.052	0.033	0.039	0.066	0.036	0.038
dtmeanclu50: Avg. targ. enc. for MoAs of drug for disease type (UCrL of 50% CrI)	0.059	0.037	0.040	0.049	0.038	0.033
tameancll50: Avg. target encoding for MoAs of drug for TA (LCrL of 50% CrI)	0.062	0.041	0.048	0.022	0.024	0.036
tameanclu50: Avg. target encoding for MoAs of drug for TA (UCrL of 50% CrI)	0.034	0.037	0.038	0.028	0.036	0.045
class_counts: # of prev. approvals with MoAs of drug	0.026	0.028	0.036	0.023	0.030	0.040
rel_log_size_ta: Relative size (number of patients) of phase 2 for TA	0.021	0.027	0.040	0.024	0.022	0.047
rel_log_size_dis: Relative size (number of patients) of phase 2 for disease type	0.022	0.034	0.039	0.016	0.030	0.033
intduration: phase 2 duration	0.014	0.028	0.031	0.017	0.029	0.042
taclass_counts: Total # of approvals for MoAs of drug for TA	0.026	0.026	0.037	0.015	0.014	0.028
dtclass_counts: # of prev. approvals with MoAs of drug for disease type	0.024	0.025	0.029	0.019	0.023	0.026
time_since_first_outcome: Years since first approval or failure for the drug?	0.019	0.023	0.016	0.024	0.030	0.022
pct_accrual: Proportion of target accrual that was actually enrolled in trial?	0.011	0.021	0.030	0.009	0.015	0.029
intactualaccrual: Number of patients actually enrolled in trial	0.010	0.022	0.027	0.010	0.018	0.025
inttargetaccrual: Number of patients planned to be enrolled in trial	0.010	0.019	0.028	0.012	0.015	0.024
phaseendyear: phase 2 end year	0.013	0.015	0.021	0.019	0.015	0.023
termreason: Worst out of ranking of trial termination reasons	0.009	0.011	0.009	0.025	0.042	0.013
unwilling_to_pay_12k: Does the drug have an INN?	0.017	0.016	0.005	0.021	0.033	0.008
taclass_approvals: Total # of MoAs of drug with previous approvals in TA	0.015	0.018	0.024	0.008	0.005	0.012
dtclass_approvals: Total # of MoAs of drug with prev. approvals for disease	0.014	0.013	0.015	0.006	0.005	0.008
mean_trialendscore: Mean score for trial outcome reasons	0.014	0.013	0.010	0.007	0.012	0.004

Post-processing

The post-processing described below was the part of the overall solution that had the largest impact on the leaderboard score. We failed to obtain a satisfactory correlation of model performance estimated via cross-validation and the leaderboard scores until we introduced the post-processing of predictions for projects with an end of phase 2 in 2018 and 2019.

The post-processing reflects that the database provided to competitors was a snapshot taken in mid-2019. For phase 2 studies that ended at some point in 2019, the team considered it highly unlikely that the drug could be approved by mid-2019, because of regulatory approval timelines. Thus, we limited the predicted probability for those studies to be, at most, 0.05%.

For an end of phase 2 in 2018, an approval by mid-2019 is at least possible, but would likely only occur for rare cases that justify approval based on phase 2 data. Thus, we scaled predicted probabilities to lie between 0.1% and 10%. These limits were based on using the discrete mixture distribution of the prior distributions elicited from 4 of the 5 team members. Individual judgments were elicited using the roulette method[7] as shown in Figure S6. A decision analysis using the mixture distribution was then conducted using the log-loss metric used in the competition as the utility function.

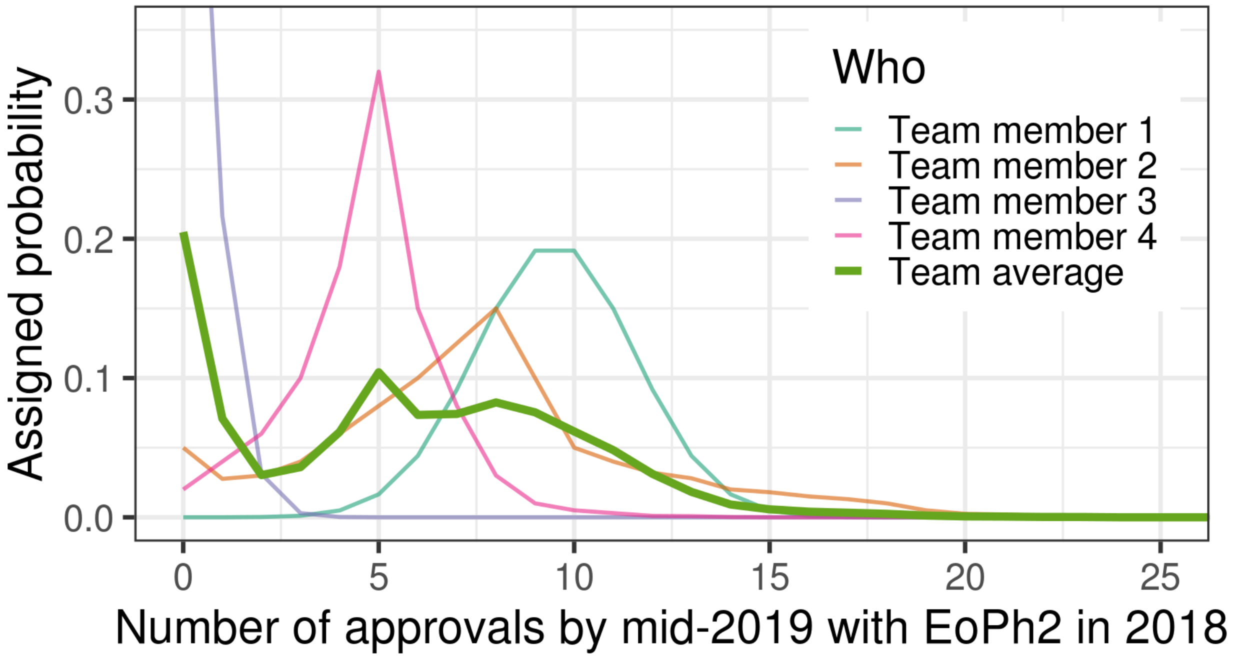

Figure S6. Elicited team prior opinions on the number of approvals by mid-2019 with end-of-phase 2 year 2018 (N=438) conditional on the outcome being known by mid-2019.

From the information communicated on how data were split into training data vs. data for the leaderboard, it seemed that a project could only have an end-of-phase 2 before 2014 and part of the leaderboard data, if a phase 3 program was initiated. For such projects we scaled the probability of approval up to be in line with phase 3 success rates.

We also avoided overly confident predictions. Given the limited available information, it appeared questionable whether predicted probabilities of approval should be above 80%, so we scaled high predicted probabilities downwards.

Additionally, we avoided (except for cases of end-of-phase 2 in 2019) predicted probabilities below 0.1%, because the binary-log-loss competition metric only rewards being right about such predictions to a very limited extent, but severely penalizes being wrong about them.

Ensemble

Figure S7 illustrates why an ensemble of the BLR with the two XGBoost models was effective. While the BLR performed slightly less well in CV and on the public leaderboard, the performance difference was relatively small and the test set predictions of the two model classes had a quite low correlation of about 0.5, which will generally help the performance of an ensemble.[4]

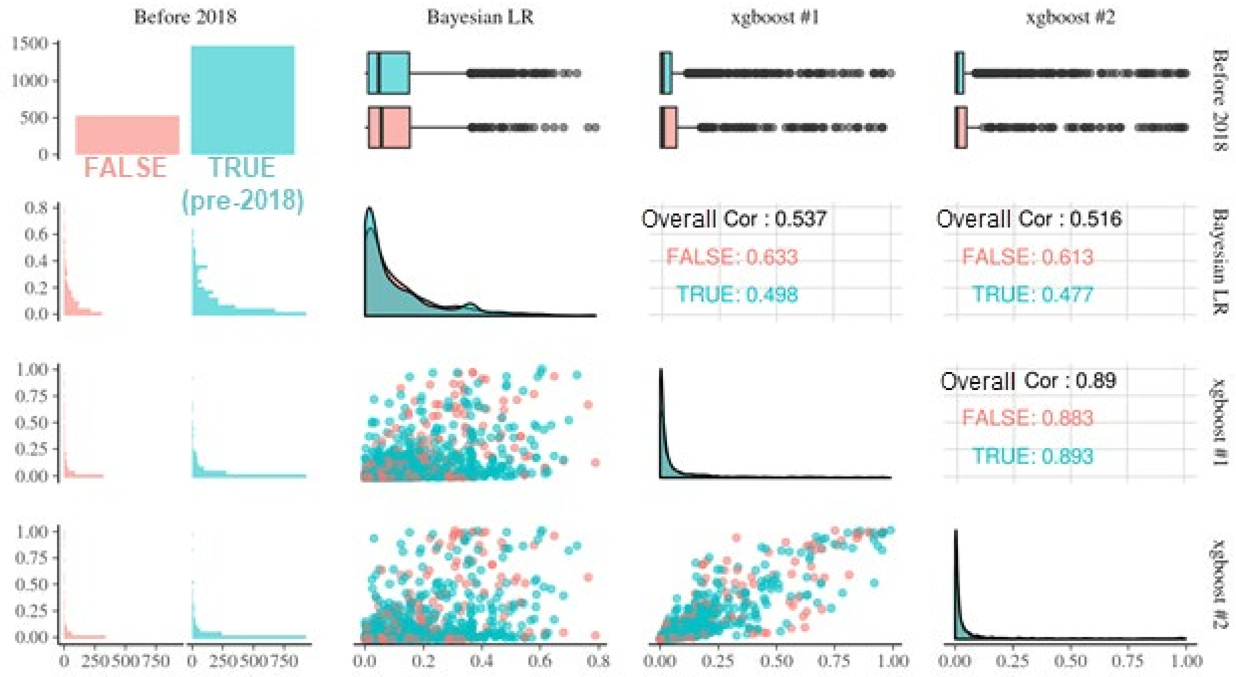

Figure S7. Correlation and distribution of predicted probabilities per drug–indication pair on the test set (overall and for pre-2018 vs. 2018 onwards).

Supplemental Note S3: Detailed Overview of Second Place Team

Overview

The overview of the E2C model development workflow is outlined in Figure S8. The team spent the first week in exploratory data analysis, focusing on visualizing the data and understanding the meaning of each given feature. This stage was critical for discovering artifacts in the data, as well as generating ideas for feature engineering. In the second week, the team carried out extensive feature engineering, explored two training-validation splitting strategies, and constructed the first decent-performing model. In the third week, the team focused on hyperparameter tuning and then further improved the model with two post-processing ideas: (1) bias correction for 2018–2019 samples; and (2) test-time augmentation across trials within the same drug–indication group. The remaining time was mostly invested in gaining insights from the model. We will highlight the key learnings from each step of the workflow. (Source code available at https://github.com/data2code/DSAI-Competition-2019.)

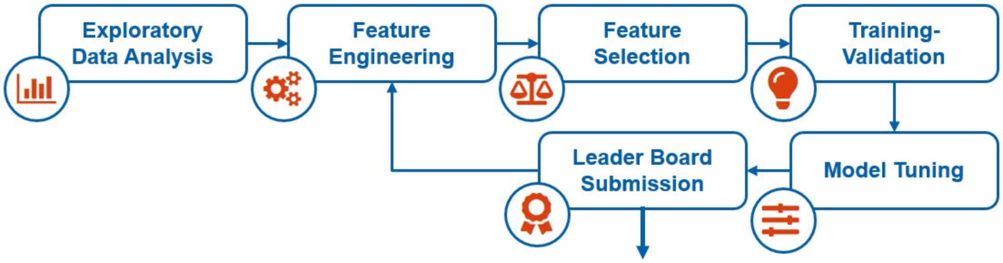

Figure S8. Overview of the E2C model development workflow.

Exploratory Data Analysis

Machine Learning Framework

We locked on using the XGBoost machine learning method early on, as it had been most frequently adopted by winning teams in Kaggle competitions on similar structured datasets. Deep learning framework was excluded, because the probability-of-success training dataset size was likely too small to train a high-performance neural network model without overfitting.

Time-decoupling

Many features were known to demonstrate strong coupling with time, as described in previous studies (e.g., Lo et al. 2019). We were able to validate those, e.g., the phase 2 trials tend to run longer and longer over the years (Figure S9).

XGBoost utilizes a decision tree structure, which is inefficient to capture such a coupling effect. Pretending trials within the top quantile of each year have a higher probability of success, as this upper quantile threshold drifts over the years. XGBoost would need multiple tree levels to handle the different thresholds across years (Figure S10). Therefore, to facilitate model training, we decided to remove the coupling between features and year through a normalization process. Normalization produced two additional features based on each raw input feature. Take feature "*abc*" as an example: (1) "*abc*_norm_by_year" was the normalized version using the mean and standard deviation of all feature values within that particular

year; (2) “*abc*_rank_norm_by_year” was its non-parametric quantile counterpart within the year, which was not sensitive to the underlying distribution.

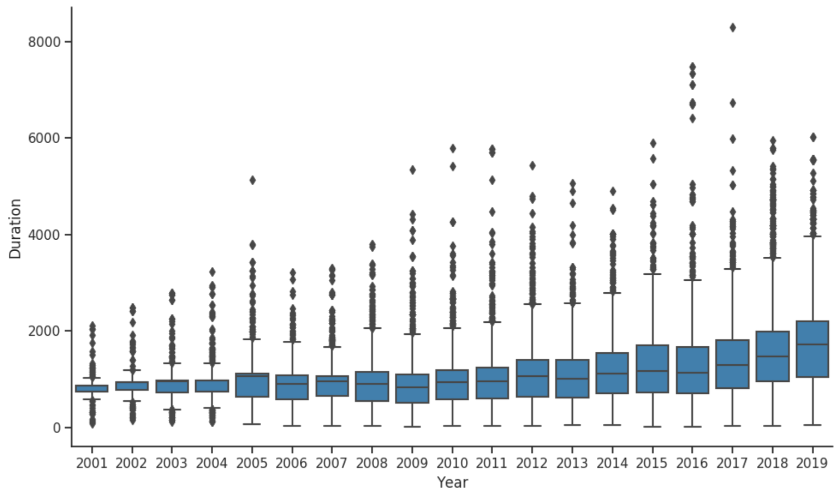

Figure S9. Phase 2 trial duration vs year.

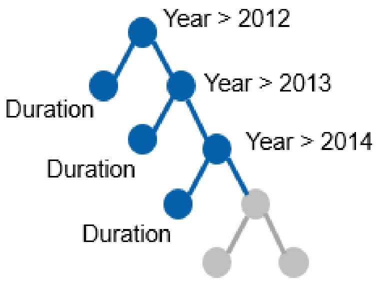

Figure S10. Modeling of the coupling between trial duration and year using a tree model would have required many tree levels.

Figure S11 shows the interaction between trial duration and year was largely decoupled after normalization. The new normalized features were expected to ease the model training process.

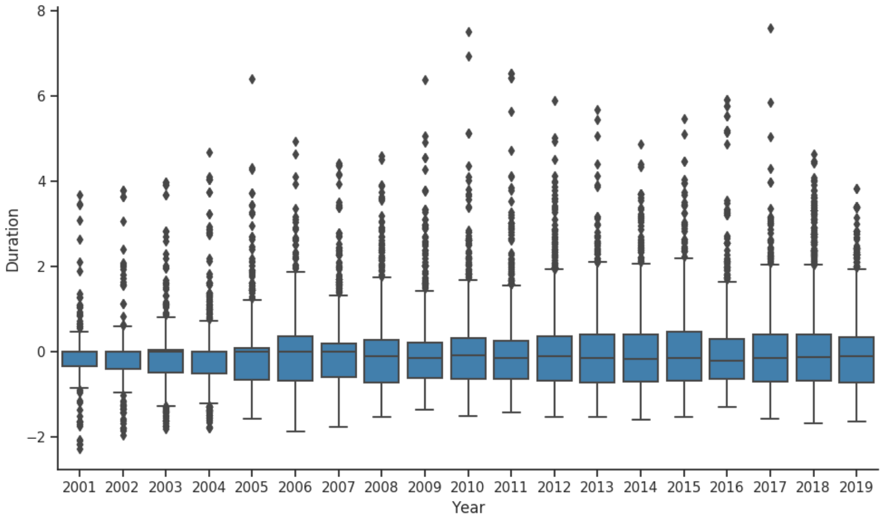

Figure S11. The time-effect on trial duration was minimized (compared to Figure S9) after mean-stdev normalization.

Normalization also tripled the number of feature counts. To avoid feature redundancy, only one of these three correlated feature versions ("*abc"*, "*abc*_norm_by_year", and "*abc*_rank_norm_by_year") was retained based on a feature ranking algorithm described later. Among the final list of retained features, normalized versions were generally favored over their original version, which verified the importance of normalization.

Additional Observations

We observed there were only 30 samples with "intphaseendyear" before 2001, including 14 samples with year 1900 (presumably representing missing data). We overwrote the year to 2001 for all these samples. We also noticed the age units could be in days, weeks, months, and years, therefore, the age value columns need to be standardized to decimal years.

We observed an interesting clustering structure, when "Sponsor p2 positive" was plotted against "Sponsor p2 total" (Figure S12). Sponsors were clearly divided into two clusters, where those sponsors in Cluster B seemed to perform rather poorly for their first 2000 trials, but later were able to boost performance, albeit with slightly lower success rates (smaller slope). Eight-nine percent of the sponsors maintained a relatively high phase 2 success rate throughout the years. This phenomenon remained true when the test dataset was merged. We decided to engineer a binary cluster label as a new feature based on this observation, nevertheless, the new feature did not contribute significantly in the end.

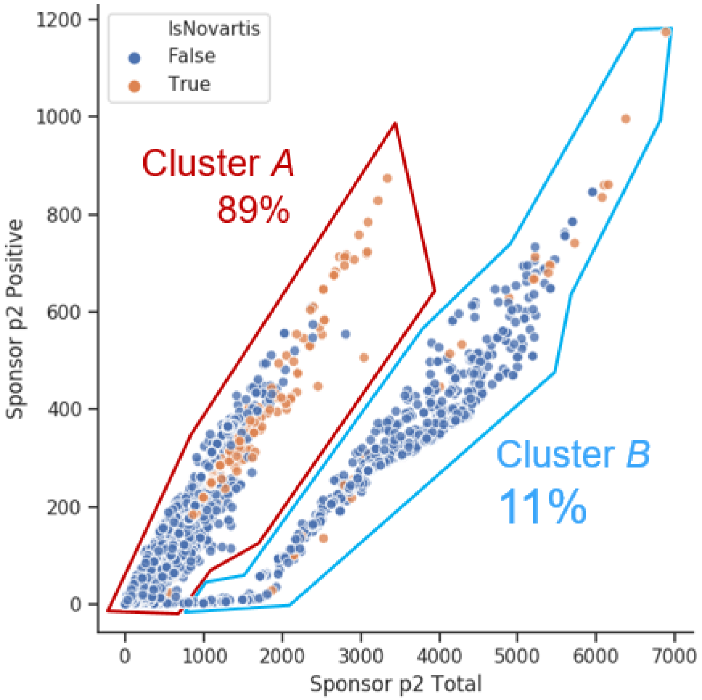

Figure S12. Sponsors fell into two intriguing clusters.

Feature Engineering

Categorical Feature Encoding

There are many categorical features in the PoS dataset, e.g., drug key, indication key, indication group key, sponsor ID, person ID, etc. These are integer identifiers, i.e., their numerical ordering carry no meaning. These features could seriously mislead XGBoost into creating nonsense tree splits, if they were treated as integers. Due to their high cardinality, they should not be treated with one-hot encoding either, otherwise, the resultant large sparse matrices would be hard to learn. Our strategy was to replace these features by their statistics. Considering an example drug key *d* (Figure S13) of a given trial sample completed in year *k*, we collected all trials where *d* was studied and then counted the percentage of successful trials (Figure S13). Success rate was calculated only using samples with year *k*-1 and earlier to avoid any data leaking. As the result, drug key *d* could be encoded with this prior success rate, a continuous and meaning number for XGBoost to split and sort. Similarly, we also encoded drug keys based on phase 3 success rate, completion rate, progress rate, etc.

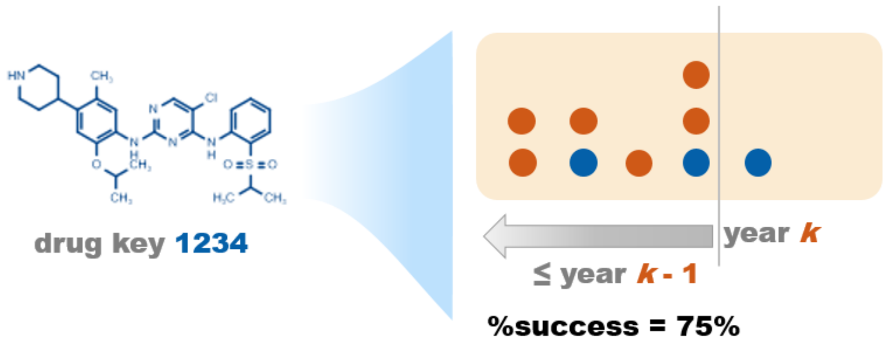

Figure S13. A drug key id 1234 occurred in year *k* can be replaced by its success rate calculated within all records until year *k* - 1.

This encoding technique led to many effective new features. Among all the features provided by the organizer, the feature most correlated with the outcome is called “intpriorapproval” with a Pearson correlation coefficient of 0.28 ($p = 0.04$). Among our engineered new features, “drug prior trial positice.pct” has a Pearson correlation coefficient of 0.31 ($p < 10^{-10}$), presumably better than all other raw features (Figure S14). Retrospectively, among the top 20 most important features, 7 came from normalization, 9 were newly engineered, and only 4 were from the raw given features (Figure S14). These counts might vary between runs but it showed the value of feature normalization and categorical feature encoding.

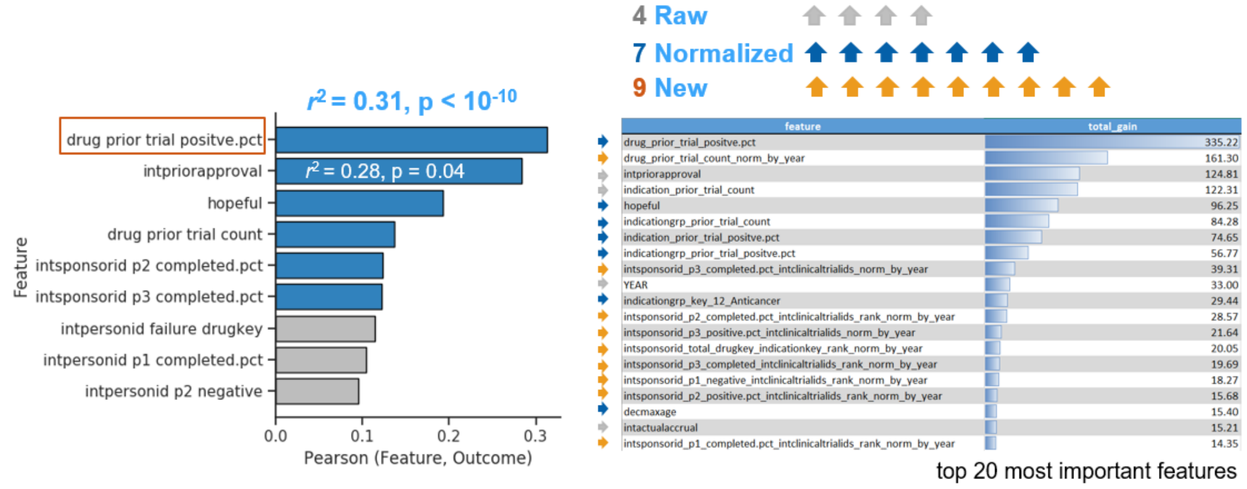

Figure S14. (a) Features most correlated with outcome based on absolute Pearson correlation coefficients. (b) Sources of top 20 features, where the majority came from our feature engineering efforts.

For those multi-label features with fewer discrete values, such as “diseaseType,” we used one-hot encoding. Binary features that were identical for more than 99% of samples were ignored. A total of 607 categories were extracted from 13 multi-label features. Free text columns were transformed into features based on TFIDF (term frequency-inverse document frequency). However, text features did not add much value to our model in terms of score improvement.

Feature Selection

Our experience showed XGBoost could handle several hundred features without problem, we nevertheless applied a feature reduction workflow to remove obviously redundant features generated by normalization or the large number of less informative features derived from text features. The process is illustrated in Figure S15.

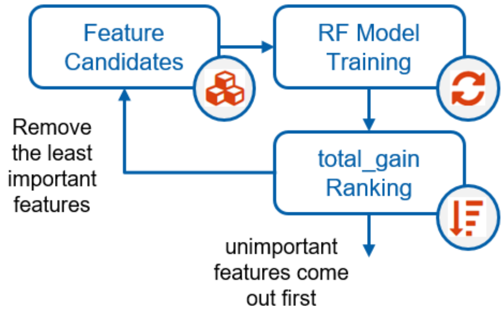

Figure S15. Feature reduction workflow.

We first ranked features by their importance, based on which highly correlated but less important features (Pearson $r^2 > 0.95$) were removed. For feature ranking, we started from all n candidate features, and used them to train a random forest model. We computed the total_gain of each feature and dropped out the feature with the least total_gain. For the remaining n - 1 features, we repeated the previous process to drop out one more feature and n - 2 retained. We iterated until only 1 feature was left. The n features, therefore, were ranked based on the order of their drop out, with the most important features surviving the longest.

For the group of three normalized features, the one with the longest survival was retained. Text features also went through the same triaging process and combined with surviving numerical features for final modeling. We excluded features that might be considered as a leak, e.g., we did not include "generic name," however, post-competition research showed it would have been acceptable. The final model used 275 features. Be aware that random forest, instead of XGBoost, was used in the feature selection process, as its total_gain was considered more reliable since all its tree members were equally important.

Model Evaluation

Cross-validation Strategy

We tested two training-validation splitting strategies. The first strategy was a random five-fold cross validation. The splitting was done in a way that ensured trials sharing the same drug–indication key pair always resided within the same fold and never spanned across training and validation sets. This was to avoid data leaking. The second strategy was to use all drug–indication pairs that did not appear before year 2013 as validation records. Although the second method was a more authentic split mimicking the architecture in the competition (use past to predict the future), it did not perform well compared to the first approach. Presumably, losing the many 2013–2014 records in the training made it hard for the model to capture rules that better described records in the more distant future 2015–2017, as one would expect rules to evolve over the years. Therefore, our final solution adopted the five-fold cross-validation scheme.

Sampling Weighting in Loss Function

We tried to assign weights to trials sharing the same drug–indication pair. We observed there could be many trials studying the same drug–indication simultaneously. For example, erlotinib was studied by about

160 trials (indication 124) in the test set and oxaliplatin was stuided by over 60 trials (indication 141) in the training set (Figure S16). Therefore, we were concerned about overfitting due to some popular drug–indication pairs exerting too much influence during model training. Three trial weighting strategies were tested. First, each trial was weighted by 1. This way a trial–indication pair with *m* trials contributed *m* times during the training, a potential bias we would like to supress. Second, each trial was weighted by 1/*m*, so each trial–indication pair was counted equally in the loss function. This enhanced the sample diversity. Third, each trial was weighted by $1/\sqrt{m}$, so each drug–indication pair had a weight of $\sqrt{m}$, a compromise between the previous two strategies. The performance varied between training runs and we did not see a statistically consistent advantage of 1/*m* or $1/\sqrt{m}$, compared to the simple weighting scheme, therefore no weight was used.

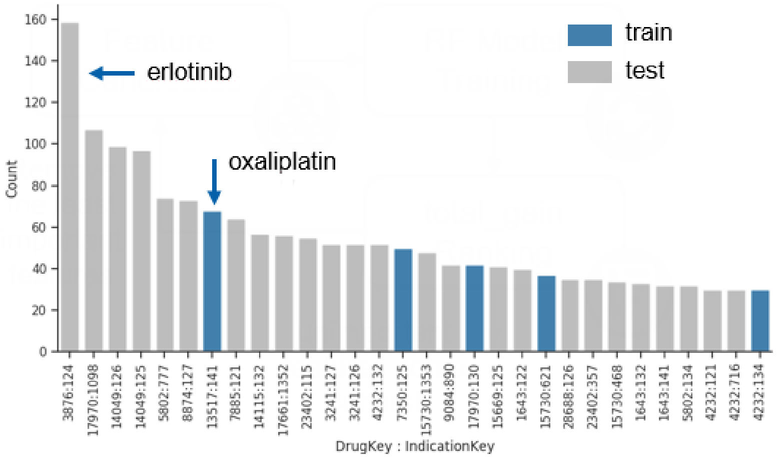

Figure S16. Many drug–indication pairs are heavily studied by multiple trials. Conceptually, this led to bias in training, as well as bias in the leaderboard scoring.

Missing Value Imputation

We also tried two imputation strategies, as this was largely described in the prior publication.[8] The first strategy was to fill the missing value by means. The second was to impute the value using the XGBoost model itself, i.e., we used all other features to build a predictor to predict a target feature containing missing values. Such predictors would use independent features as observed in samples from both training and test datasets. As the XGBoost model can take missing values as its independent variables, unlike random forest models, all independent features other than our target prediction variable, regardless of whether there were missing values, were used in the model training. Although the second strategy had a conceptual advantage, we did not see a consistent difference compared to the first strategy, which was straightforward, thus we chose the first.

Hyperparameter Tuning

The optimal model hyperparameters were identified with grid search based on cross validation. It should be noted that the loss scores used in the search were implemented exactly the same as the final loss function, including all the post processing and augmentation tricks described below.

Model Tuning

Post-processing

Log-loss score is the designated primary leaderboard score. This cross-entropy score heavily penalizes super-confident wrong predictions. For a positive record, improving the prediction probability from 0.99 to $1\text{-}10^{-10}$ only increased the score by 0.01, but reducing the prediction probability from 0.01 to 10^{-10} would decrease the score by 27.6! In other words, there is little reward in making a super-confident correct prediction but there is a huge penalty in making a super-confident wrong prediction. Therefore we clipped all prediction values into range [0.01, 0.99] to avoid severe penalties for mispredictions. The cutoff was determined empirically based on cross validation.

We noticed that 49% of the test records fell into the 2018–2019 period during the model refinement phase. With our domain knowledge, it was clear these samples were extremely unlikely to have positive outcomes, as the time left after the completion of phase 2 trials was too short for a non-trivial approval process. Therefore, we set their probabilities to 0.01.

With feature engineering, five-fold cross validation, and hyperparameter tuning, we were able to build an XGBoost model with the public leaderboard score of 0.275. The text features only had very minor contributions. With the correction of the artifacts in the test set (2018–2019 records), we then obtained a model in the third week with score 0.236.

Drug–indication Augmentation

Our model predicted the probability of success for each trial, i.e., it assigned different probabilities for different trials. However, the truth, the approval/failure outcome, was assigned to each drug–indication pair instead of each trial. The many trials for the same drug–indication pair shared the same outcome truth (Figure S16). This implies we should consolidate the different predicted probabilities for the *m* related trials under the same drug–indication pair into one final probability value.

Mathematically, there is an advantage in unifying the predictions. Considering two related trials share the same positive outcome, with predicted probability $p+\delta$ and $p-\delta$. If we submit them as they are, the score is $\log(p^2-\delta^2)$. If instead we submit their average value for both records, the score is $\log(p^2)$, which is better (the larger, the better here). The conclusion does not change if the outcome is negative.

We did not use average, though. Our reasoning was as long as one of the *m* trials yielded favorable results that successfully demonstrated the efficacy of a drug–indication pair and convinced the authority for an approval, all the remaining m-1 trials would take a free ride and be considered successful regardless of how poorly their trials were conducted. Therefore, our aggregation function was an aggressive max() function across the *m* underlying trials. One out of *m* shots was all it took for an approval. With this drug–indication augmentation, our model score improved to 0.222.

Insights

The exact tree structures embedded in XGBoost models were sensitive to small adjustments in the modeling workflow. We hypothesized this was due to two factors: (1) many features are correlated, therefore, models could achieve very similar performance with different decision rules; and (2) the first few trees in the XGBoost models accounted for the most gain, therefore, there would be a signficant variation in the early tree structures in the XGBoost models. Therefore, we decided not to over-interpret the XGBoost model, but instead derived insights from a random forest model consisting of 200 trees. By averaging the decision rules across these equally-weighted trees, we hoped to extract more reliable insights compared to using XGBoost trees.

Single-feature Analysis

The single-feature importance analysis was implemented in scikit-learn module (https://scikit-learn.org). The top ten features are shown in Figure S17. "Hopeful" is an engineered binary feature replacing "termination_reason." "Drug_prior_trial_positive.pct" is an engineered feature described previously in our categorical feature encoding section, representing the historical (up to year *k*-1) success rate of a drug. "Indicationgrp_prior_trial_count" is the number of trials for that indication group up to year *k*-1. "Targetaccrual" (normalized by year) is a normalized version of "targetaccrual." "Indication_prior_trial_count" is self explanatory. "Priorapproval" is a binary feature provided as it is, which had the highest Pearson correlation coefficient with outcome in the original feature set. "Sponsorid_total_drugkey_indicationkey" is the sum of approval and failure counts. "Minage" is the minimal age of patients. "Actualaccrual" is a feature provided. "Indication_prior_trial_positive.pct" reflects the historical success rate for that indication up to year *k*-1.

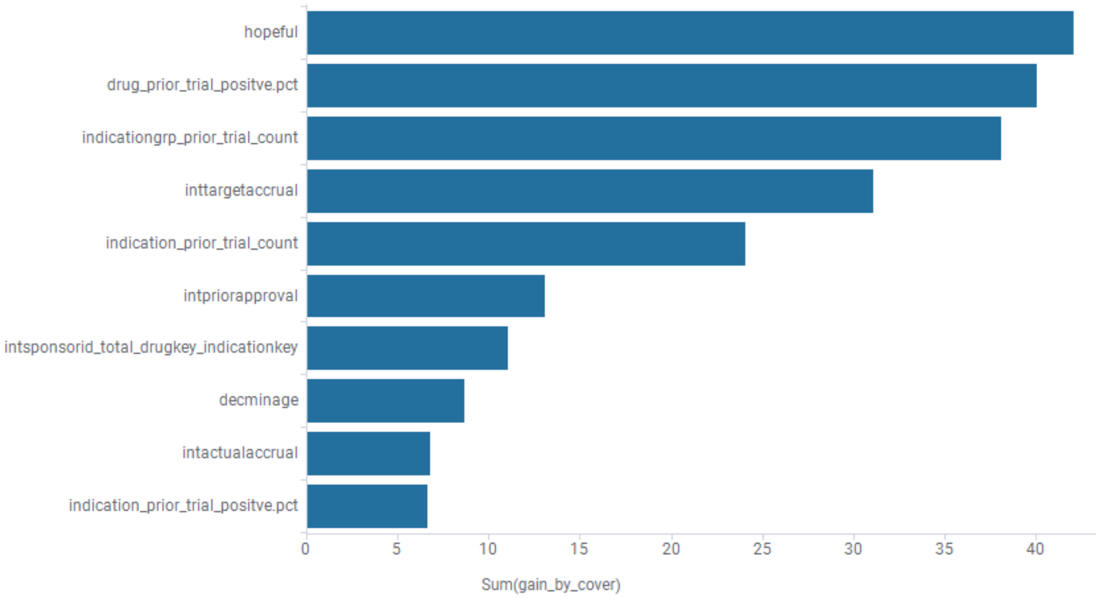

Figure S17. Top 10 most important features based on a random forest model.

As an example, Figure S18 illustrates why "drug_prior_trial_positive.pct" is an important feature. For those drugs with a prior positive rate below 0.48, there is only a 17% chance of a positive outcome versus a remarkable 63%, when the positive rate is above 0.48. The outer ring shows the percentage, if this prior positive rate were irrelevant.

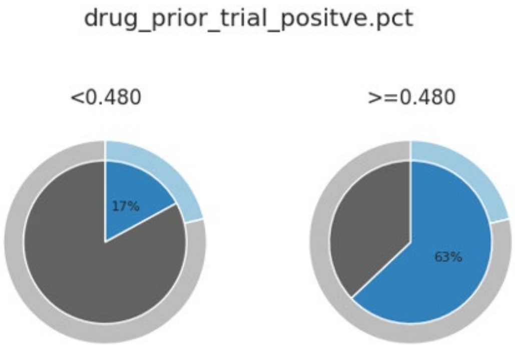

Figure S18. Effectiveness of drug prior positive rate in predicting outcomes.

Figure S19 suggests those trials accepting younger patients (< age 14) tend to be more successful. This might be because companies are generally much more conservative, when it comes to drugs that will be used in kids and sometimes even in infants.

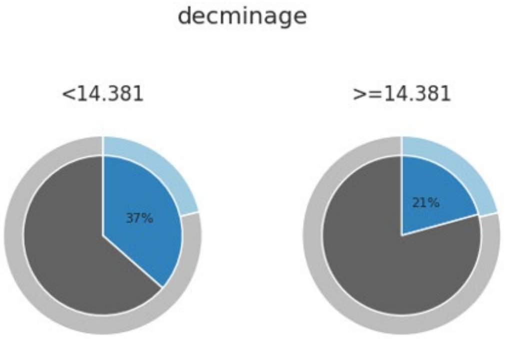

Figure S19. Minimum age of patient in trials can be predictive.

Informative Feature Pairs

As single feature analysis mostly reproduced previously-published results, we aimed to analyze the effect of feature pairs. Similar to analyzing a single feature, we looked at the frequency and the loss contribution of an immediate parent–child feature pair and compiled statistics based on their contributions to the model (as illustrated in Figure S20). This was an idea extended from but not yet implemented in scikit-learn.

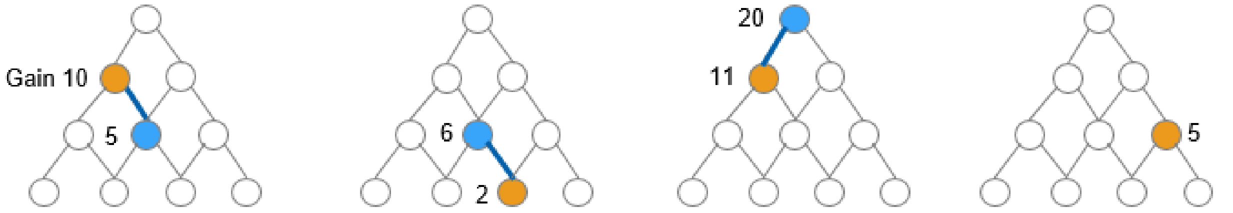

Figure S20. Similar to single feature analysis, a potentially interesting feature pair is parent–child nodes that appear frequently and make important contributions to the gain among a decision forest. The example pair occurs in three out of four trees with an average gain of 13.5.

The network in Figure S21 provides a visual summary of our findings. This network includes all top ten features, as well as some additional features that were not the most important by themselves but became useful in combination with the top features.

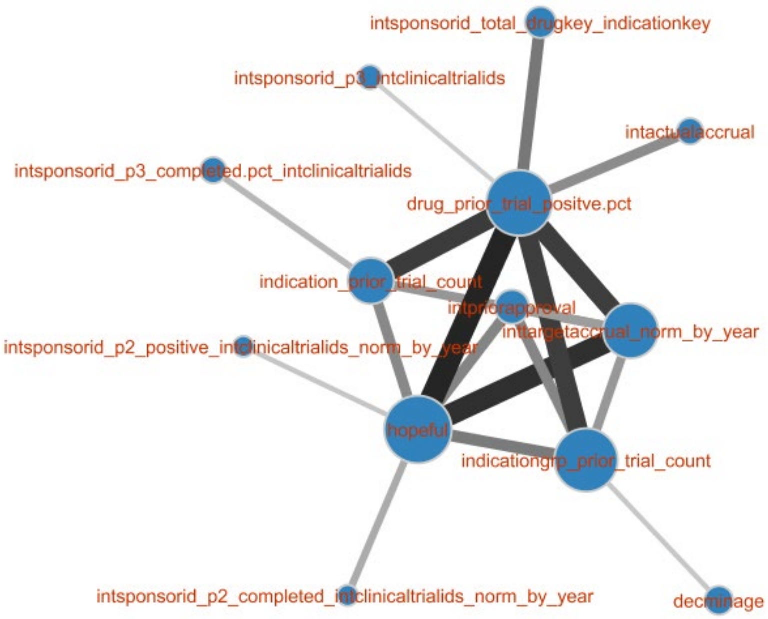

Figure S21. The network of important single features and feature pairs. The size of the node represents the importance of a single feature and the thickness of an edge represents the importance of a feature pair.

Most important feature pairs are intuitive. For example, “hopeful” and “drug_prior_trial_positive.pct” can boost the confidence in a positive outcome. As shown in Figure S22, “drug_prior_trial_positive.pct” alone can predict a positive outcome with 63% accuracy (lower-left pie) and a negative outcome with 83% accuracy (mid-left pie) based on a cutoff of 0.49. However, if this feature were combined with the “hopeful” feature, the model could further improve the accuracy to 70% (lower-right pie) and 96% (center pie), respectively, albeit the percentage of records in these two groups are only 7% and 21% (shown in upper-left corner), respectively. This shows that the combination of two correlated features can lead to higher prediction power.

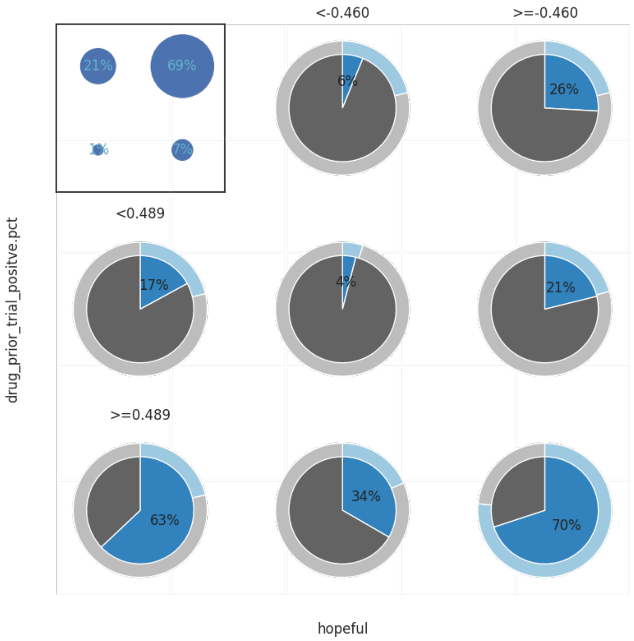

Figure S22. The combination of hopeful and drug prior positive rate can boost prediction.

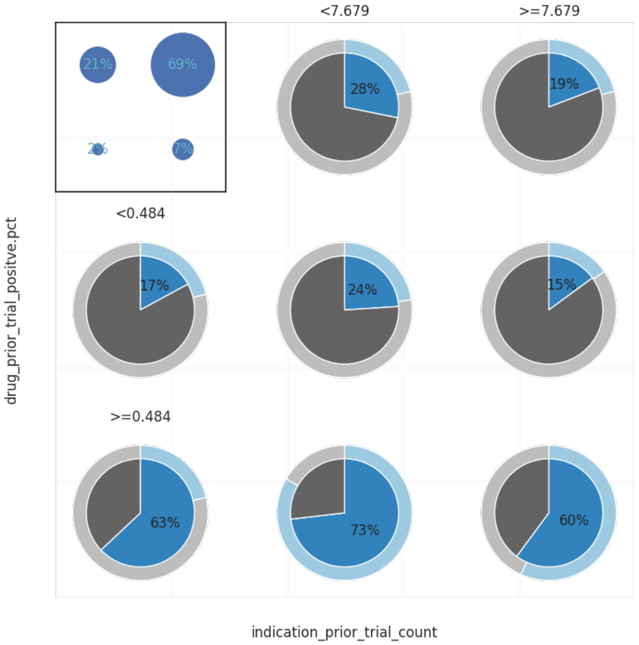

Figure S23. The combination of prior indication trial count and drug prior positive rate can give higher accuracy for applying historically successful drugs in indications that are less explored.

Figure S23 shows that the indications with fewer prior trial counts tend to have a higher success rate (28% vs 19% in the top row). When this is coupled with “drug_prior_trial_positive.pct,” it boosts the accuracy from 63% to 73%.

Although not shown in Figure S21, the combination of “drug_prior_trial_positive.pct” and “anticancer” leads to interesting results summarized in Figure S24. Anti-cancer alone is not a powerful feature, as the positive outcome rate is about the same—21% for “anitcancer” and 22% for “other” indications (top row). However, when we combined it with “drug_prior_trial_positive.pct,” this feature became impactful. The positive outcome rate changes from 65% (lower-left pie) into 54% (lower-middle pie) and 71% (lower-right pie). There appears to be a strong coupling between the two features. Our interpretation is that drugs that have been approved in other indications tend to be more likely to be approved for a new indication, and this empirical rule seems to be more true for cancer than other indications. Presumably, chemotherapies that have already worked in a particular cancer type have a better chance to work in another cancer type. Past history is less likely to predict futrue success for non-cancer indications.

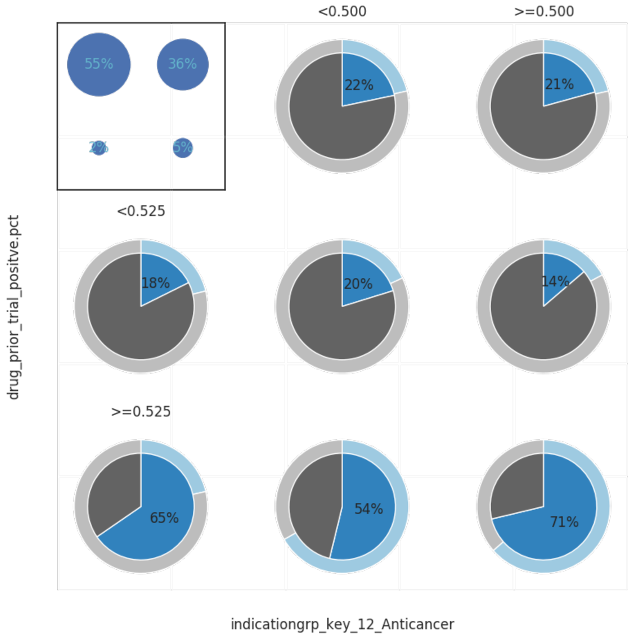

Figure S24. Cancer indication alone is not predictive, but it helps boost accuracy when coupled with drug_prior_trail_positive.pct. This pair is not among the top, probably because the number of records benefiting from this rule is small.

References

1. Chen, T., and Guestrin, C. (2016). XGBoost: A scalable tree boosting system. Proceedings of the ACM SIGKDD International Conference on Knowledge Discovery and Data Mining, 785–794. 10.1145/2939672.2939785.

2. Imai, K., and Ratkovic, M. (2014). Covariate balancing propensity score. Journal of the Royal Statistical Society: Series B: Statistical Methodology 76, 243–263. 10.1111/rssb.12027.

3. Brest, J., Greiner, S., Bošković, B., Mernik, M., and Zumer, V. (2006). Self-adapting control parameters in differential evolution: A comparative study on numerical benchmark problems. IEEE Transactions on Evolutionary Computation 10, 646–657. 10.1109/TEVC.2006.872133.

4. Thakur, A. (2020). Approaching (Almost) Any Machine Learning Problem. (Abhishek Thakur).

5. Pargent, F., Pfisterer, F., Thomas, J., and Bischl, B. (2021). Regularized target encoding outperforms traditional methods in supervised machine learning with high cardinality features. arXiv, arXiv:2104.00629.

6. Slakey, A., Salas, D., and Schamroth, Y. (2019). Encoding Categorical Variables with Conjugate Bayesian Models for WeWork Lead Scoring Engine. arXiv, arXiv:1904.13001.

7. Gore, S. M. (1987). Biostatistics and the medical research council. Medical Research Council News 35, 19–20.

8. Lo, A. W., Siah, K. W., and Wong, C.H. (2019). Machine learning with statistical imputation for predicting drug approval. Harvard Data Science Review 1. 10.1162/99608f92.5c5f0525.
