# Supplementary material for: Predicting drug approvals: The Novartis data science and artificial intelligence challenge
Source: Patterns (N Y). 2021 Jul 21;2(8):100312. doi: 10.1016/j.patter.2021.100312 (PMC8369231; doi:10.1016/j.patter.2021.100312)
Supplement: Document S2. Article plus supplemental information [file mmc3.pdf]

Descriptor

Patterns

Predicting drug approvals: The Novartis data science and artificial intelligence challenge

Highlights

- Data science challenge at Novartis in collaboration with MIT researchers
- Dataset derived from 2 decades of drug development and clinical trial data
- Winning teams outperformed the baseline MIT model by leveraging domain expertise
- Predictive analytics can augment human judgment in drug-development risk management

Authors

Kien Wei Siah, Nicholas W. Kelley, Steffen Ballerstedt, ..., Shifeng Pan, Yingyao Zhou, Andrew W. Lo

Correspondence

alo-admin@mit.edu

In brief

In an in-house data science challenge, Novartis researchers developed machine-learning models for predicting drug-development outcomes, using 2 decades of clinical trial data and building upon previous work at MIT. By leveraging domain expertise, 2 winning teams, out of 50 that participated, developed models that outperformed the baseline MIT model through state-of-the-art algorithms and feature engineering. In addition to providing new insights into drug approvals, the models can augment human judgment to make more informed risk-management decisions.

Siah et al., 2021, Patterns 2, 100312
August 13, 2021 © 2021 The Authors.
https://doi.org/10.1016/j.patter.2021.100312

CellPress

Patterns

CellPress OPEN ACCESS

Descriptor

Predicting drug approvals: The Novartis data science and artificial intelligence challenge

Kien Wei Siah,[1,2] Nicholas W. Kelley,[3] Steffen Ballerstedt,[3] Björn Holzhauer,[3] Tianmeng Lyu,[4] David Mettler,[3] Sophie Sun,[4] Simon Wandel,[3] Yang Zhong,[5] Bin Zhou,[5] Shifeng Pan,[5] Yingyao Zhou,[5] and Andrew W. Lo[1,2,6,7,*]

[1]Laboratory for Financial Engineering, Sloan School of Management, Massachusetts Institute of Technology, Cambridge, MA 02142, USA

[2]Department of Electrical Engineering and Computer Science, Massachusetts Institute of Technology, Cambridge, MA 02142, USA

[3]Novartis Pharma AG, 4056 Basel, Switzerland

[4]Novartis Pharmaceuticals Corporation, East Hanover, NJ 07936, USA

[5]Genomics Institute of the Novartis Research Foundation, San Diego, CA 92121, USA

[6]Sante Fe Institute, Santa Fe, NM 87501, USA

[7]Lead contact

*Correspondence: alo-admin@mit.edu

https://doi.org/10.1016/j.patter.2021.100312

THE BIGGER PICTURE The probability of success is a key parameter that clinical researchers, biopharma executives and investors, and portfolio managers focus on when making important scientific and business decisions about drug development. We describe an in-house data science and artificial intelligence challenge organized by Novartis in collaboration with MIT researchers. Using state-of-the-art machine-learning algorithms and extensive feature engineering augmented by domain expertise in drug development, two winning teams developed models that outperformed the baseline MIT model proposed in a prior study. These new predictive models can be used to augment human judgment to make more informed data-driven decisions in portfolio risk management and capital allocation. These results suggest the possibility of developing even more accurate models using more comprehensive and informative data, and a broader pool of challenge participants.

1 2 3 4 5 **Proof-of-Concept:** Data science output has been formulated, implemented, and tested for one domain/problem

SUMMARY

We describe a novel collaboration between academia and industry, an in-house data science and artificial intelligence challenge held by Novartis to develop machine-learning models for predicting drug-development outcomes, building upon research at MIT using data from Informa as the starting point. With over 50 cross-functional teams from 25 Novartis offices around the world participating in the challenge, the domain expertise of these Novartis researchers was leveraged to create predictive models with greater sophistication. Ultimately, two winning teams developed models that outperformed the baseline MIT model—areas under the curve of 0.88 and 0.84 versus 0.78, respectively—through state-of-the-art machine-learning algorithms and the use of newly incorporated features and data. In addition to validating the variables shown to be associated with drug approval in the earlier MIT study, the challenge also provided new insights into the drivers of drug-development success and failure.

INTRODUCTION

The rising cost of clinical trials and a shift to utilizing more complex biological pathways with greater therapeutic potential—but also greater chances of failure—have caused drug development to become an increasingly lengthy, costly, and risky endeavor in the past decade.[1–5] The average drug now requires at least 10 years of translational research involving multiple iterations of lead optimization and several phases of clinical studies costing hundreds of millions of dollars before it can be approved by drug-regulatory authorities, such as the US Food and Drug Administration (FDA).

Due to the capital-intensive nature of the drug-development process, biotech and pharma companies can only afford to

This is an open access article under the CC BY license (http://creativecommons.org/licenses/by/4.0/).

invest in a limited number of projects. When managing their portfolios of investigational drugs, these developers typically use historical estimates of regulatory approval rates, based on the therapeutic class and phase of development of the drug, combined with subjective adjustments, determined through unstructured discussions of project-specific risk factors, to make their investment decisions. Recently, however, there has been increased interest in combining machine-learning predictions with human judgments on project-specific information in a more structured manner.[6]

In a recent large-scale study involving a range of drug and clinical trial features from over 6,000 unique drugs and close to 20,000 clinical trials, Lo et al.[7] applied machine-learning techniques to predict regulatory approval. Using two proprietary pharmaceutical pipeline database snapshots (taken through 2015Q4) provided by Informa (Pharmaprojects and Trialtrove), Lo et al.[7] developed models that achieved promising predictive accuracy, measured at 0.78 and 0.81 AUC for predicting transitions from phase 2 to regulatory approval and phase 3 to regulatory approval, respectively. (The AUC, also known as the area under the receiver-operating characteristic curve, is the estimated probability that a classifier will rank a positive outcome higher than a negative outcome.) The models also identified the most useful features for predicting drug-development outcomes: trial outcome, trial status, trial accrual, trial duration, prior approval for another indication, and sponsor track record.

With a better understanding of the drivers of drug approval as well as more accurate forecasts of the likelihood of clinical trial success, biopharma companies and investors should be better able to assess the risks of different drug-development projects, and thus allocate their capital more efficiently.

As an extension of the previous study, the authors (all from MIT) collaborated with Novartis, one of the largest multinational pharmaceutical companies in the world, to implement an in-house data science and artificial intelligence (DSAI) challenge based on updated snapshots (taken through 2019Q1) of the same Informa databases. This challenge was designed to leverage the domain expertise of Novartis data scientists, statisticians, portfolio managers, and researchers to develop more powerful models for predicting the probability of success of pipeline drug candidates and uncover deeper insights into the drivers of drug approval. Success in this context was defined as regulatory approval. Over 50 teams consisting of more than 300 individuals from 25 Novartis offices around the world participated in the challenge, submitting approximately 3,000 models for evaluation in a head-to-head competition. In addition to their predictive performance, the teams were evaluated on the innovativeness and robustness of their models, and the potential business value of their findings.

In this paper, we summarize the findings of the top-performing teams. By examining their models, we validate the variables previously found to be associated with drug approval and identify new features that contain useful signals about drug-development outcomes.

Methods

Data

For the DSAI challenge, we used two pharmaceutical pipeline databases from the commercial data vendor Informa for the core dataset: Pharmaprojects, which specializes in drug information, and Trialtrove, which specializes in clinical trial intelligence (see https://pharmaintelligence.informa.com/products-and-services/data-and-analysis/citeline). These two databases aggregate drug and trial information from over 40,000 data sources in the public domain, including company press releases, government drug and trial databases (e.g., Drugs@FDA and ClinicalTrials.gov), and scientific conferences and publications. The database snapshots used in this paper are updated versions of those used in Lo et al.[7] (2019Q1 versus 2015Q4).

As in Lo et al.,[7] we constructed a dataset of drug-indication pairs, focused on phase 2 trial data that have known outcomes ("P2APP"), either successful registration or program termination. We extracted a range of drug compound attributes and clinical trial characteristics as potential features for prediction, including three binary features, one date, seven numerical features, two multi-class features, 16 multi-label features, and five unstructured free texts. These are summarized in Table 1. For the purpose of our analysis, we defined the development status of suspension, termination, and lack of development as "failures," and registration and launch in at least one country as "successes" or approvals (see Note S1 for further details).

The resulting dataset consisted of 6,901 drug-indication pairs and 12,680 unique phase 2 clinical trials, with end dates spanning 1999 to early 2019, containing about two decades of data (Table 2). In our dataset, 796 drug-indication pairs (11.5%) were successes, and 6,105 drug-indication pairs (88.5%) ended in failure. The data cover 15 indication groups: alimentary, anticancer, anti-infective, anti-parasitic, blood and clotting, cardiovascular, dermatological, genitourinary, hormonal, immunological, musculoskeletal, neurological, rare diseases, respiratory, and sensory products. Drugs for cancer, rare diseases, and neurological diseases made up the largest subgroups. As expected, the majority of the trials in the dataset were sponsored by industry rather than investigator-initiated academic trials.

Challenge setup

The DSAI challenge was hosted on an Aridhia Digital Research Environment (Aridhia DRE), a cloud-based platform designed for collaborative data analytics on healthcare data (see https://www.aridhia.com/). Each team was provided a remote workspace for accessing the data, computing resources for developing their models, and a Git repository hosted by AIcrowd for managing their source code (see https://www.aicrowd.com/). AIcrowd was also used to host a leaderboard and discussion forum for teams to interact and answer questions. Figure 1 presents an illustration of the setup.

For the leaderboard challenge, teams were required to predict the probability of regulatory approval (i.e., the drug-indication development status) given phase 2 trial data and drug compound characteristics (see Table 1). This corresponds to a real-world decision-making scenario whereby a pharmaceutical company must decide whether to invest in a phase 3 program based on phase 2 results. We split the P2APP dataset chronologically, with drug-indication pairs that failed or succeeded before 2016 provided to the participants as training data, while those pairs that failed or succeeded in 2016 or later were held out as testing data for leaderboard evaluation. Table 2 shows the sample sizes of the training and testing data. Teams were encouraged to create new features in the core dataset, in

Table 1. Features extracted from Pharmaprojects and Trialtrove

	Description
Drug-indication pair	
Biological target	protein on which the drug acts
Country	country in which the drug is being developed
Drug-indication development status	current approval status of the drug-indication pair
Indication	indication for which the drug is under development
Mechanism of action	mechanism through which the drug produces its pharmacological effect
Medium	physical composition of the material in which the drug is contained
Name	name of the drug
Origin	origin of the active ingredient in the drug
Prior approval of drug for another indication	approval of the drug for another indication prior to the indication under consideration
Route	route by which the drug is administered
Therapeutic class	therapy area for which the drug is in development
Trial	
Attribute	distinguishing attribute or feature of the trial, e.g., registration trials, biomarkers, immuno-oncology
Actual accrual	number of patients enrolled in the trial
Disease type	disease, disorder, or syndrome studied in the trial
Duration	duration of the trial
Exclusion criteria	criteria for excluding a patient from trial consideration
Gender	gender of the enrolled patients
Investigator experience	primary investigator's success in developing other drugs prior to the drug-indication pair under consideration
Location	country in which the trial is conducted
Number of identified sites	number of sites where the trial is conducted
Outcome	outcome of the trial
Patient age	minimum and maximum age of the enrolled patients
Patient population	general information about the disease condition of the enrolled patients
Patient segment	disease segmentation by patient subtypes, therapeutic objectives, or disease progression/staging
Phase 2 end date	year phase 2 ended (end date of the last observed phase 2 trial)
Primary endpoint	detailed description of primary objective, endpoint, or outcome of the trial; endpoints are classified into four main groups: efficacy, safety/toxicity, health economics and outcomes research, and pharmacokinetics/pharmacodynamics
Sponsor	financial sponsor of the trial
Sponsor track record	sponsor's success in developing other drugs prior to the drug-indication pair under consideration
Sponsor type	sponsor grouped by type
Status	recruitment status of the trial
Design	investigative methods used in the trial
Design keywords	keywords relating to investigative methods used in the trial
Target accrual	number of patients sought for the trial
Therapeutic area	therapeutic area of the disease studied in the trial

See Note S1 for examples of each feature.

addition to those provided, by linking new datasets (e.g., compound data) and through feature engineering.

The challenge spanned 5 months, from October 2019 to March 2020: 1 month for team registration and onboarding, 2 months for model development and submission, and 2 months for final evaluation. During the model development segment, teams built their models using the training data. They were able to receive real-time feedback on the performance of their models on a subset of the testing data (50%) and how it compared with other teams ("open-testing round"). This happened via a public leaderboard, which was updated with every submission. This gave participants the opportunity to refine and calibrate their algorithms. Additionally, each team's submissions were evaluated on the complete testing set

Table 2. Sample sizes of the P2APP dataset, and the training and testing data used for the challenge

	Drug-indication pairs	Clinical trials	Unique drugs	Unique indications	Unique clinical trials
Phase 2 to approval (P2APP)					
Success	796	2,435	614	182	2,209
Failure	6,105	13,203	3,313	283	10,722
Total	6,901	15,638	3,726	291	12,680
Training data					
Success	610	1,852	468	169	1,666
Failure	4,293	6,839	2,537	264	5,845
Total	4,903	8,691	2,872	272	7,451
Testing data					
Success	186	583	160	93	557
Failure	1,812	6,364	1,096	218	5,065
Total	1,998	6,947	1,229	229	5,561

Note that the number of unique drugs, indications, and clinical trials are not necessarily additive across rows since drugs, indications, and trials have relationships that are surjective and non-injective: different drugs may target the same indication, and some trials may involve multiple drug-indication pairs. See also Note S1.

(100%) in the final evaluation round. This information was not shown to participants during the competition, defining the private leaderboard to assess performance. We used the binary cross entropy log loss function as the primary scoring metric for evaluating the predicted probabilities.

We also trained a baseline model based on the algorithm described in Lo et al.[7] using the same training data provided to the participants. To obtain the confidence interval of the performance of each model, we bootstrapped the testing set 1,000 times and evaluated the models on the same bootstrapped datasets.

As part of the final evaluation process, teams were required to upload the code used to train their models and a write-up describing their methods and results. An evaluation committee was formed from technical and domain experts to (1) validate the team's leaderboard performance and (2) assess the level of depth regarding business insights produced by the models. Along the technical dimension, each team's source code repository was examined to ensure that the results reported were robust and reproducible. The submission history of the top-performing teams was also reviewed to prevent gamification and ensure that they did not gain an unfair advantage by making frequent submissions. As discussed in the results, little evidence for overfitting or reverse engineering was observed. Technical evaluation also included understanding the innovative aspects of top solutions that were driving their performance in terms of data wrangling and adopted methodology. Domain experts then evaluated the insights and learning from such model interrogations and visualizations in terms of general, scientific program, and scientific trial insights. Since the potential business value of the findings would be to inform portfolio and risk-management decisions, the focus for the business evaluation was on the interpretability of the models, i.e., the ease of insight regarding the risk factors and key drivers of approval. This additional domain assessment was planned in anticipation of a potential discrepancy between top-performing models and actionable insights. However, in-depth domain expertise and feature insights proved to be clear differentiators of both winning solutions.

Subsequent to this evaluation, the two top-performing teams were selected to present their findings to a final committee consisting of Novartis leaders from its portfolio strategy and biostatistics divisions and its Digital Office, and MIT researchers A.W.L. and K.W.S. Other teams with innovative approaches were also invited to share their experience as part of a panel discussion with the broader Novartis community.

RESULTS

We received approximately 3,000 model submissions in the open-testing round of the leaderboard challenge. The teams explored a wide range of machine-learning models, ranging from traditional logistic regression, support vector machines, decision trees, and neural networks to ensemble methods such as random forests,[8] gradient boosting machines, XGBoost,[9] and combinations of multiple types of models.

Recognizing the dangers of overfitting that arise from the reuse of testing set data,[10] we created a scatterplot of public and private leaderboard scores to assess the extent of adaptive overfitting (Figure 2). The public scores were evaluated on a subset of the testing set provided to the participants during the open-testing round, while the private scores were evaluated on the complete testing set in the final evaluation round. In the ideal case, the points would lie close to the diagonal since the public and private performance of the models would be almost identical. In contrast, deviations from the diagonal suggest possible overfitting. We observe that our scores approximated the ideal case in Figure 2, indicating that there was little evidence of DSAI challenge competitors overfitting to the public leaderboard score.

In Figure 3, we compare the performance of the top ten ranking teams with the baseline model described in Lo et al.,[7] using the private leaderboard log loss and the AUC as our metrics. While the baseline model had a worse log loss compared with the top ten best-performing teams, its AUC (0.78 with 95% confidence interval [CI] [0.75, 0.82]) was only lower than the top two teams in the challenge. This may be, in part, because the teams in the competition attempted to optimize log loss.

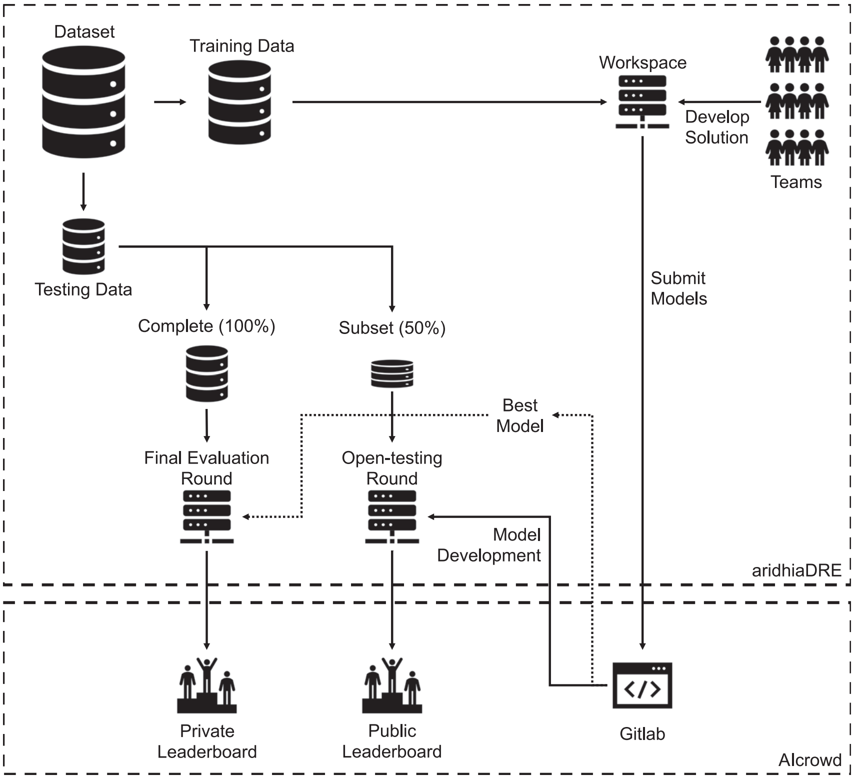

Figure 1. DSAI challenge setup
The challenge was hosted on Aridhia DRE and AIcrowd. It consisted of an open-testing round for teams to refine and calibrate their models, and a final evaluation round.

We focus on the approaches of the two teams that outperformed the baseline model on all metrics. These teams had different strategies and backgrounds of expertise, but were aligned in the way they harnessed human insight into their model predictions.

- The team with the top-ranked model was primarily composed of biostatisticians with significant domain expertise in clinical trial data analysis. They relied on handcrafted features that incorporated their insights into drug-development timelines and which data entries should be discarded. A team member with portfolio management experience also provided a different perspective.
- The runner-up team was primarily composed of data scientists with domain expertise in bioinformatics and chem-informatics. They relied on extensive data exploration and feature engineering, in particular developing a novel method to understand the interaction of these features, but also augmented them with clinical trial knowledge.

Approach of the top-performing team

The top-performing model was developed by a collaborative team (team ''Insight_Out'') from Novartis offices in the United States and Switzerland whose members had backgrounds in biostatistics, data science, and portfolio management. Their model achieved an AUC of 0.88 (95% CI [0.85, 0.90]), corresponding to an improvement of approximately 0.10 over the baseline model. In addition to using the core features provided in the dataset, the team created several new features to: capture information about orphan drug indications, improve the granularity of therapeutic areas; compare the relative size of phase 2 trials with the average by therapeutic area and disease; classify the drug candidate as a novel compound, a life cycle management (LCM) project, or a generic; and determine whether an international nonproprietary name (INN) has been registered for the drug.

The final model of the top-performing team was an ensemble consisting of two XGBoost models and one Bayesian logistic regression (BLR) model. See https://mc-stan.org/rstanarm/ and https://github.com/stan-dev/stancon_talks/for implementation details of the BLR model. The XGBoost models, known to be highly effective for tabular data, were trained using 263 raw and derived features, using time-series cross-validation with different levels of hyperparameter tuning (i.e., using simple heuristics and a more sophisticated approach involving differential evolution optimization).[11] Subsequently, logistic regression with a ridge penalty was used to combine the trial-level predictions of the XGBoost models into predictions at the drug-indication level.

The BLR model was trained using case weights based on covariate balancing propensity scores,[12] with greater weights given to cases that had a greater propensity of appearing in the test set. The BLR model allowed the team to incorporate its judgment on the likely effects of a smaller set of features. These included granular therapeutic areas as a random effect, novelty (e.g., that a drug was non-generic, and not an insulin or a flu vaccine), the relative phase 2 accrual versus the disease average, the success rates of drugs with the same mechanism of action, INN assignment, and trial outcomes, as well as interactions between these features. The parameters were estimated via Markov chain Monte Carlo sampling.

Ensembles of diverse models can generally outperform any individual model.[13] The ensemble predictions were obtained as a weighted average of the predictions from the XGBoost and BLR models. Afterward, the predictions were post-processed using heuristics derived from the team's domain expertise. For example, the predictions for trials after 2018 were rescaled between 0.001 and 0.1 because the team believed that obtaining approval within 2 years of completing phase 2 was unlikely. These limits were determined based on prior elicitation using the roulette method.[14] In addition, the team introduced upper and lower bounds for their predictions to reduce the impact of overconfident and overpessimistic predictions on the log loss, since extreme predictions that are incorrect are heavily penalized under the log loss metric.

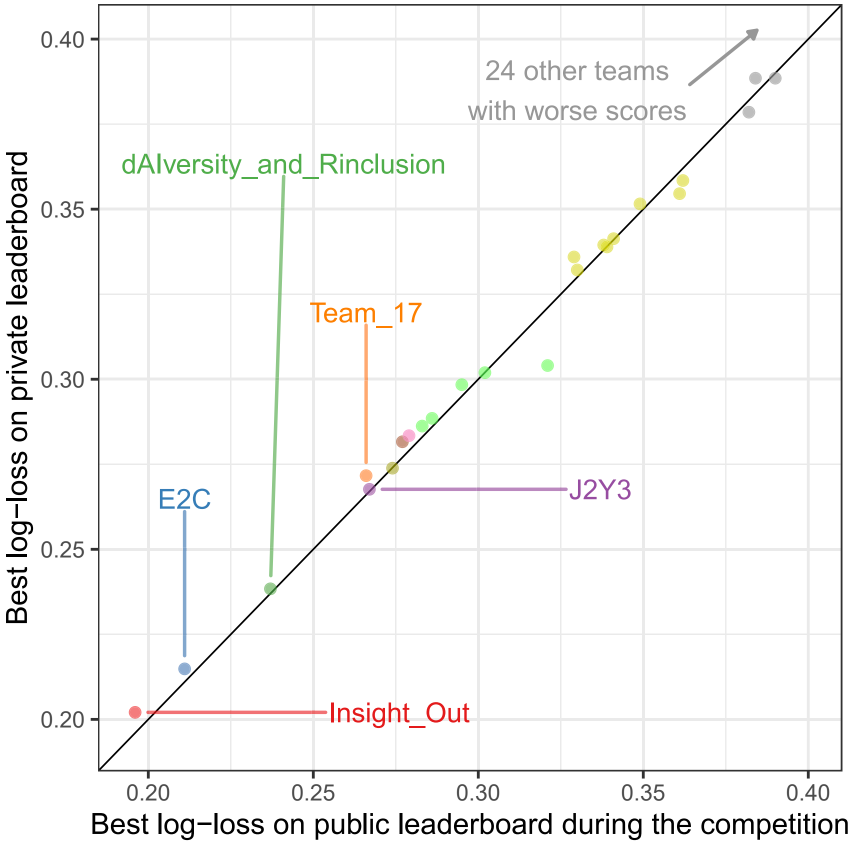

Figure 2. Scatterplot of public and private leaderboard scores
Each point corresponds to the best-performing submission of each team. The points lay very close to the diagonal, which indicates that there is little evidence of overfitting in the competition.

The team found that the phase 2 accrual relative to the disease average was one of the strongest predictors of approval. The likelihood of success increased for programs with above-average accrual compared with other programs for the same disease. In contrast, programs with below-average accrual were more likely to fail. The team also found prior approvals for any indication (e.g., LCM programs), past approvals of other drugs for similar indications, and well-established modes of action improved the odds of approval, suggesting that repositioning an approved drug for a new indication is less challenging than developing a first-in-class new chemical entity. On the other hand, they found that drugs that targeted difficult-to-treat diseases (i.e., therapeutic areas that have historically demonstrated a much lower probability of success in clinical development versus their counterparts), such as cancer or Alzheimer's disease, were more likely to fail. Trial termination (whether due to lack of efficacy, safety issues, or pipeline reprioritization), poor patient enrollment versus planned accrual, and the absence of an INN were also strong indicators of failure. See Note S2 for further details of the methodology and findings.

Approach of the second-place team

The second-place model was developed by a team of data scientists and researchers from the Genomics Institute of the Novartis Research Foundation (team "E2C"). This model achieved an AUC of 0.84 (95% CI [0.81, 0.86]), corresponding to an improvement of approximately 0.06 over the baseline model. The team performed extensive feature engineering, creating rank-normalized versions of features known to demonstrate temporal coupling (e.g., phase 2 trial durations, which have shown greater mean and spread over the years). This was done because decision-tree algorithms tend to be inefficient at incorporating heteroskedasticity. In addition to the core features in the dataset such as prior approval, the team created new variables to capture the impact of development history on future approvals. For example, they computed the number of past trials in which each drug had been involved by phase, by outcome, and in aggregate, regardless of indication. Additionally, they made a similar computation for indications and indication groups, aggregating them over all drugs. The team also used natural language-processing techniques, such as the TFIDF (term frequency-inverse document frequency) algorithm, to convert text data for trials into feature vectors. Because the set of features under consideration was large, the team performed stepwise feature selection using random forests to identify a parsimonious set of factors.

From the outset, the second-place team focused on the XGBoost model, an algorithm that has a strong track record in data science competitions. They explored multiple training-validation strategies for hyperparameter selection, eventually settling on the random 5-fold cross-validation approach. Like the top team, they also post-processed trial-level predictions from the XGBoost model, based on expert knowledge. For example, they reduced the predictions for trials after 2018 because team members believed that approval within 2 years was unlikely, and clipped overconfident and overpessimistic predictions to reduce the impact of outliers on the log loss scoring metric. Unlike the leading team, however, they obtained predictions for each drug-indication pair by using the maximum trial-level prediction across all trials associated with the drug-indication pair, as opposed to using penalized logistic regression. They hypothesized that the best-performing trial would dominate the outcome of the drug-indication pair regardless of any lack of evidence in other trials in support of efficacy.

Among the final set of features, the second-place team found that rank-normalized variables were generally favored over their raw, unnormalized counterparts, thus verifying the importance of normalization. Out of the top 20 most important features, eight were novel features created by the team and not provided in the core dataset (see Note S3 for further details). They found that the top features were largely consistent with those reported

CellPress
OPEN ACCESS

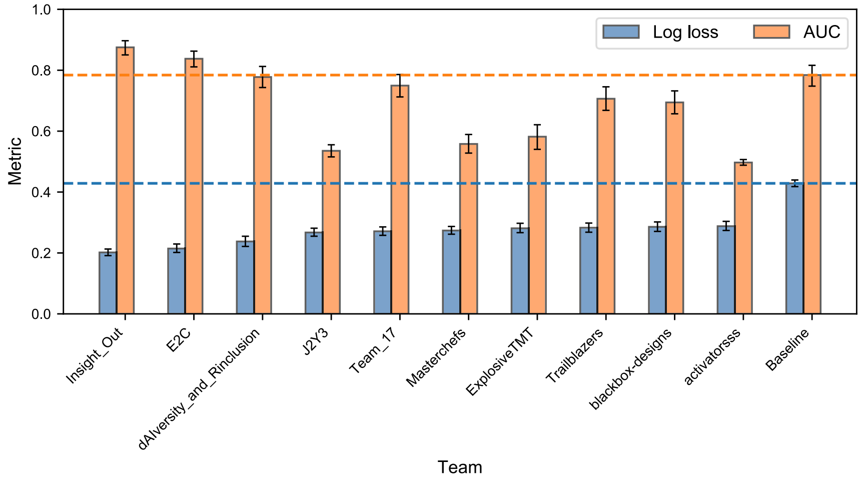

Figure 3. Private leaderboard log loss and AUC for the top ten ranking teams and the baseline model

The error bars correspond to the 95% confidence intervals. The top two teams outperformed all other submissions in the leaderboard challenge as well as the baseline model.

by Lo et al.,[7] e.g., trial outcomes, trial accrual, prior approval, and sponsor track record. Moreover, they found that drugs with strong development histories, as quantified by the percentage of past trials with positive outcomes, were more likely to be successful. Over- and underenrollment with respect to the target accrual were also associated with lower success rates, not an entirely unexpected finding since these signs hint at poor trial operation or a lack of efficacy. Interestingly, the team found that trials with a younger age criterion for inclusion tended to be more successful. However, features created from text data did not seem to contribute meaningful predictive value.

In addition to single-feature analysis, the second-place team went a step further to identify informative feature pairs. They found strong interaction effects between trial outcomes and drug-development history, e.g., the historical success rate of past trials and the presence or absence of prior approval. For example, given a successful trial with its primary endpoints met, a drug with prior approval for other indications was almost twice as likely to be approved in comparison with a new compound without any prior approval. The team also found that drug developers with a strong track record had higher probabilities of success in indications that had been less explored in the development process, as quantified by the cumulative number of past trials.

In addition, the team observed strong coupling between the success of anti-cancer drugs and their development history. The likelihood of success of an anti-cancer drug was five times greater with a prior approval than without. This effect was less pronounced in non-cancer programs, where the ratio in success rates conditional on prior approval was only twice as great. The team hypothesized that historical success rates and prior approval were especially important for anti-cancer drugs because it is not uncommon for effective cancer therapies to work across multiple cancer subtypes (e.g., chemotherapy), and therefore an approval in one subtype was predictive of potential success in other subtypes. See Note S3 for further details of the methodology and findings.

We also evaluate the performance of both models when combined, taking simple composites of the winning teams' predictions, e.g., the maximum, minimum, arithmetic mean, and geometric mean. The combined models achieved AUCs that are close to the top-performing model, ranging between 0.86 and 0.88 depending on the composite used.

DISCUSSION

MIT and Novartis researchers collaborated on an in-house DSAI challenge to develop machine-learning models for predicting clinical development outcomes, building on Lo et al.,[7] whose work used one of the largest pharmaceutical pipeline databases in the world, provided by Informa. To the best of our knowledge, this challenge represents the first crowd-sourced collaborative competition to use pharmaceutical data for this purpose, in this case updated snapshots of the same Informa databases used in the earlier MIT study. In total, over 50 cross-functional teams from 25 international Novartis offices participated in the challenge. We received approximately 3,000 model submissions over a 2-month period.

Internal data science competitions are both an opportunity for a company to address business problems and a learning opportunity for the company's data science community. From this perspective, the large number of Novartis associates who chose to actively participate in the process and had the chance to expand their data science skill set was encouraging.

The probability of success is one of several key parameters, in combination with unmet medical need and market opportunity, which clinical researchers, biopharma investors, and portfolio managers consider when making scientific and business decisions about drug development. Accurate estimates of this parameter are therefore critical for efficient risk management and resource allocation. The top-performing teams in their winning solutions delivered additional heuristics with respect to predicting the probability of success:

- Identification of novel features predictive of probability of success (as outlined above)
- Novel approaches and methodologies for feature extraction, combining domain expertise and machine learning
- Creative ways of introducing additional data types to the problem, such as unstructured text and biochemical data—for example, several teams presented ways of connecting new data types, although this in itself did not translate into top leaderboard performance

Additionally, the discussion about the availability of specific information at the time of decision making about the fate of a project was helpful for assessing the potential for target leakage in the solutions of external vendors offering similar predictive solutions.

However, the DSAI challenge also had several limitations. First, the P2APP dataset was split chronologically, using drug-indication pairs that failed or succeeded before 2016 as training data, and those that failed or succeeded in 2016 or later were held out as testing data. Due to the nature of drug development, however, some boundary effects were inevitably present in the last years of the testing data. Because drugs tend to fail much more quickly than those that are approved, the majority of the trials completed after 2018 ended in failure. (The probability of phase 2 to approval in the testing data is 9.3% for all trials in aggregate but only 1.8% for trials completed after 2018.) With their experience and expertise in drug development, both teams eventually discovered this artifact in the data, and were able to improve their model performance by adjusting their predictions for trials after 2018. While such adjustments were useful in the competition, they add little practical value for real-life application.

Second, some available features reflected a decision already taken by a company to terminate a project. These included trials that were stopped due to pipeline reprioritization, a small-sized phase 2 program due to stopping the program after an initial small trial, and the failure to apply for an INN. Not all such information is available at the time of decision making in practice. These limitations illustrate that in order to make data science competitions directly useful for business problems without substantial modification, it is important to align extremely closely the prediction task in the competition with the real-world business problem

We also received feedback from knowledgeable participants that the core dataset lacked key information that decision-makers typically take into consideration, such as the preclinical data, detailed safety and efficacy data, and biological plausibility of the mechanism of action. Unfortunately, investigators do not usually release this information to the public domain for strategic reasons. It is therefore unsurprising that such data are not available in commercial pharmaceutical databases based on publicly available sources of information. Potentially, this limitation could be overcome by recent progress in deep-learning approaches to natural language processing, which may enable information about trial protocols, development programs, and drugs to be extracted from unstructured text data sources.

Conclusion

By tapping the power of crowd-sourcing and the domain expertise of Novartis researchers working in cross-disciplinary teams, we have shown the potential for DSAI challenges to generate predictive models for drug-development outcomes that outperform existing models from the academic literature. In addition to validating features previously associated with drug approval in the MIT study, the DSAI challenge has provided new insights into the drivers of drug approval and failure. Ultimately, these new predictive models can be used to augment human judgment to make more informed decisions in portfolio risk management. Nevertheless, there remains a clear opportunity to further improve the models in this competition. We believe that more accurate models can be developed with access to better quality and more comprehensive data, and a broader pool of challenge participants.

EXPERIMENTAL PROCEDURES

Resource availability

Lead contact

Andrew W. Lo, MIT Sloan School of Management, 100 Main Street, E62–618, Cambridge, MA 02142. (617) 253-0920 (tel), (781) 891-9783 (fax), alo-admin@mit.edu (email).

Materials availability

This study did not generate new unique reagents.

Data and code availability

The source code of the algorithms is available at https://github.com/bjoernholzhauer/DSAI-Competition-2019 (Insight_Out) and https://github.com/data2code/DSAI-Competition-2019 (E2C). The data supporting the current study have not been deposited in a public repository, due to their proprietary nature. The data are available from Informa at https://pharmaintelligence.informa.com/products-and-services under Pharmaprojects and Trialtrove. Restrictions apply to the availability of the data, which were used under license for this study.

SUPPLEMENTAL INFORMATION

Supplemental information can be found online at https://doi.org/10.1016/j.patter.2021.100312.

ACKNOWLEDGMENTS

Research support from the MIT Laboratory for Financial Engineering is gratefully acknowledged. We thank Informa for allowing us to use their data for this project, three anonymous referees for specific comments on the manuscript, and Jayna Cummings for editorial assistance. We would also like to acknowledge the operational team behind the DSAI challenge—Srayanta Mukherjee, Preeti Chauhan, Pamoli Dutta—for the final dataset and, along with Imtiaz Hossain, for the Python starter kit; Markus Lange and Conor Moloney for the R starter kit and as part of the evaluation team along with Srayanta and Shantanu Bose; Lisa Hampson and Mark Baillie as our biostatistics and clinical trial/program probability of success experts; Adrian Cassidy, Holger Hoefling, and Peter Krusche for the initial testing of the platform; and Satya Kantipudi for managing this complex project. The views and opinions expressed in this article are those of the authors only, and do not necessarily represent the views and opinions of any institution or agency, any of their affiliates or employees, or any of the individuals acknowledged above. No direct funding was received for this study; general research support was provided by the MIT Laboratory for Financial Engineering and its sponsors. The authors were personally salaried by their institutions during the period of writing (though no specific salary was set aside or given for the writing of this paper).

AUTHOR CONTRIBUTIONS

Conceptualization, K.W.S., N.W.K., S.B., and A.W.L.; Resources, N.W.K., S.B., and A.W.L.; Methodology, K.W.S., N.W.K., S.B., B.H., T.L., D.M., S.S., S.W., Y. Zhong, B.Z., S.P., Y. Zhou, and A.W.L.; Software, K.W.S., B.H., T.L., D.M., S.S., S.W., Y. Zhong, B.Z., S.P., and Y. Zhou; Formal analysis, K.W.S., B.H., T.L., D.M., S.S., S.W., Y. Zhong, B.Z., S.P., and Y. Zhou; Writing – original draft, K.W.S. and A.W.L.; Writing – review & editing, K.W.S., N.W.K., S.B., B.H., T.L., D.M., S.S., S.W., Y. Zhong, B.Z., S.P., Y. Zhou, and A.W.L.; Supervision, N.W.K., S.B., and A.W.L.; Project administration, N.W.K., S.B., and A.W.L.; Funding acquisition, N.W.K. and A.W.L.

DECLARATION OF INTERESTS

K.W.S., N.W.K., S.B., B.H., T.L., D.M., S.S., S.W., Y. Zhong, B.Z., S.P., and Y. Zhou declare no competing interests. A.W.L. reports personal investments in biotechnology companies, biotech venture capital funds, and biotech mutual funds (all listed on his homepage) and is a co-founder and partner of QLS Advisors LLC, a healthcare investment advisor, and QLS Technologies Inc., a healthcare analytics and consulting company. A.W.L. is also an advisor to the ACS's BrightEdge Impact Fund, Apricity Health, Aricari Bio, the CME's Competitive Markets Advisory Council, Fennec Health, Lazard, the Swiss

Finance Institute's Scientific Council, and Thalēs; a director of Annual Reviews, Atomwise, BridgeBio Pharma, and Roivant Sciences; and chairman emeritus and senior advisor to AlphaSimplex Group. Finally, A.W.L. is a member of the NIH's National Center for Advancing Translational Sciences Advisory Council and Cures Acceleration Network Review Board.. During the most recent 6-year period, A.W.L. has received speaking/consulting fees, honoraria, or other forms of compensation from AlphaSimplex Group, Annual Reviews, the Bernstein Fabozzi Jacobs Levy Award, BIS, BridgeBio Pharma, Cambridge Associates, CME, Financial Times, Harvard Kennedy School, IMF, JOIM, Lazard, National Bank of Belgium, New Frontiers Advisors (for the Harry M. Markowitz Prize), Q Group, Research Affiliates, Roivant Sciences, and the Swiss Finance Institute.

Received: February 19, 2021
Revised: April 26, 2021
Accepted: June 21, 2021
Published: July 20, 2021

REFERENCES

1. Hopkins, A.L., and Groom, C.R. (2002). The druggable genome. Nat. Rev. Drug Discov. *1*, 727–730. https://doi.org/10.1038/nrd892.
2. Pammolli, F., Magazzini, L., and Riccaboni, M. (2011). The productivity crisis in pharmaceutical R&D. Nat. Rev. Drug Discov. *10*, 428–438. https://doi.org/10.1038/nrd3405.
3. Booth, B., and Zemmel, R. (2004). Prospects for productivity. Nat. Rev. Drug Discov. *3*, 451–456. https://doi.org/10.1038/nrd1384.
4. Ma, P., and Zemmel, R. (2002). Value of novelty? Nat. Rev. Drug Discov. *1*, 571–572. https://doi.org/10.1038/nrd884.
5. Scannell, J.W., Blanckley, A., Boldon, H., and Warrington, B. (2012). Diagnosing the decline in pharmaceutical R&D efficiency. Nat. Rev. Drug Discov. *11*, 191–200. https://doi.org/10.1038/nrd3681.
6. Hampson, L.V., Bornkamp, B., Holzhauer, B., Kahn, J., Lange, M.R., Luo, W., Cioppa, G.D., Stott, K., and Ballerstedt, S. (2021). Improving the assessment of the probability of success in late stage drug development. arXiv, 2102.02752.
7. Lo, A.W., Siah, K.W., and Wong, C.H. (2019). Machine learning with statistical imputation for predicting drug approval. Harv. Data Sci. Rev. *1*. https://doi.org/10.1162/99608f92.5c5f0525.
8. Breiman, L. (2001). Random forests. Mach. Learn. *45*, 5–32. https://doi.org/10.1023/A:1010933404324.
9. Chen, T., and Guestrin, C. (2016). XGBoost: a scalable tree boosting system. In Proceedings of the ACM SIGKDD International Conference on Knowledge Discovery and Data Mining, pp. 785–794. https://doi.org/10.1145/2939672.2939785.
10. Roelofs, R., Shankar, V., Recht, B., Fridovich-Keil, S., Hardt, M., Miller, J., and Schmidt, L. (2019). A meta-analysis of overfitting in machine learning. In Advances in Neural Information Processing Systems, *32*, H. Wallach, H. Larochelle, A. Beygelzimer, F. d'Alché-Buc, E. Fox, and R. Garnett, eds., *pp. 9179–9189*.
11. Brest, J., Greiner, S., Bošković, B., Mernik, M., and Zumer, V. (2006). Self-adapting control parameters in differential evolution: a comparative study on numerical benchmark problems. IEEE Trans. Evol. Comput. *10*, 646–657. https://doi.org/10.1109/TEVC.2006.872133.
12. Imai, K., and Ratkovic, M. (2014). Covariate balancing propensity score. J. R. Stat. Soc. Ser. B: Stat. Methodol. *76*, 243–263. https://doi.org/10.1111/rssb.12027.
13. Thakur, A. (2020). Approaching (Almost) Any Machine Learning Problem (Abhishek Thakur).
14. Gore, S.M. (1987). Biostatistics and the medical research council. Med. Res. Counc. News *35*, 19–20.

Patterns, Volume *2*

Supplemental information

Predicting drug approvals: The Novartis data science and artificial intelligence challenge

Kien Wei Siah, Nicholas W. Kelley, Steffen Ballerstedt, Björn Holzhauer, Tianmeng Lyu, David Mettler, Sophie Sun, Simon Wandel, Yang Zhong, Bin Zhou, Shifeng Pan, Yingyao Zhou, and Andrew W. Lo

Supplemental Experimental Procedures

Supplemental Note S1: Core Dataset

We constructed our datasets using two Informa® databases: *Pharmaprojects* and *Trialtrove*, two separate relational databases organized by largely different ontologies. We extracted drug-specific features and drug–indication development status from *Pharmaprojects*, and clinical trial features from *Trialtrove*.

First, we identified all drug–indication pairs with known outcomes in *Pharmaprojects*. Next, we dropped pairs that did not have any trials captured in *Trialtrove*. (We note that the disease coverage in *Pharmaprojects* and *Trialtrove* is slightly different.) Because missingness is present in both *Pharmaprojects* and *Trialtrove*, we imposed several additional filters to make sure that all samples collected were usable for analysis.

We summarize the steps in Table S1 and Figure S1. It is important to note that the drug, indication, and trial relationships in the databases are surjective and non-injective: different drugs may target the same indication, and some trials may involve multiple drug–indication pairs. This is to be expected since one drug can be indicated for multiple diseases, a disease can have more than one treatment, and it is not uncommon for a trial to involve two or more related primary investigational drugs. In Figure S2, we plot the probability of phase 2 to approval over time in the dataset.

We extracted drug compound attributes and clinical trial characteristics from *Pharmaprojects* and *Trialtrove*, respectively (see Table S2). In addition to structured features readily available in the databases, we created an augmented set of variables that captured sponsor track record and investigator experience. To quantify a particular trial sponsor's track record in successfully developing other drugs, we used the number of prior approved and failed drug–indication developments; and for past trials for phases 1, 2, and 3 separately, we used the total number of trials sponsored, the number of trials sponsored with positive and negative results, and the number of trials sponsored to completion and termination. We used the end date of the last trial of the drug–indication pair under consideration as the cutoff for considering prior experience since the last end date will be the time of prediction. We abstracted investigator experience in the same manner.

Lastly, we also constructed a binary drug–indication pair feature that indicates whether a drug has previously been approved for another indication. Similarly, we used the end date of the last trial as the cutoff for considering prior approval.

Table S1. Filters for constructing P2APP.

	Rationale
**Drug–indication Pairs in *Pharmaprojects***	
Trials observed in *Trialtrove*	We excluded pairs for which we do not observe any trials in *Trialtrove*.
Known approval date (if approved)	We defined the approval date as the earliest date a drug–indication pair was approved in any market. We require these dates to perform time-series analysis.
Known failure date (if failed)	We defined the failure date as one year after the end-date of the last phase 2 or phase 3 trial (if any), whichever is latest.
**Clinical Trials in *Trialtrove***	
Phase 2 trials	P2APP focuses on phase 2 trial data
Known end date	We required these dates to create sponsor track record and investigator experience, and to perform time-series analysis.
Known sponsors and disease types	Trials not tagged with sponsor/disease types are typically out of *Trialtrove* commercial coverage and not maintained.

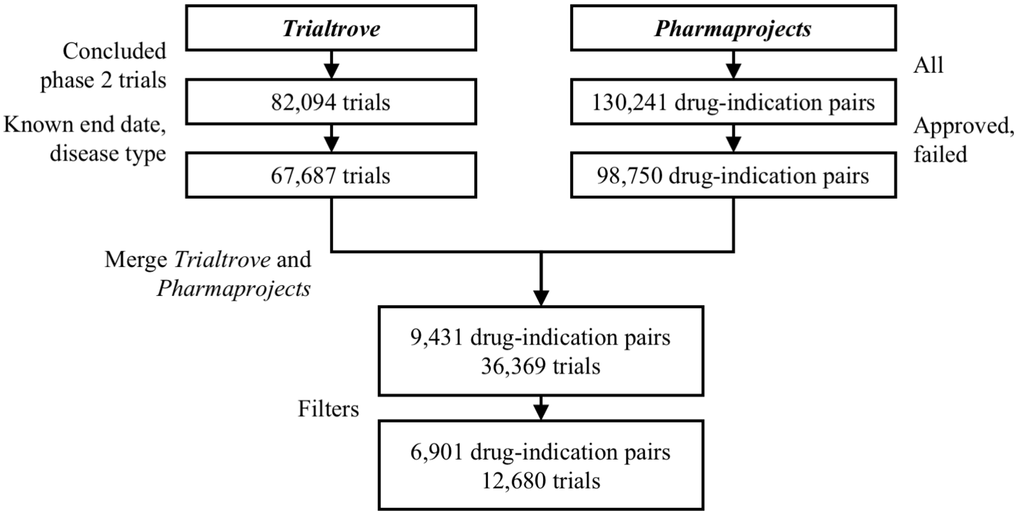

Figure S1. Sample sizes at each step of data pre-processing.

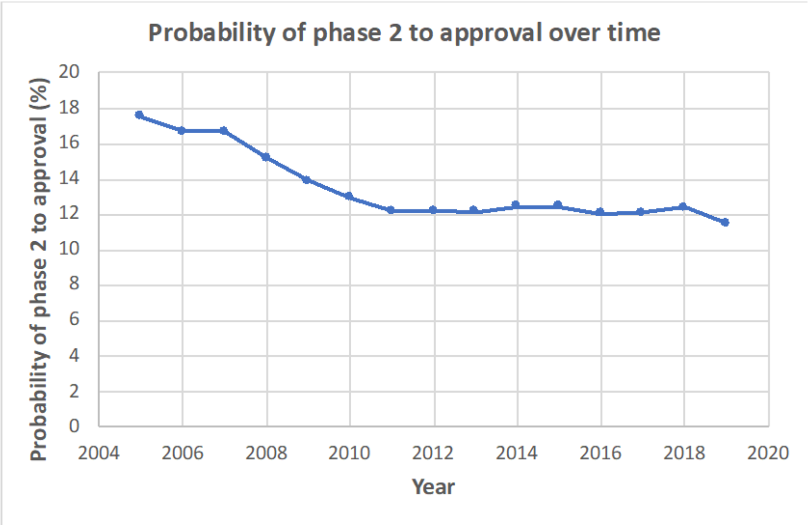

Figure S2. Probability of phase 2 to approval in the dataset plotted using expanding windows.

Table S2. Features extracted from *Pharmaprojects* and *Trialtrove*. We define multi-class features as categorical features that have mutually exclusive categories, i.e., features can belong to only one category at any time; and multi-label features as features that have non-mutually-exclusive categories, i.e., features can belong to more than one category simultaneously.

	Examples	Type
**Drug–indication Pair**		
Biological target	Cytokine/Growth factor; Enzyme; Ion channel; Receptor; Transporter	Multi-label
Country	China; India; Japan; United States	Multi-label
Drug–indication development status	Approved; Failed	Binary
Indication	Cancer, lung, small cell; Cancer, lung, non-small cell; Cancer, brain	Multi-class
Mechanism of action	Cell cycle inhibitor; DNA inhibitor; Ion channel antagonist; Protein kinase inhibitor	Multi-label
Medium	Capsule, hard; Capsule, soft; Powder; Solution; Suspension; Tablet	Multi-label
Name	Free text	String
Origin	Biological, protein, antibody; Biological, protein, recombinant; Chemical, synthetic	Multi-label
Prior approval of drug for another indication	True; false	Binary
Route	Inhaled; Injectable; Oral; Topical	Multi-label
Therapeutic class	Anti-viral, anti-HIV; Anti-cancer, immunological; Anti-epileptic	Multi-label
**Trial**		
Attribute	Biomarker/Efficacy; Biomarker/Toxicity; Pharmacogenomic - Patient Preselection/Stratification	Multi-label
Actual accrual	Integer	Numerical
Disease type	Bladder; colorectal; ovarian	Multi-label
Duration	Integer	Numerical
Exclusion criteria	Free text	String
Gender	Male, female, both	Multi-class
Investigator experience	Refer to sponsor track record	Numerical
Location	Canada; Europe; United Kingdom; United States	Multi-label
Number of identified sites	Integer	Numerical
Outcome	Completed, Negative outcome/primary endpoint(s) not met; Completed, Outcome indeterminate; Completed, Positive outcome/primary endpoint(s) met; Terminated, Safety/adverse effects	Multi-label
Patient age	Integer	Numerical
Patient population	Free text	String
Patient segment	Stage I; stage III; stage IV; second line; pediatric	Multi-label
Phase 2 end date	Date	Date
Primary endpoint	Free text	String
Sponsor	Duke University Medical Center; National Institutes of Health; Celgene	Multi-label
Sponsor track record	Number of prior approved drug–indication pairs; Number of prior failed pairs; Total number of phase 1 trials sponsored; Number of phase 1 trials with positive results; Number of phase 1 trials with negative results; Number of completed phase 1 trials; Number of terminated phase 1 trials; Total number of phase 2 trials sponsored; Number of phase 2 trials with positive results; Number of phase 2 trials with negative results; Number of completed phase 2 trials; Number of terminated phase 2 trials; Total number of phase 3 trials sponsored; Number of phase 3 trials with positive results; Number of phase 3 trials with negative results; Number of completed phase 3 trials; Number of terminated phase 3 trials	Numerical
Sponsor type	Academic; Industry, all other pharma; Industry, Top 20 Pharma	Multi-label
Status	Completed; terminated	Binary
Design	Free text	String
Design keywords	Cross over; Double blind/blinded; Efficacy; Multiple arm; Open label; Pharmacodynamics; Pharmacokinetics; Placebo control; Randomized; Single arm	Multi-label
Target accrual	Integer	Numerical
Therapeutic area	Autoimmune/Inflammation; Cardiovascular; CNS; Infectious Disease	Multi-label

Supplemental Note S2: Detailed Overview of Top-Performing Team

High-level Overview

A high-level overview of the solution by the team in top performing place is shown as a flowchart in Figure S3. The solution uses an ensemble of three different predictions followed by some post-processing. (Source code available at https://github.com/bjoernholzhauer/DSAI-Competition-2019.)

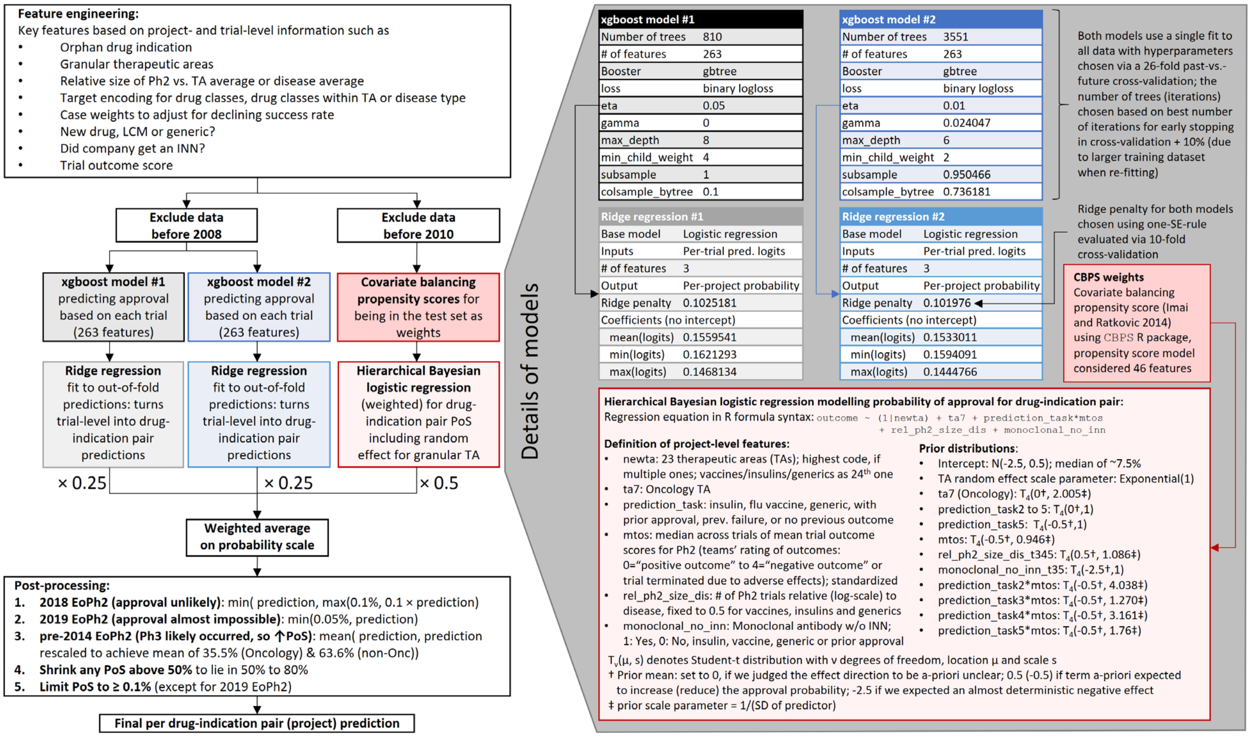

Figure S3. High-level flowchart of top performing team.

Extreme gradient boosting (XGBoost) was found to perform very well, as to be expected on tabular data.[1] Therefore the final solution included two XGBoost models that were fit on trial-level data. To obtain a single prediction per drug–indication pair, the predictions for each phase 2 trial for the drug–indication pair were summarized using 3 features (the mean, minimum, and maximum of the predicted probabilities on the logit-scale) and combined into a single prediction using a ridge regression model. The ridge regression models were fit to the predictions of the validation-fold of each of the 26 cross-validation splits (out-of-fold predictions). Hyperparameters for the ridge regression model were chosen based on the one-standard-error rule via a 10-fold cross-validation. The ridge regression model had the desirable additional effect of improving the calibration of predicted probabilities, which we expected to improve the binary log-loss on the unseen test set data.

In contrast, the third model in the ensemble was a hierarchical Bayesian logistic regression (BLR) model fit on the drug–indication level using features summarized across the trials for the drug–indication pair. The `rstanarm` R package (see https://mc-stan.org/rstanarm/) that uses the Stan modelling language and Markov Chain Monte Carlo sampler (https://mc-stan.org) in the background were used to fit the model.

For the Bayesian model, somewhat informative (i.e., expressing considerable uncertainty about the prior judgements) prior distributions were chosen based on judgments on the likely effects of different features. While this model performed slightly worse than the XGBoost models as assessed by cross-validation (CV), it substantially improved the performance of our final model ensemble.

The observations used in fitting the BLR model were given weights based on covariate balancing propensity scores (CBPS) for being in the leaderboard test set. This had the motivation that there appear to be notable differences in the predictors of the training data and predictors of the leaderboard test set. While we did not know the outcomes to be predicted for the test samples and how exactly these might be influenced by this distribution shift in predictors, we assumed that it would be important to address this. CBPS weights are one of the possible techniques for addressing such a distribution shift.[2]

There is a notable decline in approval rates over time in the training data. Some of this trend may be due to an increasing difficulty in obtaining approval for a new drug. However, we were concerned that this might be the result of failed projects missing from the database for the years before trials had to be registered on clinicaltrials.gov in 2007. We tested this theory by discarding some of the training data both via cross-validation and in terms of leaderboard score, and obtained improved results. Thus, we did not use data before 2008 in our XGBoost models and, in the case of the BLR, even discarded data from 2008 and 2009.

Hyperparameter Tuning for XGBoost

Hyperparameter tuning was based on a 26-fold past-vs.-future CV scheme.

- For the first XGBoost model it was done manually using the rule of thumb that one should tune hyperparameters separately using relatively high learning rate (eta=0.1) in the following order: 1) max_depth, 2) subsample, 3) min_child_weight, 4) colsample_bytree.
- For the second XGBoost model, hyperparameters were tuned at a high learning rate using the differential evolution algorithm[3] implemented in the `DEoptim` R package for global optimization of the leader-board metric as assessed by CV.

For both XGBoost models the final learning rate was 0.05 and the number of trees (learning rounds) for refitting with the whole data was identified using the CV-log-loss (with the optimal number of trees increased by 10% for refitting on the whole dataset to account for the larger dataset).

The selected final hyperparameters are shown in Figure S3.

In contrast to the XGBoost models, the modelling decisions for the Bayesian logistic regression were taken based on the team judgments, because this resulted in substantially better public leaderboard performance than attempts to automatically tune the hyperparameters of prior distributions.

Cross-validation

An appropriate validation set-up is critical for making modelling choices in a principled way that avoids overfitting[4] – i.e., for arriving at a solution that generalizes well to the unseen leaderboard outcomes. The CV approach for hyperparameter tuning followed known general principles for CV (see also https://www.fast.ai/2017/11/13/validation-sets/ accessed 6 April 2021) by

1) attempting to approximate the actual prediction task as closely as possible by splitting the time series of data by past-versus-future (while still offering multiple splits that are as independent as possible for evaluation) and
2) ensuring that studies from the same drug–indication pair are never in the training data and the validation data of a split.

We mixed several possible ways of doing past-vs.-future splits in the 26 different folds we created. These ideas included:

- overlapping past-vs.-future splits (e.g., <2012 vs. >= 2012, <2013 vs. >= 2013, etc.),

- splitting the same future into several bits (e.g., fold 1 = 1/5 of >= 2012, folds 2 = the next 1/5 of >= 2012, etc.), and
- variants where you either try to emulate predicting for the same drug and/or predicting for previously unseen drugs (one could look at those two tasks separately).

As to be expected, random splits correlated less with the public leaderboard score than splitting past-vs.-future.

For the ridge regression, a 10-fold CV scheme on the out-of-fold predictions was used. This CV scheme always grouped all records for a drug–indication pair for any of the 26-validation folds into the same fold.

Feature Engineering

A wide range of additional features (or predictors) was derived in addition to those already provided by the competition organizers. The definition of each of the 263 features used by two XGBoost models is summarized in Table S3. How these features were summarized into features at the drug–indication pair level for the BLR model is summarized in Figure S3.

We highlight a few key features that had high variable importance in the XGBoost models and had somewhat more complex derivations.

One set of key features captured the relative size (on the log-scale) of the phase 2 program (rel_log_size_dis: rel_ph2_size_ta) for the drug–indication pair relative to other drug–indication pairs within the same therapeutic area and for the same disease type. Size was either in terms of the patient number or in terms of the number of phase 2 trials.

Prior approvals for a drug, but also for other similar drugs that share a mode of action is clearly a potentially useful feature. However, for the latter, important information is lost if we focus on just the proportion of prior approvals for similar drugs: 1 approval for one drug is not the same as 20 approvals for 20 drugs—in the latter case our confidence about a mode of action should be increased compared to the first scenario. Similarly, one failure with a previous drug may not be very informative, but repeated failures of many drugs would be. To capture this information, we used two approaches:

1) **Approval counts:** The simpler of the two approaches was to count the number of approvals for a mode of action up to and including the year of the phase 2 end of a drug–indication pair.
2) **Target encoding:** We used a form of target encoding[5,6] adapted to the time series nature of the data by creating a target encoding based only on data up to and including the year of the phase 2 end of a drug–indication pair.

We did this for any approval, approvals in the same therapeutic area and for the same disease type. A drug may have multiple modes of action, in which case we averaged the target encodings for the different modes of action. The derived variable is “prior_approval” in Table S3. A more sophisticated way of dealing with different modes of action could be a direction for future research.

While only limited information on the outcomes of phase trials was available, we created a trial end score to summarize this information (termreason:trailendscore4). Within each trial, one feature was the mean of these scores, while another feature was the worst score.

Other important features included whether a drug is a monoclonal antibody (is_mab), a generic (is_a_generic), an insulin (insulin), or a flu vaccine (fluvacc), and whether an international non-proprietary name (INN) (unwilling_to_pay_12k) is on the dataset for a drug (based on regular expression to distinguish brand names and/or company internal project codes from official INNs).

Table S3. Definitions of the trial-level features used in XGBoost models.

[See Excel file.]

Fitted Models

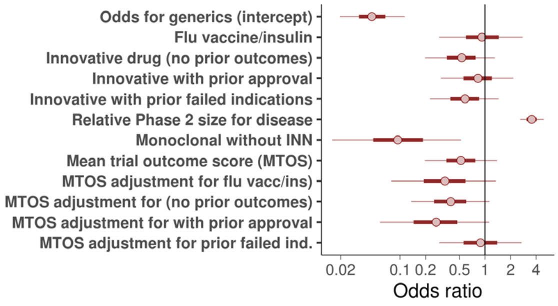

Figure S4. Odds ratios for non-disease area variables from the Bayesian logistic regression used in the final submission.

Figure S4 and Figure S5 show the marginal posterior odds-ratios for each feature obtained from the BLR model. These figures are split according to whether model terms are fixed effects (Figure S4) or random disease area effects (Figure S5). The shown point estimates are posterior medians, the inner intervals are 50% credible intervals, and the outer intervals are 95% credible intervals.

Firstly, a phase 2 program with more trials compared with other drugs for the same disease is associated with a higher probability of approval. This may partially reflect that larger phase 2 programs could result in better decisions about phase 3, but to a considerable extent reflects that projects that fail early in phase 2 will tend to have fewer phase 2 trials than projects that succeed in initial trials. That is, this variable may reflect a decision by companies to terminate a project.

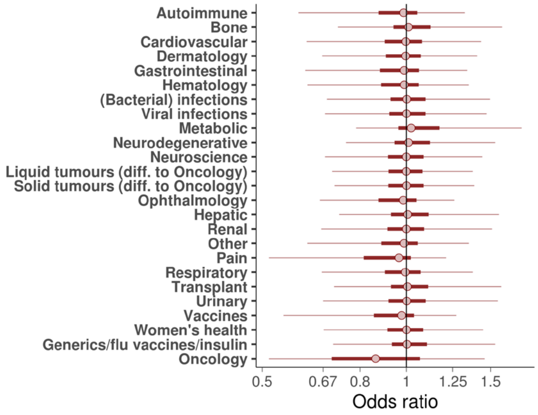

Figure S5. Odds ratios for disease area variables from the Bayesian logistic regression used in the final submission.

Prior approvals for other indications increase the probability of approval. This is logical: A prior approval shows that a drug has at least some clinical effect and makes unexpected safety findings less likely.

If there is a well-understood mode of action and regulatory pathway—such as, for flu vaccines and insulins—the probably of approval is higher, too.

On the negative side, innovative drugs have a lower probability of success than generics. This reflects that the uncertainty about them is greater.

As would be expected, the mean trial outcome score (MTOS) we created is a major predictor of success: projects with studies stopped due to pipeline re-prioritization, strategy shift, safety issues, or lack of efficacy rarely lead to an approval. Similarly, when a company does not obtain an INN for a drug, it does not intend to start a phase 3.

We also note that oncology projects tend to fail more often than drugs in other therapeutic areas.

Three different feature importance metrics for the two XGBoost models are shown in Table S4 for the top-25 predictors in the XGBoost models. As can be seen, no single feature dominates the feature importance, but several features that capture the relative size of the phase 2 program, the target encodings for modes of action, and the trial outcomes scores dominate this list. The lack of a single feature having much higher importance is partially due to the high correlation between the large number of related features.

Table S4. Feature importance for top 25 predictors in XGBoost models.

Feature	XGBoost model #1			XGBoost model #2		
	Gain	Cover	Freq.	Gain	Cover	Freq.
rel_ph2_size_ta: Relative size (number of trials) of phase 2 for TA	0.095	0.056	0.046	0.221	0.118	0.075
rel_ph2_size_dis: Relative size (number of trials) of phase 2 for disease	0.090	0.053	0.048	0.078	0.062	0.061
meanclu50: Avg. target encoding for MoAs of drug (UCrL of 50% CrI)	0.065	0.052	0.051	0.046	0.049	0.055
class_approvals: Total # of MoAs of drug with previous approvals	0.044	0.042	0.033	0.043	0.063	0.047
meancll50: Avg. target encoding for MoAs of drug (LCrL of 50% CrI)	0.031	0.032	0.035	0.049	0.055	0.062
dtmeancll50: Avg. targ. enc. for MoAs of drug for disease type (LCrL of 50% CrI)	0.052	0.033	0.039	0.066	0.036	0.038
dtmeanclu50: Avg. targ. enc. for MoAs of drug for disease type (UCrL of 50% CrI)	0.059	0.037	0.040	0.049	0.038	0.033
tameancll50: Avg. target encoding for MoAs of drug for TA (LCrL of 50% CrI)	0.062	0.041	0.048	0.022	0.024	0.036
tameanclu50: Avg. target encoding for MoAs of drug for TA (UCrL of 50% CrI)	0.034	0.037	0.038	0.028	0.036	0.045
class_counts: # of prev. approvals with MoAs of drug	0.026	0.028	0.036	0.023	0.030	0.040
rel_log_size_ta: Relative size (number of patients) of phase 2 for TA	0.021	0.027	0.040	0.024	0.022	0.047
rel_log_size_dis: Relative size (number of patients) of phase 2 for disease type	0.022	0.034	0.039	0.016	0.030	0.033
intduration: phase 2 duration	0.014	0.028	0.031	0.017	0.029	0.042
taclass_counts: Total # of approvals for MoAs of drug for TA	0.026	0.026	0.037	0.015	0.014	0.028
dtclass_counts: # of prev. approvals with MoAs of drug for disease type	0.024	0.025	0.029	0.019	0.023	0.026
time_since_first_outcome: Years since first approval or failure for the drug?	0.019	0.023	0.016	0.024	0.030	0.022
pct_accrual: Proportion of target accrual that was actually enrolled in trial?	0.011	0.021	0.030	0.009	0.015	0.029
intactualaccrual: Number of patients actually enrolled in trial	0.010	0.022	0.027	0.010	0.018	0.025
inttargetaccrual: Number of patients planned to be enrolled in trial	0.010	0.019	0.028	0.012	0.015	0.024
phaseendyear: phase 2 end year	0.013	0.015	0.021	0.019	0.015	0.023
termreason: Worst out of ranking of trial termination reasons	0.009	0.011	0.009	0.025	0.042	0.013
unwilling_to_pay_12k: Does the drug have an INN?	0.017	0.016	0.005	0.021	0.033	0.008
taclass_approvals: Total # of MoAs of drug with previous approvals in TA	0.015	0.018	0.024	0.008	0.005	0.012
dtclass_approvals: Total # of MoAs of drug with prev. approvals for disease	0.014	0.013	0.015	0.006	0.005	0.008
mean_trialendscore: Mean score for trial outcome reasons	0.014	0.013	0.010	0.007	0.012	0.004

Post-processing

The post-processing described below was the part of the overall solution that had the largest impact on the leaderboard score. We failed to obtain a satisfactory correlation of model performance estimated via cross-validation and the leaderboard scores until we introduced the post-processing of predictions for projects with an end of phase 2 in 2018 and 2019.

The post-processing reflects that the database provided to competitors was a snapshot taken in mid-2019. For phase 2 studies that ended at some point in 2019, the team considered it highly unlikely that the drug could be approved by mid-2019, because of regulatory approval timelines. Thus, we limited the predicted probability for those studies to be, at most, 0.05%.

For an end of phase 2 in 2018, an approval by mid-2019 is at least possible, but would likely only occur for rare cases that justify approval based on phase 2 data. Thus, we scaled predicted probabilities to lie between 0.1% and 10%. These limits were based on using the discrete mixture distribution of the prior distributions elicited from 4 of the 5 team members. Individual judgments were elicited using the roulette method[7] as shown in Figure S6. A decision analysis using the mixture distribution was then conducted using the log-loss metric used in the competition as the utility function.

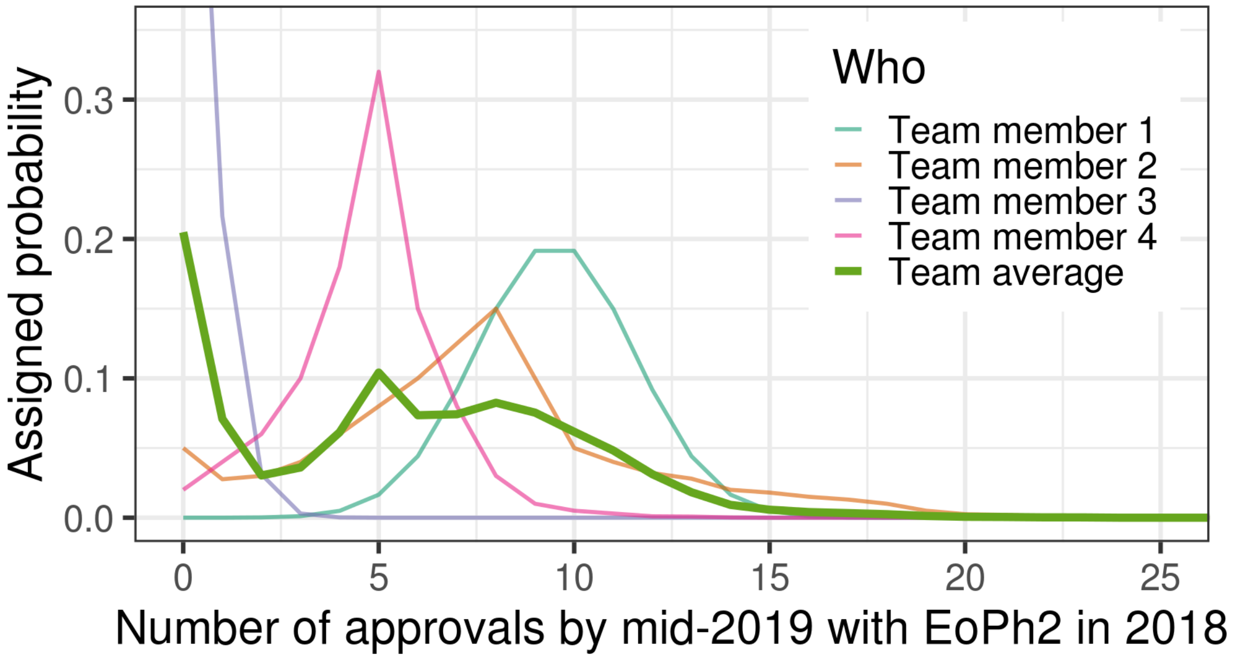

Figure S6. Elicited team prior opinions on the number of approvals by mid-2019 with end-of-phase 2 year 2018 (N=438) conditional on the outcome being known by mid-2019.

From the information communicated on how data were split into training data vs. data for the leaderboard, it seemed that a project could only have an end-of-phase 2 before 2014 and part of the leaderboard data, if a phase 3 program was initiated. For such projects we scaled the probability of approval up to be in line with phase 3 success rates.

We also avoided overly confident predictions. Given the limited available information, it appeared questionable whether predicted probabilities of approval should be above 80%, so we scaled high predicted probabilities downwards.

Additionally, we avoided (except for cases of end-of-phase 2 in 2019) predicted probabilities below 0.1%, because the binary-log-loss competition metric only rewards being right about such predictions to a very limited extent, but severely penalizes being wrong about them.

Ensemble

Figure S7 illustrates why an ensemble of the BLR with the two XGBoost models was effective. While the BLR performed slightly less well in CV and on the public leaderboard, the performance difference was relatively small and the test set predictions of the two model classes had a quite low correlation of about 0.5, which will generally help the performance of an ensemble.[4]

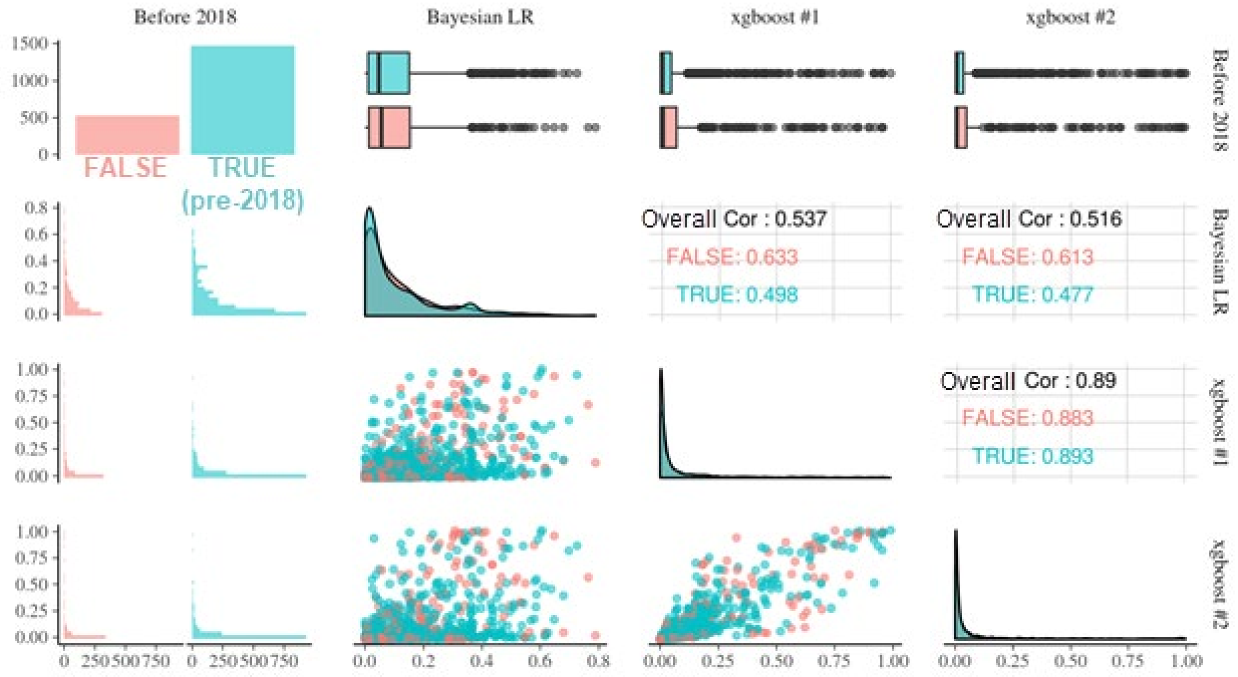

Figure S7. Correlation and distribution of predicted probabilities per drug–indication pair on the test set (overall and for pre-2018 vs. 2018 onwards).

Supplemental Note S3: Detailed Overview of Second Place Team

Overview

The overview of the E2C model development workflow is outlined in Figure S8. The team spent the first week in exploratory data analysis, focusing on visualizing the data and understanding the meaning of each given feature. This stage was critical for discovering artifacts in the data, as well as generating ideas for feature engineering. In the second week, the team carried out extensive feature engineering, explored two training-validation splitting strategies, and constructed the first decent-performing model. In the third week, the team focused on hyperparameter tuning and then further improved the model with two post-processing ideas: (1) bias correction for 2018–2019 samples; and (2) test-time augmentation across trials within the same drug–indication group. The remaining time was mostly invested in gaining insights from the model. We will highlight the key learnings from each step of the workflow. (Source code available at https://github.com/data2code/DSAI-Competition-2019.)

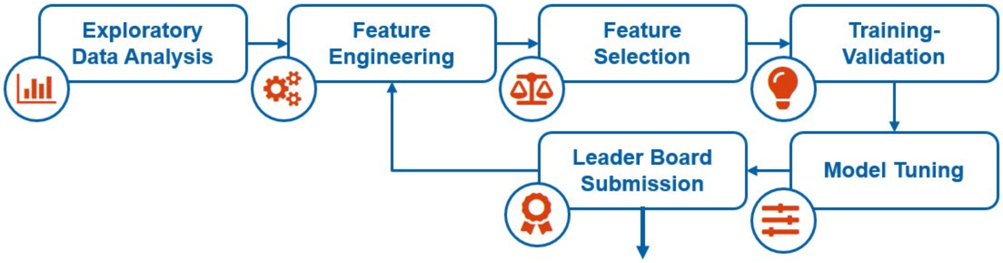

Figure S8. Overview of the E2C model development workflow.

Exploratory Data Analysis

Machine Learning Framework

We locked on using the XGBoost machine learning method early on, as it had been most frequently adopted by winning teams in Kaggle competitions on similar structured datasets. Deep learning framework was excluded, because the probability-of-success training dataset size was likely too small to train a high-performance neural network model without overfitting.

Time-decoupling

Many features were known to demonstrate strong coupling with time, as described in previous studies (e.g., Lo et al. 2019). We were able to validate those, e.g., the phase 2 trials tend to run longer and longer over the years (Figure S9).

XGBoost utilizes a decision tree structure, which is inefficient to capture such a coupling effect. Pretending trials within the top quantile of each year have a higher probability of success, as this upper quantile threshold drifts over the years. XGBoost would need multiple tree levels to handle the different thresholds across years (Figure S10). Therefore, to facilitate model training, we decided to remove the coupling between features and year through a normalization process. Normalization produced two additional features based on each raw input feature. Take feature "*abc*" as an example: (1) "*abc*_norm_by_year" was the normalized version using the mean and standard deviation of all feature values within that particular

year; (2) “*abc*_rank_norm_by_year” was its non-parametric quantile counterpart within the year, which was not sensitive to the underlying distribution.

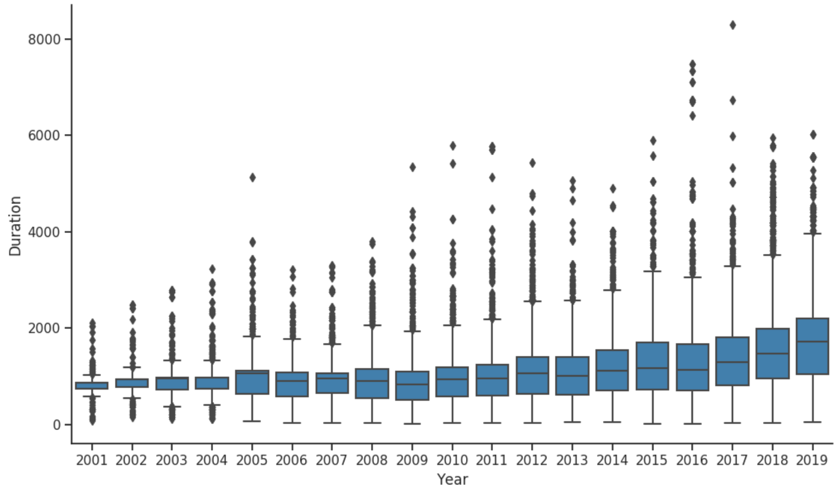

Figure S9. Phase 2 trial duration vs year.

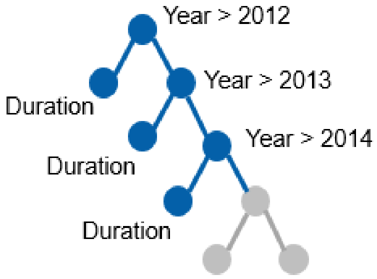

Figure S10. Modeling of the coupling between trial duration and year using a tree model would have required many tree levels.

Figure S11 shows the interaction between trial duration and year was largely decoupled after normalization. The new normalized features were expected to ease the model training process.

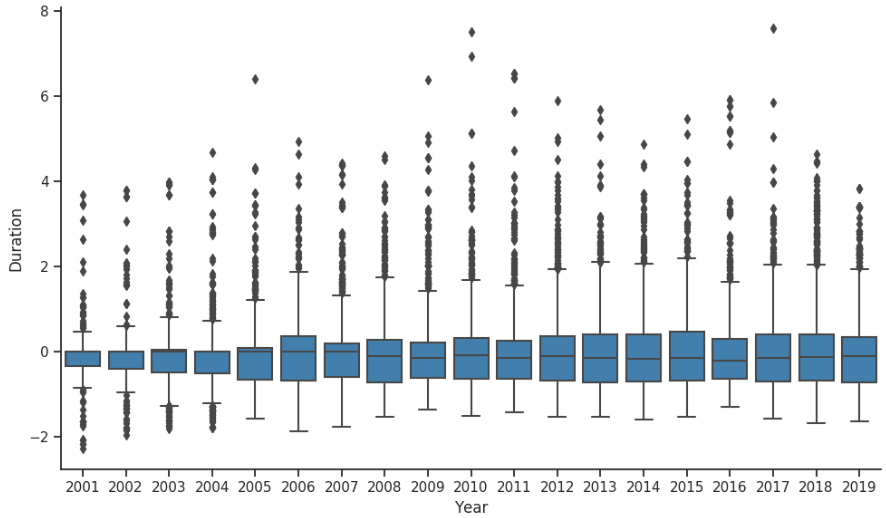

Figure S11. The time-effect on trial duration was minimized (compared to Figure S9) after mean-stdev normalization.

Normalization also tripled the number of feature counts. To avoid feature redundancy, only one of these three correlated feature versions ("*abc"*, "*abc*_norm_by_year", and "*abc*_rank_norm_by_year") was retained based on a feature ranking algorithm described later. Among the final list of retained features, normalized versions were generally favored over their original version, which verified the importance of normalization.

Additional Observations

We observed there were only 30 samples with "intphaseendyear" before 2001, including 14 samples with year 1900 (presumably representing missing data). We overwrote the year to 2001 for all these samples. We also noticed the age units could be in days, weeks, months, and years, therefore, the age value columns need to be standardized to decimal years.

We observed an interesting clustering structure, when "Sponsor p2 positive" was plotted against "Sponsor p2 total" (Figure S12). Sponsors were clearly divided into two clusters, where those sponsors in Cluster B seemed to perform rather poorly for their first 2000 trials, but later were able to boost performance, albeit with slightly lower success rates (smaller slope). Eight-nine percent of the sponsors maintained a relatively high phase 2 success rate throughout the years. This phenomenon remained true when the test dataset was merged. We decided to engineer a binary cluster label as a new feature based on this observation, nevertheless, the new feature did not contribute significantly in the end.

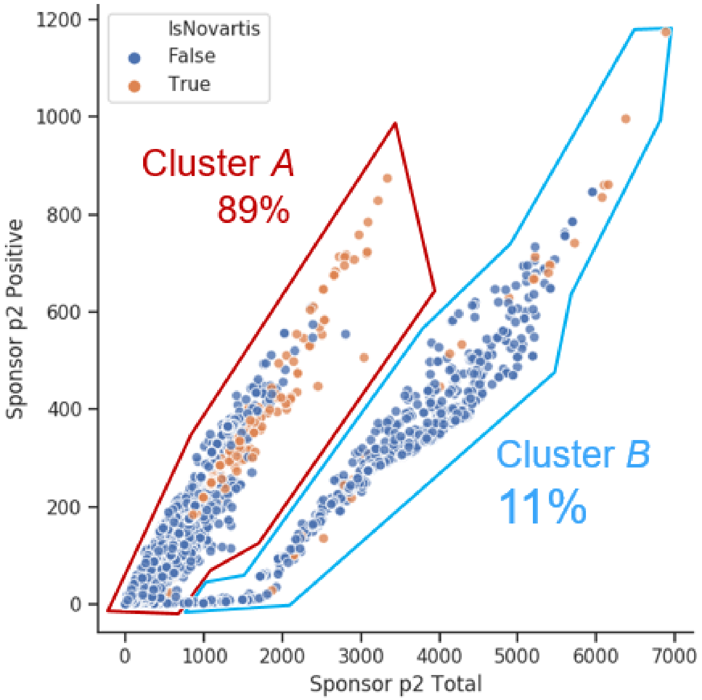

Figure S12. Sponsors fell into two intriguing clusters.

Feature Engineering

Categorical Feature Encoding

There are many categorical features in the PoS dataset, e.g., drug key, indication key, indication group key, sponsor ID, person ID, etc. These are integer identifiers, i.e., their numerical ordering carry no meaning. These features could seriously mislead XGBoost into creating nonsense tree splits, if they were treated as integers. Due to their high cardinality, they should not be treated with one-hot encoding either, otherwise, the resultant large sparse matrices would be hard to learn. Our strategy was to replace these features by their statistics. Considering an example drug key *d* (Figure S13) of a given trial sample completed in year *k*, we collected all trials where *d* was studied and then counted the percentage of successful trials (Figure S13). Success rate was calculated only using samples with year *k*-1 and earlier to avoid any data leaking. As the result, drug key *d* could be encoded with this prior success rate, a continuous and meaning number for XGBoost to split and sort. Similarly, we also encoded drug keys based on phase 3 success rate, completion rate, progress rate, etc.

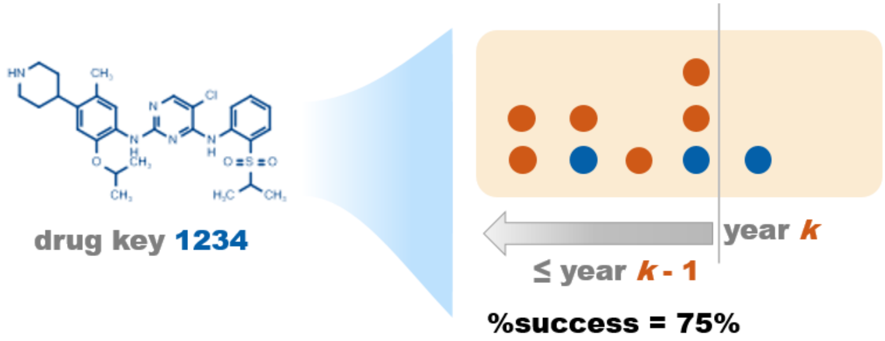

Figure S13. A drug key id 1234 occurred in year *k* can be replaced by its success rate calculated within all records until year *k* - 1.

This encoding technique led to many effective new features. Among all the features provided by the organizer, the feature most correlated with the outcome is called “intpriorapproval” with a Pearson correlation coefficient of 0.28 (p = 0.04). Among our engineered new features, “drug prior trial positice.pct” has a Pearson correlation coefficient of 0.31 ($p < 10^{-10}$), presumably better than all other raw features (Figure S14). Retrospectively, among the top 20 most important features, 7 came from normalization, 9 were newly engineered, and only 4 were from the raw given features (Figure S14). These counts might vary between runs but it showed the value of feature normalization and categorical feature encoding.

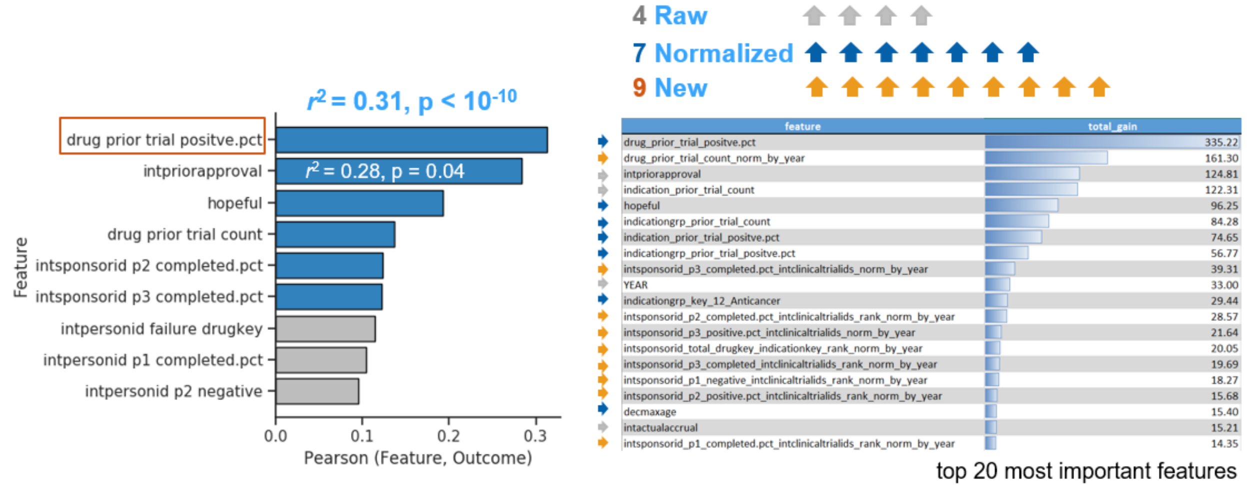

Figure S14. (a) Features most correlated with outcome based on absolute Pearson correlation coefficients. (b) Sources of top 20 features, where the majority came from our feature engineering efforts.

For those multi-label features with fewer discrete values, such as “diseaseType,” we used one-hot encoding. Binary features that were identical for more than 99% of samples were ignored. A total of 607 categories were extracted from 13 multi-label features. Free text columns were transformed into features based on TFIDF (term frequency-inverse document frequency). However, text features did not add much value to our model in terms of score improvement.

Feature Selection

Our experience showed XGBoost could handle several hundred features without problem, we nevertheless applied a feature reduction workflow to remove obviously redundant features generated by normalization or the large number of less informative features derived from text features. The process is illustrated in Figure S15.

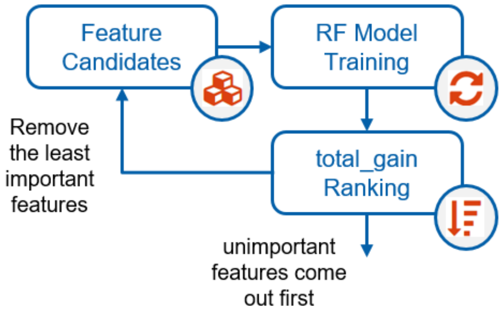

Figure S15. Feature reduction workflow.

We first ranked features by their importance, based on which highly correlated but less important features (Pearson $r^2 > 0.95$) were removed. For feature ranking, we started from all n candidate features, and used them to train a random forest model. We computed the total_gain of each feature and dropped out the feature with the least total_gain. For the remaining n - 1 features, we repeated the previous process to drop out one more feature and n - 2 retained. We iterated until only 1 feature was left. The n features, therefore, were ranked based on the order of their drop out, with the most important features surviving the longest.

For the group of three normalized features, the one with the longest survival was retained. Text features also went through the same triaging process and combined with surviving numerical features for final modeling. We excluded features that might be considered as a leak, e.g., we did not include "generic name," however, post-competition research showed it would have been acceptable. The final model used 275 features. Be aware that random forest, instead of XGBoost, was used in the feature selection process, as its total_gain was considered more reliable since all its tree members were equally important.

Model Evaluation

Cross-validation Strategy

We tested two training-validation splitting strategies. The first strategy was a random five-fold cross validation. The splitting was done in a way that ensured trials sharing the same drug–indication key pair always resided within the same fold and never spanned across training and validation sets. This was to avoid data leaking. The second strategy was to use all drug–indication pairs that did not appear before year 2013 as validation records. Although the second method was a more authentic split mimicking the architecture in the competition (use past to predict the future), it did not perform well compared to the first approach. Presumably, losing the many 2013–2014 records in the training made it hard for the model to capture rules that better described records in the more distant future 2015–2017, as one would expect rules to evolve over the years. Therefore, our final solution adopted the five-fold cross-validation scheme.

Sampling Weighting in Loss Function

We tried to assign weights to trials sharing the same drug–indication pair. We observed there could be many trials studying the same drug–indication simultaneously. For example, erlotinib was studied by about

160 trials (indication 124) in the test set and oxaliplatin was stuided by over 60 trials (indication 141) in the training set (Figure S16). Therefore, we were concerned about overfitting due to some popular drug–indication pairs exerting too much influence during model training. Three trial weighting strategies were tested. First, each trial was weighted by 1. This way a trial–indication pair with m trials contributed m times during the training, a potential bias we would like to supress. Second, each trial was weighted by $1/m$, so each trial–indication pair was counted equally in the loss function. This enhanced the sample diversity. Third, each trial was weighted by $1/\sqrt{m}$, so each drug–indication pair had a weight of $\sqrt{m}$, a compromise between the previous two strategies. The performance varied between training runs and we did not see a statistically consistent advantage of $1/m$ or $1/\sqrt{m}$, compared to the simple weighting scheme, therefore no weight was used.

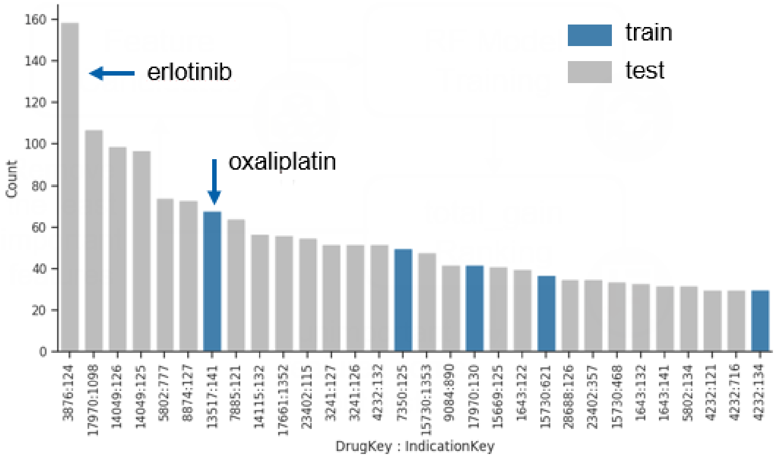

Figure S16. Many drug–indication pairs are heavily studied by multiple trials. Conceptually, this led to bias in training, as well as bias in the leaderboard scoring.

Missing Value Imputation

We also tried two imputation strategies, as this was largely described in the prior publication.[8] The first strategy was to fill the missing value by means. The second was to impute the value using the XGBoost model itself, i.e., we used all other features to build a predictor to predict a target feature containing missing values. Such predictors would use independent features as observed in samples from both training and test datasets. As the XGBoost model can take missing values as its independent variables, unlike random forest models, all independent features other than our target prediction variable, regardless of whether there were missing values, were used in the model training. Although the second strategy had a conceptual advantage, we did not see a consistent difference compared to the first strategy, which was straightforward, thus we chose the first.

Hyperparameter Tuning

The optimal model hyperparameters were identified with grid search based on cross validation. It should be noted that the loss scores used in the search were implemented exactly the same as the final loss function, including all the post processing and augmentation tricks described below.

Model Tuning

Post-processing

Log-loss score is the designated primary leaderboard score. This cross-entropy score heavily penalizes super-confident wrong predictions. For a positive record, improving the prediction probability from 0.99 to $1\text{-}10^{-10}$ only increased the score by 0.01, but reducing the prediction probability from 0.01 to 10^{-10} would decrease the score by 27.6! In other words, there is little reward in making a super-confident correct prediction but there is a huge penalty in making a super-confident wrong prediction. Therefore we clipped all prediction values into range [0.01, 0.99] to avoid severe penalties for mispredictions. The cutoff was determined empirically based on cross validation.

We noticed that 49% of the test records fell into the 2018–2019 period during the model refinement phase. With our domain knowledge, it was clear these samples were extremely unlikely to have positive outcomes, as the time left after the completion of phase 2 trials was too short for a non-trivial approval process. Therefore, we set their probabilities to 0.01.

With feature engineering, five-fold cross validation, and hyperparameter tuning, we were able to build an XGBoost model with the public leaderboard score of 0.275. The text features only had very minor contributions. With the correction of the artifacts in the test set (2018–2019 records), we then obtained a model in the third week with score 0.236.

Drug–indication Augmentation

Our model predicted the probability of success for each trial, i.e., it assigned different probabilities for different trials. However, the truth, the approval/failure outcome, was assigned to each drug–indication pair instead of each trial. The many trials for the same drug–indication pair shared the same outcome truth (Figure S16). This implies we should consolidate the different predicted probabilities for the *m* related trials under the same drug–indication pair into one final probability value.

Mathematically, there is an advantage in unifying the predictions. Considering two related trials share the same positive outcome, with predicted probability $p+\delta$ and $p-\delta$. If we submit them as they are, the score is $\log(p^2-\delta^2)$. If instead we submit their average value for both records, the score is $\log(p^2)$, which is better (the larger, the better here). The conclusion does not change if the outcome is negative.

We did not use average, though. Our reasoning was as long as one of the *m* trials yielded favorable results that successfully demonstrated the efficacy of a drug–indication pair and convinced the authority for an approval, all the remaining *m*-1 trials would take a free ride and be considered successful regardless of how poorly their trials were conducted. Therefore, our aggregation function was an aggressive max() function across the *m* underlying trials. One out of *m* shots was all it took for an approval. With this drug–indication augmentation, our model score improved to 0.222.

Insights

The exact tree structures embedded in XGBoost models were sensitive to small adjustments in the modeling workflow. We hypothesized this was due to two factors: (1) many features are correlated, therefore, models could achieve very similar performance with different decision rules; and (2) the first few trees in the XGBoost models accounted for the most gain, therefore, there would be a signficant variation in the early tree structures in the XGBoost models. Therefore, we decided not to over-interpret the XGBoost model, but instead derived insights from a random forest model consisting of 200 trees. By averaging the decision rules across these equally-weighted trees, we hoped to extract more reliable insights compared to using XGBoost trees.

Single-feature Analysis

The single-feature importance analysis was implemented in scikit-learn module (https://scikit-learn.org). The top ten features are shown in Figure S17. "Hopeful" is an engineered binary feature replacing "termination_reason." "Drug_prior_trial_positive.pct" is an engineered feature described previously in our categorical feature encoding section, representing the historical (up to year *k*-1) success rate of a drug. "Indicationgrp_prior_trial_count" is the number of trials for that indication group up to year *k*-1. "Targetaccrual" (normalized by year) is a normalized version of "targetaccrual." "Indication_prior_trial_count" is self explanatory. "Priorapproval" is a binary feature provided as it is, which had the highest Pearson correlation coefficient with outcome in the original feature set. "Sponsorid_total_drugkey_indicationkey" is the sum of approval and failure counts. "Minage" is the minimal age of patients. "Actualaccrual" is a feature provided. "Indication_prior_trial_positive.pct" reflects the historical success rate for that indication up to year *k*-1.

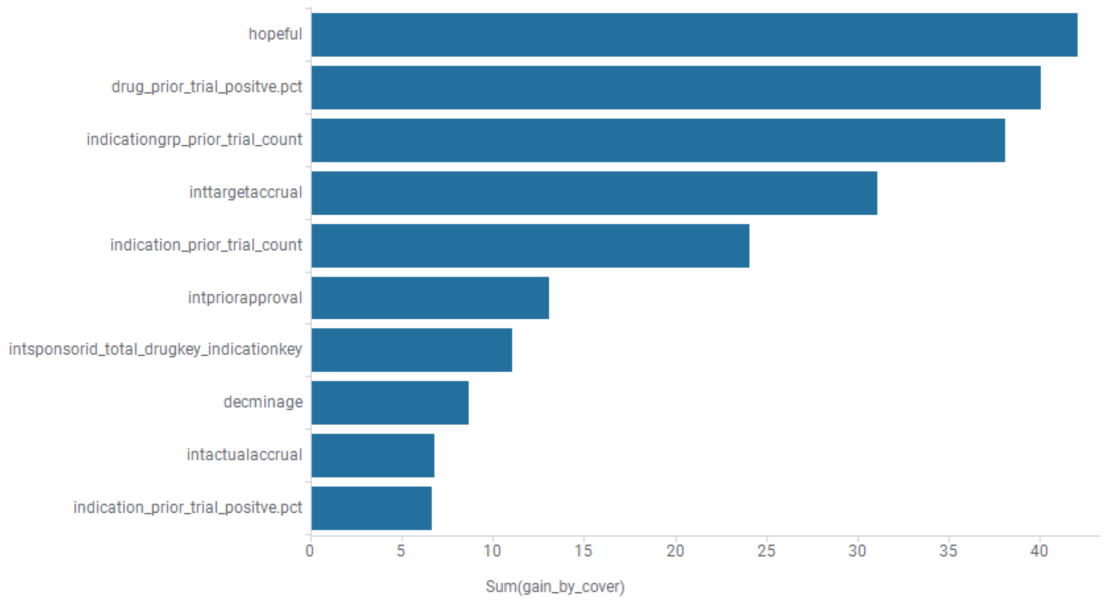

Figure S17. Top 10 most important features based on a random forest model.

As an example, Figure S18 illustrates why "drug_prior_trial_positive.pct" is an important feature. For those drugs with a prior positive rate below 0.48, there is only a 17% chance of a positive outcome versus a remarkable 63%, when the positive rate is above 0.48. The outer ring shows the percentage, if this prior positive rate were irrelevant.

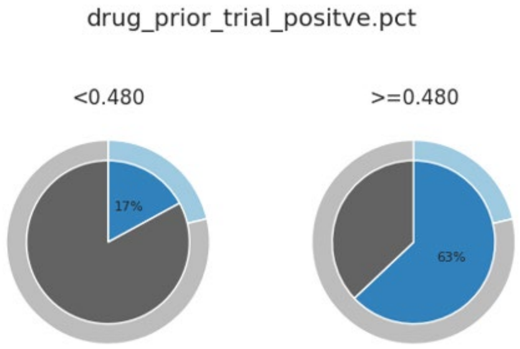

Figure S18. Effectiveness of drug prior positive rate in predicting outcomes.

Figure S19 suggests those trials accepting younger patients (< age 14) tend to be more successful. This might be because companies are generally much more conservative, when it comes to drugs that will be used in kids and sometimes even in infants.

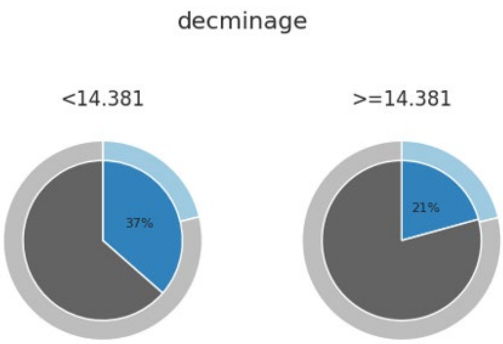

Figure S19. Minimum age of patient in trials can be predictive.

Informative Feature Pairs

As single feature analysis mostly reproduced previously-published results, we aimed to analyze the effect of feature pairs. Similar to analyzing a single feature, we looked at the frequency and the loss contribution of an immediate parent–child feature pair and compiled statistics based on their contributions to the model (as illustrated in Figure S20). This was an idea extended from but not yet implemented in scikit-learn.

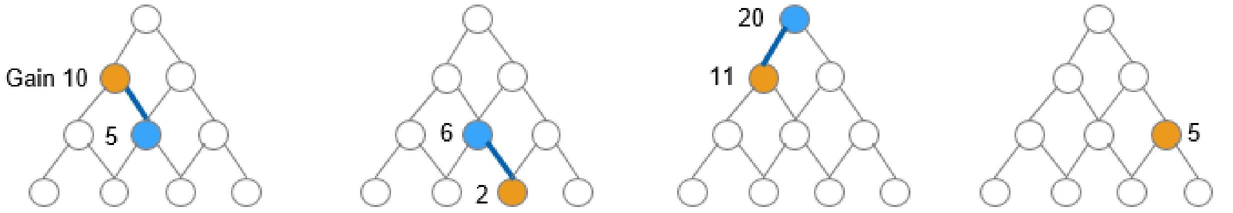

Figure S20. Similar to single feature analysis, a potentially interesting feature pair is parent–child nodes that appear frequently and make important contributions to the gain among a decision forest. The example pair occurs in three out of four trees with an average gain of 13.5.

The network in Figure S21 provides a visual summary of our findings. This network includes all top ten features, as well as some additional features that were not the most important by themselves but became useful in combination with the top features.

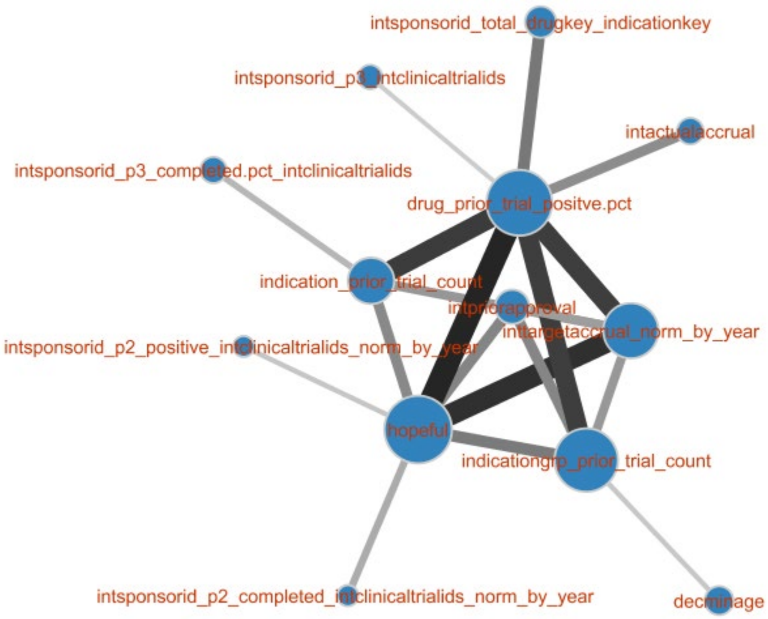

Figure S21. The network of important single features and feature pairs. The size of the node represents the importance of a single feature and the thickness of an edge represents the importance of a feature pair.

Most important feature pairs are intuitive. For example, “hopeful” and “drug_prior_trial_positive.pct” can boost the confidence in a positive outcome. As shown in Figure S22, “drug_prior_trial_positive.pct” alone can predict a positive outcome with 63% accuracy (lower-left pie) and a negative outcome with 83% accuracy (mid-left pie) based on a cutoff of 0.49. However, if this feature were combined with the “hopeful” feature, the model could further improve the accuracy to 70% (lower-right pie) and 96% (center pie), respectively, albeit the percentage of records in these two groups are only 7% and 21% (shown in upper-left corner), respectively. This shows that the combination of two correlated features can lead to higher prediction power.

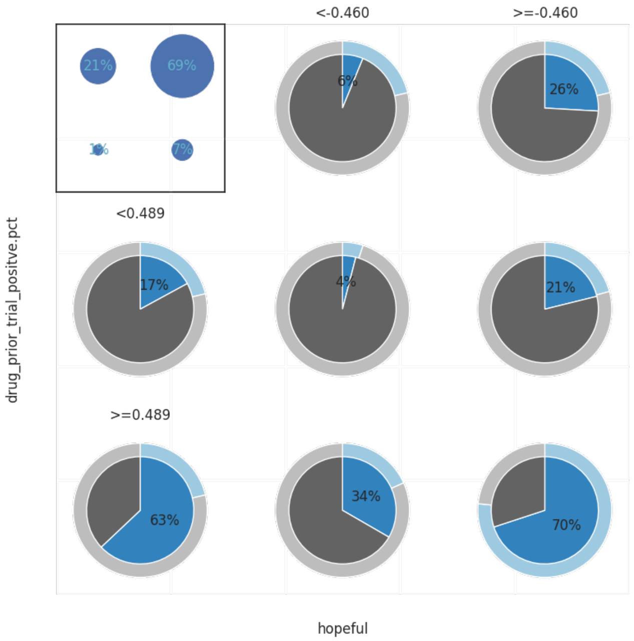

Figure S22. The combination of hopeful and drug prior positive rate can boost prediction.

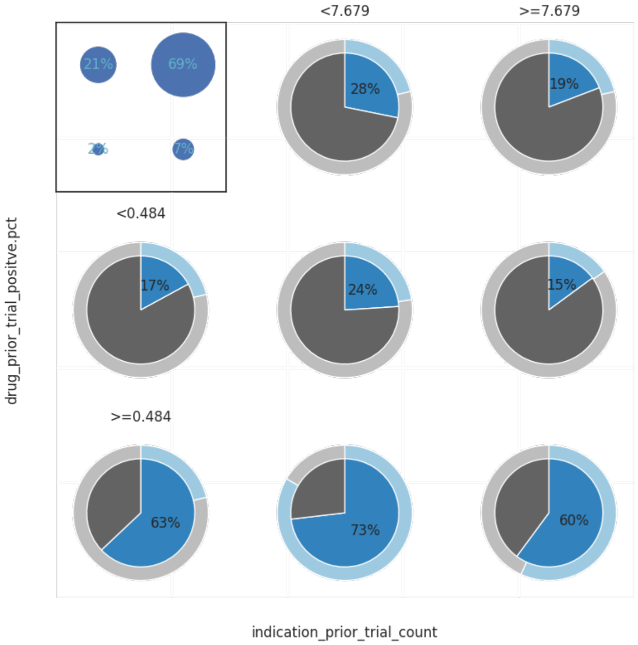

Figure S23. The combination of prior indication trial count and drug prior positive rate can give higher accuracy for applying historically successful drugs in indications that are less explored.

Figure S23 shows that the indications with fewer prior trial counts tend to have a higher success rate (28% vs 19% in the top row). When this is coupled with “drug_prior_trial_positive.pct,” it boosts the accuracy from 63% to 73%.

Although not shown in Figure S21, the combination of “drug_prior_trial_positive.pct” and “anticancer” leads to interesting results summarized in Figure S24. Anti-cancer alone is not a powerful feature, as the positive outcome rate is about the same—21% for “anitcancer” and 22% for “other” indications (top row). However, when we combined it with “drug_prior_trial_positive.pct,” this feature became impactful. The positive outcome rate changes from 65% (lower-left pie) into 54% (lower-middle pie) and 71% (lower-right pie). There appears to be a strong coupling between the two features. Our interpretation is that drugs that have been approved in other indications tend to be more likely to be approved for a new indication, and this empirical rule seems to be more true for cancer than other indications. Presumably, chemotherapies that have already worked in a particular cancer type have a better chance to work in another cancer type. Past history is less likely to predict futrue success for non-cancer indications.

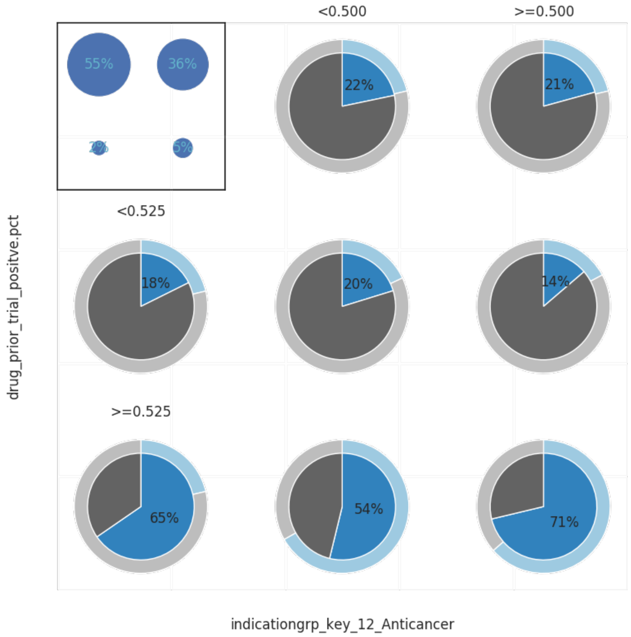

Figure S24. Cancer indication alone is not predictive, but it helps boost accuracy when coupled with drug_prior_trail_positive.pct. This pair is not among the top, probably because the number of records benefiting from this rule is small.

References

1. Chen, T., and Guestrin, C. (2016). XGBoost: A scalable tree boosting system. Proceedings of the ACM SIGKDD International Conference on Knowledge Discovery and Data Mining, 785–794. 10.1145/2939672.2939785.

2. Imai, K., and Ratkovic, M. (2014). Covariate balancing propensity score. Journal of the Royal Statistical Society: Series B: Statistical Methodology 76, 243–263. 10.1111/rssb.12027.

3. Brest, J., Greiner, S., Bošković, B., Mernik, M., and Zumer, V. (2006). Self-adapting control parameters in differential evolution: A comparative study on numerical benchmark problems. IEEE Transactions on Evolutionary Computation 10, 646–657. 10.1109/TEVC.2006.872133.

4. Thakur, A. (2020). Approaching (Almost) Any Machine Learning Problem. (Abhishek Thakur).

5. Pargent, F., Pfisterer, F., Thomas, J., and Bischl, B. (2021). Regularized target encoding outperforms traditional methods in supervised machine learning with high cardinality features. arXiv, arXiv:2104.00629.

6. Slakey, A., Salas, D., and Schamroth, Y. (2019). Encoding Categorical Variables with Conjugate Bayesian Models for WeWork Lead Scoring Engine. arXiv, arXiv:1904.13001.

7. Gore, S. M. (1987). Biostatistics and the medical research council. Medical Research Council News 35, 19–20.

8. Lo, A. W., Siah, K. W., and Wong, C.H. (2019). Machine learning with statistical imputation for predicting drug approval. Harvard Data Science Review 1. 10.1162/99608f92.5c5f0525.
